# Development of Zalfermin, a Long-Acting Proteolytically Stabilized FGF21 Analog

**DOI:** 10.1021/acs.jmedchem.4c00391

**Published:** 2024-07-16

**Authors:** Kristian Sass-Ørum, Tina Møller Tagmose, Jørgen Olsen, Annika Sjölander, Per-Olof Wahlund, Dan Han, Andreas Vegge, Steffen Reedtz-Runge, Zhe Wang, Xiang Gao, Birgit Wieczorek, Kasper Lamberth, Kirsten Lykkegaard, Peter Kresten Nielsen, Henning Thøgersen, Mingrui Yu, Jianhua Wang, Jørn Drustrup, Xujia Zhang, Patrick Garibay, Kristian Hansen, Ann Maria Kruse Hansen, Birgitte Andersen

**Affiliations:** † Novo Nordisk A/S, Global Research Technologies, DK-2760 Maaloev, Denmark; ‡ Novo Nordisk A/S, Global Drug Discovery, DK-2760 Maaloev, Denmark; § Novo Nordisk A/S, Novo Nordisk Research Center China, Beijing 102206, China

## Abstract

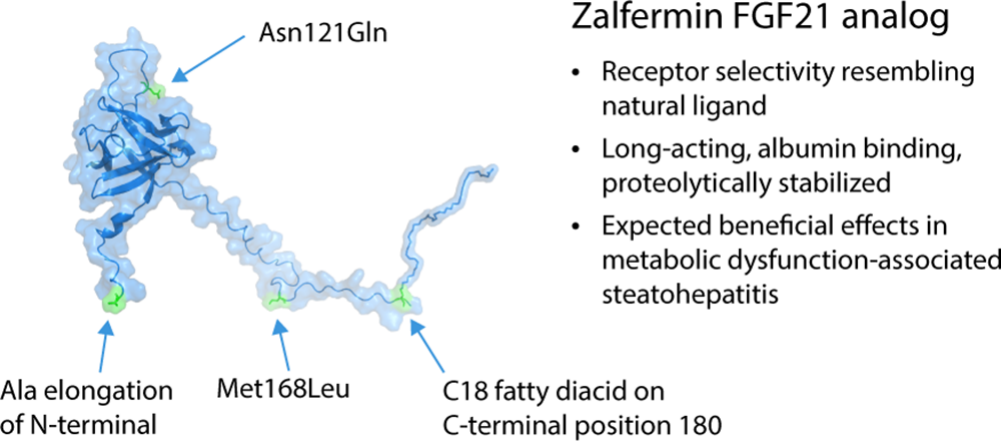

Here, we describe the development of the FGF21 analog zalfermin (NNC0194-0499, **15**), intended for once-weekly sc dosing. Protein engineering was needed to address inherent druggability issues of the natural FGF21 hormone. Thus, deamidation of Asp121 was solved by mutation to glutamine, and oxidation of Met168 was solved by mutation to leucine. N-terminal region degradation by dipeptidyl peptidase IV was prevented by alanine residue elongation. To prevent inactivating metabolism by fibroblast activation protein and carboxypeptidase-like activity in the C-terminal region, and to achieve *t*_1/2_ extension (53 h in cynomolgus monkeys), we introduced a C18 fatty diacid at the penultimate position 180. The fatty diacid binds albumin in a reversible manner, such that the free fraction of zalfermin potently activates the FGF-receptor complex and retains receptor selectivity compared with FGF21, providing strong efficacy on body weight loss in diet-induced obese mice. Zalfermin is currently being clinically evaluated for the treatment of metabolic dysfunction-associated steatohepatitis.

## Introduction

FGF21 was identified as a potential therapeutic protein in 2005, based on its ability to increase glucose uptake into 3T3-L1 adipocytes and to reduce blood glucose in diabetic mice.^1^ In 2007, the antidiabetic effect of FGF21 was confirmed in diabetic monkeys.^2^ FGF21 is an 181 amino acid protein mainly secreted from the liver,^3^ belonging to the FGF19 subfamily of endocrine FGFs (FGF19, FGF21, and FGF23). In contrast to paracrine FGFs, endocrine FGFs bind heparin poorly,^4^ and can therefore escape the cellular matrix and enter the circulation.^5^ FGF21 signaling requires the c-isoform of fibroblast growth factor receptor 1 (FGFR1c), 2 (FGFR2c) or 3 (FGFR3c), the transmembrane coreceptor beta-klotho (KLB), and heparin.^6−8^ KLB consists of an extracellular domain, a single-pass transmembrane region, and a short cytoplasmic tail. KLB does not induce intracellular signaling, but the extracellular domain serves as a docking site facilitating the interaction between FGF21 and the FGFRs. The N- and C-terminal regions of FGF21 are critical for binding to FGFR1c/FGFR3c and KLB, respectively.^9,10^ The FGF21-receptor complex is expressed in adipose tissue, the liver, the pancreas, and specific areas of the CNS.^11,12^ In addition to the antidiabetic effect, preclinical data have shown pharmacological doses of FGF21 to lower plasma insulin, triglycerides, cholesterol, liver fat, and body weight.^13−15^ This led to high expectations for a novel and efficacious treatment of obesity, T2D, dyslipidemia, and metabolic-disfunction-associated steatohepatitis (MASH).

The development of FGF21 for medical use must address a wide variety of factors, such as production cost, chemical stability, PK, pharmacological efficacy, immunogenicity risk, and formulation properties. To date, seven optimized FGF21 analogs have entered clinical testing: LY2405319, PF-05231023, BMS-986036 (pegbelfermin), AKR-001 (efruxifermin), BOS-580 (formerly LLF580), BIO89-100 (pegozafermin), and the FGF21 analog zalfermin (NNC0194-0499, **15**). Moreover, two FGF21-receptor agonistic antibodies have been tested in humans.^16,17^

LY2405319 was the first FGF21 analog to be clinically tested.^18^ An additional disulfide bond (Leu118Cys and Ala134Cys) stabilized this compound for which a preserved multiuse formulation for once-daily dosing was developed. Deletion of the four N-terminal amino acids, His-Pro-Ile-Pro, mitigated inhomogeneous compound quality resulting from partial cleavage by the *Pichia pastoris* expression host and further abolished human metabolism in the same region.^19,20^ Ser167Ala mutation prevented O-linked glycosylation during expression.^21^ In mice, the potency of LY2405319 was on par with FGF21.^21^ However, in nonhuman primates (NHP), high doses (3, 9, and 50 mg/kg) of LY2405319 were required to lower blood glucose^22^ compared to FGF21 (0.03–0.1 and 0.3 mg/kg).^2^ LY2405319 was tested in people with obesity and T2D, and after 4 weeks of daily treatment the two highest doses of 10 and 20 mg lowered plasma triglycerides (TG) by 50% while no blood glucose-lowering effect was observed.^18^

The systemic *t*_1/2_ of iv- and sc-administered FGF21 is 2.0 and 4.3 h in cynomolgus monkeys, respectively,^2^ and one sc daily dose of FGF21^2,15^ and LY2405319^18^ is required for PD effect. Accordingly, several *t*_1/2_ extension strategies, such as antibody conjugation (so-called CovX-body format),^23^ fragment crystallizable (Fc)-fusion,^24^ and PEGylation,^12,15^ have been explored.

The first approach to increase *t*_1/2_ came with the FGF21 analog PF-05231023 (also known as CVX-343). This FGF21 CovX-body conjugate compound consists of two engineered (des-His1, Ala129Cys) FGF21 molecules which, via the mutant Cys residue, are covalently linked to the fragment antigen-binding (Fab) of an IgG1 monoclonal antibody.^23^ Administration of a single high (200 mg) iv dose of PF-05231023 to subjects with T2D lowered plasma TG 40–50% from day 4–14.^12^ However, mean terminal *t*_1/2_ of 6.5–7.7 h and 66.5–96.6 h for intact C- and N-termini, respectively, indicated rapid cleavage in the FGF21 C-terminal region.^12^ Protection against C-terminal cleavage via attachment of the CovX-body Fab to the C-terminal of FGF21 was not feasible, since that compound showed more than 200-fold loss of potency in vitro.^23^

Pegbelfermin (BMS-986036) is an FGF21 analog with extended *t*_1/2_ obtained by attachment of a 30-kDa PEG moiety to a non-natural amino acid para-acetyl phenylalanine at position 108.^25,26^ The selection of position 108 followed a scan of the potential modification sites. Here the position 108 PEGylated protein was shown to retain potency in vitro, and in mice and in sc-dosed rats *t*_1/2_ was increased more than 10-fold.^26^ In contrast, pegbelfermin only showed a *t*_1/2_ of 19–24 h in sc-dosed humans.^27^ In a phase 2a study, 10 mg dosed once daily decreased absolute liver fat content by 6.8%, whereas 20 mg once weekly decreased absolute liver fat content by 5.2%.^28^ To prepare for phase 3, pegbelfermin was tested in patients with biopsy-confirmed MASH in two phase 2b trials (FALCON 1 and 2), where patients received 10, 20, or 40 mg compound or placebo once weekly.^11,29^ In these clinical studies, pegbelfermin failed to reach the primary outcome of MASH resolution and fibrosis regression.^30,31^

To understand the limited success of early clinical candidates, it is important to consider how FGF21 is metabolized. Circulating endogenous human FGF21 is primarily cleaved by dipeptidyl peptidase IV (DPP-IV) after Pro2 and Pro4, and by fibroblast activation protein (FAP) after Pro171,^19,20^ whereas carboxypeptidase-like protease activity removes 1–3 amino acids in the C-terminal.^32^ The identity of the carboxypeptidase-like protease(s) is not yet known. Importantly, the absence of only a few C-terminal amino acids markedly reduces potency,^9,10^ strongly indicating that therapeutic FGF21 analogs should be C-terminally stabilized. This is particularly important for analogs intended for infrequent dosing such as once weekly.

Efruxifermin (formerly Fc-FGF21[RGE], AMG 876)^32,33^ is a ∼92 kDa compound consisting of two FGF21 analog (Leu98Arg, Pro171Gly, Ala180Glu; hence RGE) moieties fused at their N-termini to an IgG1 Fc domain.^32^ The Pro171Gly mutation serves to prevent C-terminal FAP cleavage and efruxifermin shows prolonged *t*_1/2_ in mice (19 h, iv), cynomolgus monkeys (76 h, iv), and humans (3–3.5 days, sc).^34^ Propensity for aggregation at high concentrations and temperature is mitigated by the Leu98Arg mutation.^33^ The Ala180Glu mutation increases binding to mouse, cynomolgus, and human KLB, enhances potency in a mouse PD model, and reduces C-terminal region carboxypeptidase-like degradation.^32^ However, a comparison of Fc-FGF21(RGE) with Fc-FGF21(RG) effects on metabolic parameters in cynomolgus monkeys suggests the mutation 180E to provide limited additional in vivo efficacy in NHPs.^32^ Efruxifermin has been used in several clinical trials. Recently, a phase 2b trial (24 weeks) showed efruxifermin 28 and 50 mg once weekly to lower fibrosis by 39% and 41%, respectively, compared with 20% for placebo. MASH resolution was achieved in 47% and 76% of patients treated with 28 and 50 mg efruxifermin, respectively, compared with 15% for placebo.^35^

Pegozafermin (BIO89–100) is a glycoPEGylated FGF21 analog with an extended in vivo *t*_1/2_ of 50 h in sc-dosed diabetic cynomolgus monkeys^36^ and a median sc *t*_1/2_ of 46–68 h in people with MASH, as recently reported from a pegozafermin phase 1b/2a study.^37^ Pegozafermin has an N-terminal methionine residue extension, a single 20 kDa linear PEG covalently attached via a glycosyl moiety on a mutant residue in position 172, and a second mutant residue in position 175.^38−41^ Twelve weeks of dosing with 27 mg once-weekly pegozafermin lowered liver fat by 70% in subjects with MASH or phenotypic MASH,^41^ which is on par with 50 mg once-weekly dosing of efruxifermin.^42,43^ Recently, promising data were obtained from a 24-week, randomized, placebo-controlled phase 2b trial in patients with MASH with fibrosis stage F2/F3 where 15 and 30 mg of pegozafermin dosed once weekly resolved MASH by 37% and 23%, respectively, versus 2% in the placebo group, and decreased fibrosis by 22% and 26%, respectively, versus 7% in the placebo group.^44^

BOS-580 (formerly LLF580)^45^ contains two FGF21 analog moieties, both of which are stabilized by an introduced disulfide bond and are fused at their N-termini to the human IgG1 Fc domain. The added disulfide bond reportedly increases the thermodynamic stability and decreases the proteolytic lability of the FGF21 moiety.^45^ This FGF21 analog has been tested in adults with obesity with mild hypertriglyceridemia who were treated for 12 weeks with three injections of BOS-580 given every fourth week. This led to a 50% decrease in plasma TG and a 60% decrease in hepatic liver fat content,^45^ suggesting a once-monthly dosing potential of BOS-580.

The improvement in PK properties has led to FGF21 analogs which support weekly or less frequent sc dosing. In the clinical setting, the FGF21 analogs have been well tolerated, and the main side effects are nausea and diarrhea. However, longer studies are required to determine long-term safety as preclinical side effects (hypothalamus−pituitary−adrenal axis, blood pressure, bone health, and female fertility) have been reported.^46−48^ While the clinical efficacy on blood glucose and body weight of the various FGF21 analogs has been rather disappointing, significant and meaningful effects have been obtained on plasma lipids and MASH parameters. Efruxifermin has paved the way by establishing the potential role of FGF21 in the treatment of MASH, with an effect on MASH resolution and fibrosis improvement after just 24 weeks in patients with MASH with fibrosis score 2/3,^43,49^ leading to high expectations for the FGF21 drug class in MASH, a progressive disease with no current treatment options.^50^ Efruxifermin, pegozafermin, BOS-580, and zalfermin are currently being tested in phase 2 clinical MASH trials.

Here, we describe the development of once-weekly sc zalfermin (NNC0194-0499, **15**). Compared with native FGF21, zalfermin has improved chemical and metabolic stability, natural FGFR selectivity, low immunogenicity risk, and biophysical and formulation properties supporting long-term refrigerated storage and ambient in-use time. At the initiation of our work, PEGylation, antibody conjugation (CovX-body), and Fc-elongation in the FGF21 C-terminal region had been described in multiple cases to severely compromise biological activity^23,26,33^ due to steric conflict with KLB coreceptor binding. To solve this, we pursued *t*_1/2_ extension via a lipid side chain that can reversibly bind to albumin in the bloodstream (reviewed by Kurtzhals et al.^50^). The strategic positioning of the albumin binder in the C-terminal offers intact biological activity (of the fraction of FGF21 analog not bound to albumin) and protection against protease activity (via the FGF21 analog fraction bound to albumin).

## Results and Discussion

### Screening Plan

In order to develop **15**, we first screened for intrinsic FGF21 druggability limitations. Storage of protein pharmaceuticals can cause degradation such as oxidation of Met, deamidation of Asn and Gln, and isomerization of Asp,^51−53^ hence, we performed protein chemical and biophysical characterization of human FGF21 with N-terminal Met extension (Met-FGF21, **1**) and selected analogs before and after forced degradation. The forced degradation was done by subjecting liquid solutions of Met-FGF21 and selected analogs to prolonged storage at various temperatures, pH buffers, and excipient compositions. These studies helped us identify backbone mutations that should be considered for **15**.

Our strategy to use a lipid side chain for extending the in vivo *t*_1/2_ necessitated semirecombinant FGF21 analog production. Chemical polypeptide modifications are most often performed by acylation of the N-terminal primary amino group or of the epsilon amino group of lysine residues; however, the presence of four lysine residues in FGF21 prevented site-specific conjugation. Instead, conjugation to mutant cysteine residues was found to be selective and efficient. For this purpose, we prepared albumin-binding fatty-diacid side chains functionalized with bromoacetamide for thiol alkylation. Besides the fatty diacid, the side chains contain a gamma-glutamic acid (gGlu) linker, a hydrophilic spacer composed of two 2-(2-(2-aminoethoxy)ethoxy)acetyl elements (OEG-OEG), an ethylenediamine (C2DA), and finally an acetyl moiety derived from bromoacetyl (Ac–Br), used as the Cys-reactive group. In general, we did not observe interference with the two naturally occurring cysteine residues that form a disulfide bond in FGF21. Some cysteine-containing analogs were susceptible to degradation and dimerization during screening-scale production. In-process cysteamine protection of the introduced cysteine (see Experimental Section) in many cases solved these challenges, but still some compounds were abandoned since they did not meet screening-phase quality criteria. Taken together, these findings prompted us to first screen for potential side chain modification sites, and then to investigate compounds containing various fatty-diacid side chains on such sites. We screened for a variety of side chains, some of which are mentioned elsewhere.^54^

In vitro potency of FGF21 analogs was determined in HEK293 cells stably transfected with human KLB and relying on endogenous expression of FGF receptors. FGF21 analogs induce phosphorylation of ERK in these cells^55^ and the potency of native FGF21 is approximately 1 nM.^1^ FGF21 binds the Ig-like binding domains of FGFR1c (D2/D3)^56^ in the presence of KLB.^7^ The D2/D3 domains are >99% sequence identical between mice, pigs, cynomolgus monkeys, and humans,^8^ thus the endogenous human FGFRs are used in our species qualification assays. In contrast, the sequence identity of the extracellular domain of human KLB ranges from 97% (cynomolgus monkeys), to 87% (pigs^57^), to 79% (mice). For species qualification, the potency of **15** and Met-FGF21 (**1**) was therefore compared in HEK293 cells overexpressing mouse, cynomolgus monkey, or human KLB. To complement the functional assay, we tested selected analogs in a binding assay based on AlphaScreen technology comprising both KLB and the extracellular domain of FGFR1c.^55^

We expected the reversible albumin binding by C-terminal region fatty-diacid side chains to impact KLB binding and thus the potency. To help guide the selection of side chain moiety, the in vitro potency in a range of albumin concentrations was determined for selected FGF21 analogs.

The potential impact of FGF21 engineering on FGFR subtype specificity was determined in vitro using Ba/F3 cells overexpressing FGFR1c/KLB, FGFR2c/KLB, FGFR3c/KLB, and FGFR4/KLB. FGF21 was used as a comparator, while FGF1 and FGF19 served as assay controls for signaling through the FGFR2c/KLB and FGFR4/KLB receptor complexes, respectively. To complement these functional assays, binding assays utilizing the ectodomain of FGFR1c, FGFR2c, FGFR3c, and FGF4 together with KLB were applied.

Mouse was selected as the primary species for initial PK screening since in-house metabolism data showed the same in vivo degradation products of Met-FGF21 in mice and minipig (Supporting Information A [Figure S1]). This was in accordance with published data on the FAP metabolism of FGF21^19,20^ and data for Fc-FGF21 in cynomolgus monkeys with respect to carboxypeptidase-like degradation.^32^ An antihuman FGF21 enzyme-linked immunosorbent assay (ELISA) kit was used for measuring FGF21 and metabolite identification was done with LC–MS. Detailed PK characterization of **15** was performed using iv and/or sc dosing to minipigs, Landrace-Yorkshire Duroc (LYD) pigs, and cynomolgus monkeys.

Body-weight loss in mice provided a noninvasive means to discriminate in vivo potency of FGF21 analogs. The results should be cautiously interpreted since: (i) FGF21 binds 2–3-fold stronger to mice compared with human KLB in the presence of FGFR1c,^55^ hence the body-weight losses in mice may be overestimated, and (ii) despite remarkable body-weight-lowering effects of FGF21 analogs in mice,^13^ pigs,^14,57^ and NHP,^15,22,58^ these results do not translate well to humans.^18,43,59,60^ However, FGF21 analogs with body-weight-lowering effect in mice^32^ have shown great effects on MASH resolution and fibrosis regression in clinical trials.^43,60^

In order to assess the in vivo metabolism of **15**, two sets of experiments were conducted. In vitro assays determined whether the C-terminal region of **15** during the presence of albumin was protected against FAP cleavage. Furthermore, the metabolism of **15** in mice and minipigs was characterized by LC–MS analysis of PK study plasma samples.

The final **15** drug product development will be completed upon the availability of outcomes from ongoing clinical trials. However, to pave the way for the drug product, the biophysical, chemical, and formulation properties of **15** were characterized in a liquid formulation compatible with sc injection. Forced stability experiments combined with Arrhenius calculations were used to indicate meaningful drug-product shelf life and in-use time.

### Asn121Gln Mutation to Prevent Deamidation

**Figure 1:**
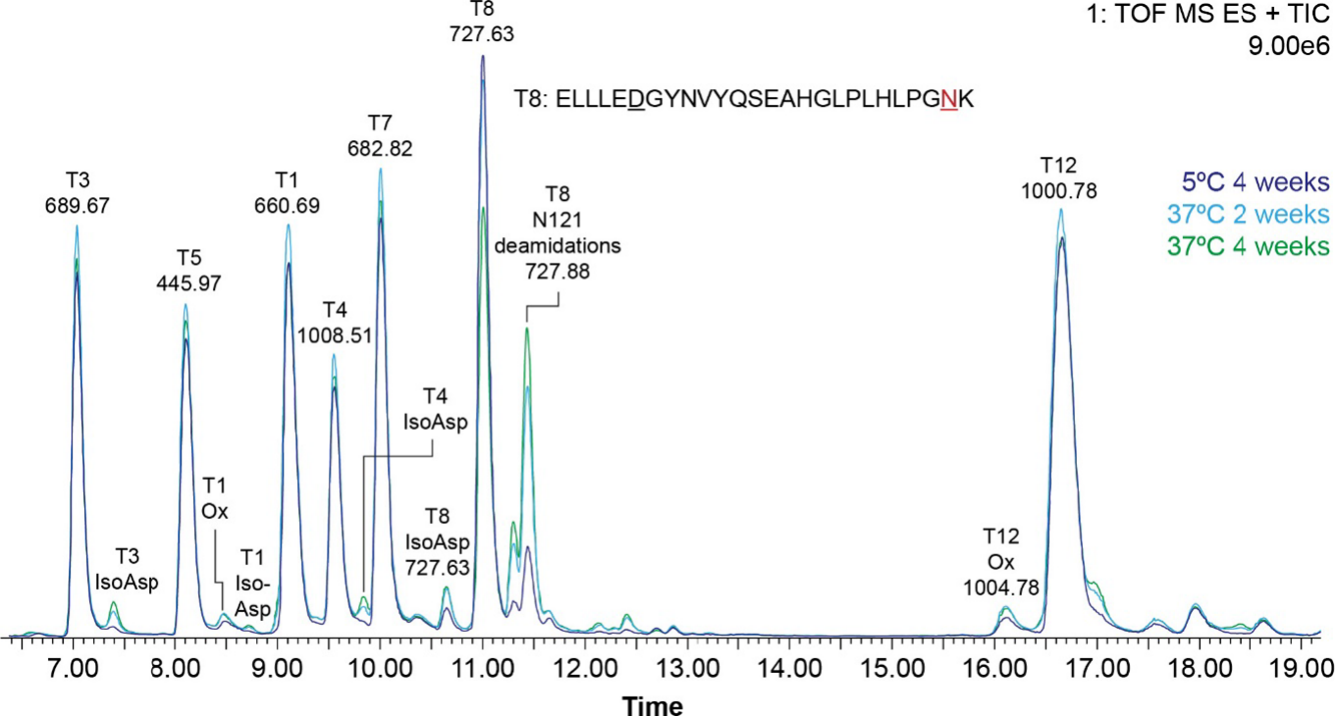
Tryptic peptide map of met-FGF21 (**1**) (10 mg/mL in 10 mM phosphate buffer pH 8.2) stored at 37 °C for 2 and 4 weeks. The amino acid sequence of tryptic peptide T8, which contains Asn121, is shown (*Z* = 4). Peptides were identified by LC–MS and liquid chromatography elevated energy mass spectrometry (LC–MS^E^). Met-FGF21: human FGF21 with an N-terminal Met extension.

**Figure 2:**
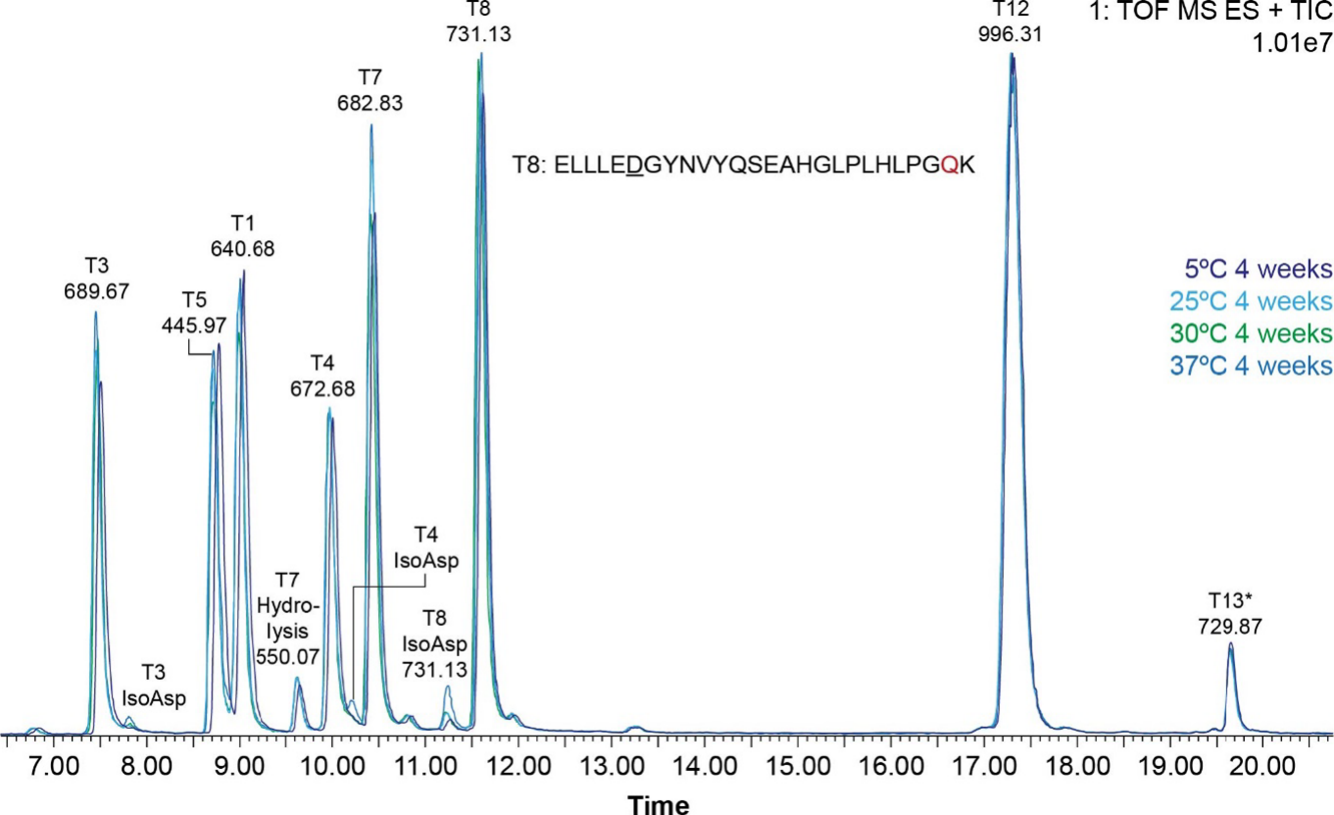
Tryptic peptide map of **15** (15 mg/mL in 10 mM phosphate buffer pH 8.2 with 2% glycerol) stored at 5, 25, 30, and 37 °C for 4 weeks. Peptides were identified by LC–MS and liquid chromatography elevated energy mass spectrometry (LC–MS^E^).

Forced degradation followed by tryptic peptide mapping and LC–MS (Figure 1) revealed temperature-dependent deamidation (+1 Da) of Asn121 (more than 30% after 4 weeks at 37 °C) in a liquid formulation of Met-FGF21 (**1**). To mitigate this, the Asn121Gln mutation was introduced in **15** which was confirmed to be devoid of deamidation (Figure 2).

### N-Terminal Alanine Elongation (−1Ala) and Met168Leu Mutation to Prevent Oxidation

Tryptic peptide mapping after Met-FGF21 (**1**) storage in phosphate buffer revealed oxidation to occur in −1Met and Met168 (see Figure 1, tryptic peptides T12 and T1, respectively). We estimate this oxidation to occur at the level of a few percent per month. Accurate quantitation was hindered by the trypsin cleavage reaction itself contributing to oxidation. To prevent methionine oxidation of **15**, the N-terminal was designed with an alanine residue instead of methionine and Met168 was mutated to leucine.

### –1Ala Prevents DPP-IV Cleavage

The N-terminal of endogenous FGF21 (His-Pro-Ile-Pro, HPIP) is cleaved by DPP-IV to release His-Pro and subsequently Ile-Pro.^19^ DPP-IV preferentially cleaves off N-terminal dipeptides if the substrate has a Pro or an Ala in the P1 position,^61^ whereas substrates with a Pro in the P1′ position are generally resistant to DPP-IV hydrolysis. Recombinant *E. coli* expression of human FGF21 adds an N-terminal residue which brings a Pro into the P1′ position. We sought to confirm that the −1Ala design of FGF21 offers protection toward DPP-IV proteolysis. Thus, LC–MS was used to monitor DPP-IV cleavage (in vitro) of two truncated peptides representing 1–31 (present in endogenous FGF21) and −1–31 (−1Ala). 1–31 had a *t*_1/2_ of 41 min, whereas −1–31 was stable toward DPP-IV degradation (see Table 1). 1–31 was cleaved by DPP-IV and two metabolites representing a loss of first His-Pro and subsequently Ile-Pro to form 3–31 and 5–31, respectively, were identified (see Supporting Information B [Figure S2]).

**Table 1:** N-Terminal Alanine Elongation Protects the N-Terminal of FGF21 against Dipeptidyl Peptidase IV Cleavage

peptide fragment	*t*_1/2_ (min)
1–31: HPIPDS···.QTEA	41
–1–31: AHPIPDS···.QTEA	stable

### Isomerization-Prone Natural Aspartate Residues Retained

FGF21 contains 11 aspartic acid residues, which have the potential to isomerize. Isomerization of l-aspartate is a well-known phenomenon^52^ and can lead to three products: d-aspartate, l-isoaspartate, and d-isoaspartate. Asp102, Asp38, Asp25, and Asp5 isomerize primarily to their l-isoaspartate form in a temperature-dependent manner. The isomerization was characterized by trypsin peptide mapping in combination with LC–MS (Figure 2 and Supporting Information H) and degradation products quantitated from the peptide mapping UV signal and Arrhenius calculations (5, 25, 30, and 37 °C). Isomerization rates were accelerated by temperature; thus, Asp102 isomerized to 1.0% after 1 month at 30 °C but only 0.1% after 2 years at 4 °C (extrapolation from Arrhenius calculations). Other isomers (d-isoaspartate and d-aspartate) were detected, albeit at a much lower level. In line with structural models suggesting Asp102 is located in an extended loop, which is important for FGF-receptor complex interaction (data not shown), mutational efforts to prevent isomerization typically resulted in the loss of FGF21 activity and frequently also a change of FGF-receptor selectivity (data not shown). Accordingly, the natural Asp residues were retained.

### Stabilizing Mutations in the Backbone of **15**

**Figure 3:**
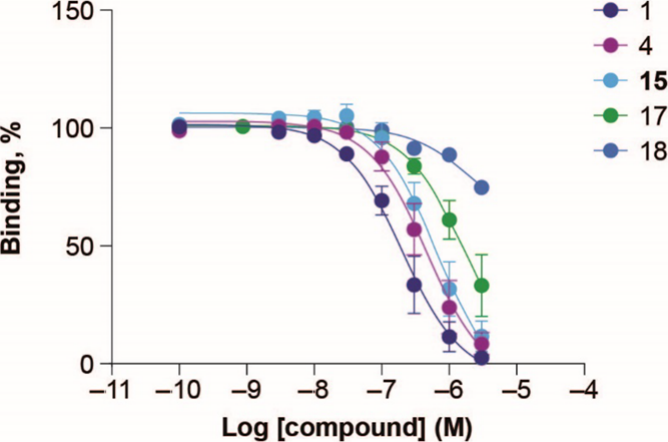
FGFR1c/KLB AlphaScreen binding data for compounds **1**, **4**, **15**, **17**, and **18**. Data are mean ± SEM (*n* = 4–7).

Taken together, stabilization of the **15** backbone was achieved by introducing −1Ala elongation and Asn121Gln and Met168Leu mutations. These mutations were well tolerated and only had a minor effect on potency, as evident when comparing the stabilized backbone (**4**) of **15** to **1** in HEK293-/KLB-expressing cells (2.0 vs 1.6 nM) (Table 2 and Supporting Information C [Table S1]). A small but significant loss of binding affinity to FGFR1c/KLB was observed for **4** as compared to **1** (pIC_50_ 6.18 ± 0.10 vs 6.56 ± 0.89, *p* = 0.017; Figure 3 and Supporting Information C [Table S2]).

**Table 2:** Structure–Activity Relationship of C-Terminal Region FGF21 Analogs^a^

compound	mutations^b^	modification		potency (EC_50_, nM)
		site	type	
FGF21 (**0**)				1.0
**1**	–1M			1.6
**2**	–1A			1.3
**3**	–1A, 121Q			1.0
**4**	–1A, 121Q, 168L			2.0
**5**	–1A, 71C, 121Q, 168L	71C	fatty diacid^c^	4.8
**6**	–1A, 121Q, 167C, 168L	167C	cysteamine	1.4
**7**	–1A, 121Q, 168L, 170C	170C	cysteamine	3.4
**8**	–1A, 121Q, 168L, 171C	171C	cysteamine	2.2
**9**	–1A, 121Q, 168L, 172C	172C	cysteamine	3.3
**10**	–1A, 121Q, 168L, 174C	174C	fatty diacid^c^	1.5
**11**	–1A, 121Q, 168L, 174C	174C	cysteamine	6.1
**12**	–1A, 121Q, 168L, 176C	176C	cysteamine	160
**13**	–1A, 121Q, 168L, 178C	178C	cysteamine	90
**14**	–1A, 121Q, 168L, 179C	179C	cysteamine	67
**15**	–1A, 121Q, 168L, 180C	180C	fatty diacid^c^	3.3
**16**	–1A, 121Q, 168L, 180C	180C	cysteamine	2.5
**17**	–1A, 121Q, 168L, 180C, des181	180C	fatty diacid^c^	3.9
**18**	–1A, 121Q, 168L, 181C	181C	fatty diacid^c^	39
**19**	–1A, 121Q, 168L, 181C	181C	cysteamine	24

a The stabilized backbone (**4**) is included, and FGF21 (**0**) and Met-FGF21 (**1**) serve as references. In vitro potency (in the absence of HSA) was measured as the phosphorylation of ERK in HEK293 cells transfected with human beta-klotho. The complete data table with pEC_50_ values, number of experiments, and standard deviation is reported in Supporting Information C [Table S1].

b –1A and −1M are single amino acid extensions, resulting in AHPIP and MHPIP N-termini, respectively.

c C18 diacid gGlu-OEG-OEG-C2DA-Ac side chain. pEC_50_: negative logarithm of EC_50_.

### Fatty-Diacid Modification in the FGF21 C-Terminal without Loss of Biological Activity

Our strategy to use fatty-diacid modification in the FGF21 C-terminal region aimed to mitigate FAP and carboxypeptidase-like degradation and provide *t*_1/2_ extension via reversible albumin binding. This would have to be achieved without compromising the biological activity of the free (not albumin-bound) fraction of the FGF21 analog. Results from our modification site scan in the C-terminal region of the **4** backbone are summarized in Table 2 and Supporting Information C [Table S1]. Cysteamine protection (of the mutant cysteine residue intended for fatty-diacid side chain conjugation) at positions 167, 170, 171, 172, or 180 had only a marginal effect on the potency of FGF21. Conversely, cysteamine protection at positions 176, 178, 179, or 181 decreased potency by at least 10-fold. Fatty-diacid modification in position 181 (**18**) markedly decreased potency compared with that of the stabilized backbone (**4**) (39 vs 2.0 nM Table 2). In contrast, compound **15**, which carries the fatty-diacid side chain in position 180, was of similar potency as the stabilized backbone (**4**) (3.3 vs 2.0 nM, Table 2). Potency loss following modification in position 181 (**18**) as well as preservation of potency with modification in position 180 (**15**) was corroborated in the AlphaScreen binding assay with **18** binding significantly weaker than the stabilized backbone (**4**) (pIC_50_ 5.72 ± 0.02 for **18** vs 6.18 ± 0.10 for **4**, *p* = 0.01, Figure 3 and Supporting Information C [Table S2]) but **15** displaying binding like that of **4** (pIC_50_ 6.13 ± 0.80 vs 6.18 ± 0.10, Figure 3 and Supporting Information C [Table S2]). From these data, we inferred that the incorporation of a fatty-diacid side chain in position 180C was well tolerated and did not further lower the potency of the stabilized backbone. This clearly differentiates **15** from other FGF21 analogs carrying a *t*_1/2_ extending moiety in the outermost C-terminal sequence. Thus, both linear 20 kDa PEGylation on FGF21 Tyr179^62^ and antibody-conjugation to an FGF21 Ser181Lys mutant residue (CovX-body molecular format)^23^ resulted in >100-fold decreased potency in vitro, while recombinant Fc-elongation of the FGF21 C-terminal also led to dramatically reduced KLB binding and loss of in vitro activity.^33^

While the absence of Ser181 from **15** (**17**) slightly lowered FGFR1c/KLB binding (pIC_50_ 5.79 ± 0.05 vs 6.13 ± 0.08, *p* = 0.04, Figure 3 and Supporting Information C [Table S2]) this did not translate to potency loss in the functional assay (Table 2). These results agree with previously reported minor but assay-dependent effects of removing Ser181 from FGF21^10^ and supported our decision to keep Ser181 in **15**.

**Figure 4:**
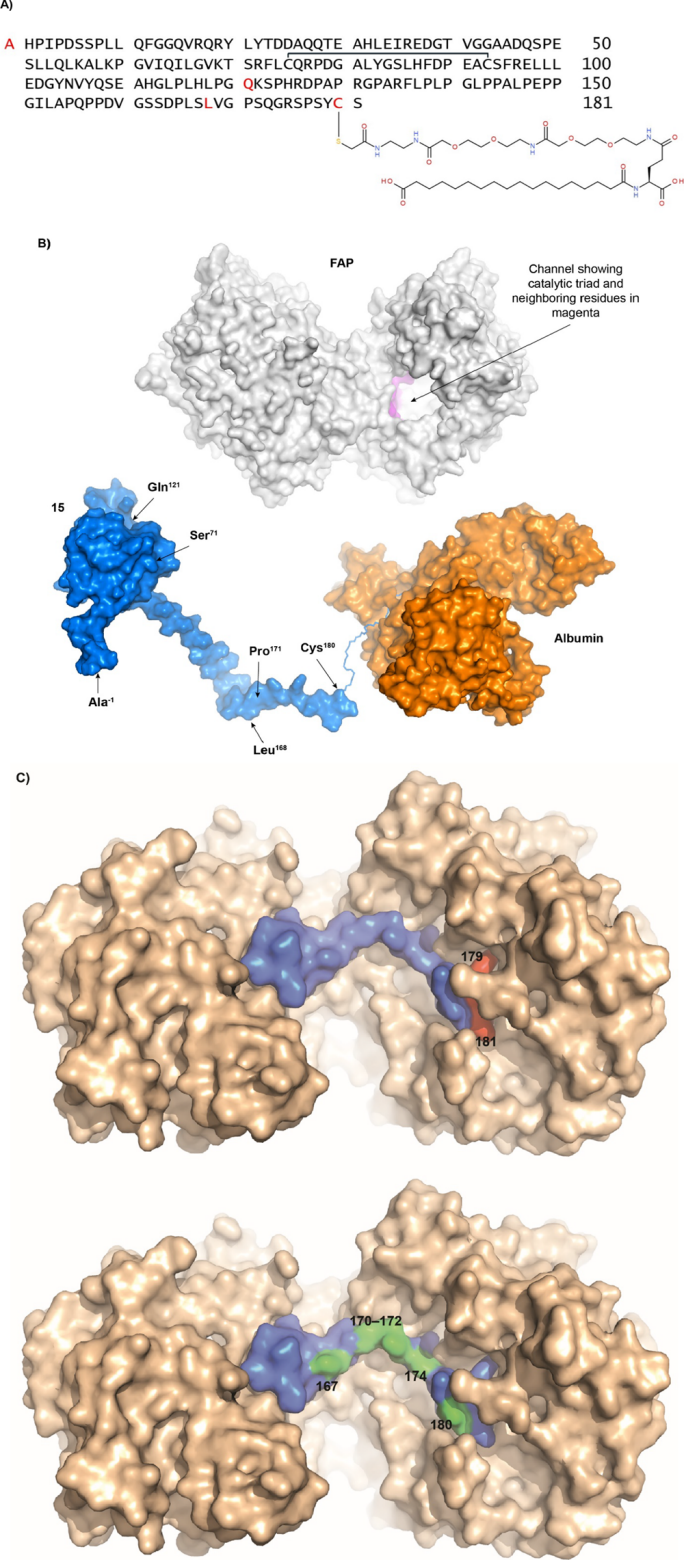
Structural model of the zalfermin (**15**) FGF21 analog and illustration of interactions with albumin and KLB. Numbering of amino acids corresponds to human FGF21. (A) Amino acid sequence with rendition of the fatty-diacid side chain. The naturally occurring disulfide bridge between Cys75 and Cys93 is indicated by a line. Red font represents a mutation relative to human FGF21. The N-terminal is elongated by an alanine residue (−1Ala) and in-sequence mutations include Asn121Gln, Met168Leu, and Ala180Cys. A C18 diacid gGlu-OEG-OEG-C2DA-Ac side chain is attached at position 180. (B) Representative AlphaFold model of **15** shown in blue and potential interaction with albumin (orange) based on albumin−somapacitan cocrystal structure (PDB entry 6QIO). The FAP enzyme is colored gray, with the catalytic site and surrounding residues in magenta. FGF21 positions mutated in **15** as well as position 71 (modification site in compound **5**) and 171 (part of the FAP cleavage Pro171/Ser172 site) are indicated. (C) Crystal structure of FGF21 C-terminal region residues (158-Ser181, in colors) in complex with binding to KLB (in gold) (PDB entry 5VAQ).^63^ The structure predicts introduction of a fatty-diacid side chain on some (red color) but not other (green color) FGF21 amino acid residues to cause steric clash with KLB. For clarity, some FGF21 residues (168, 169, 171, 173, 175–178) are not annotated and red/green color-coded since they are largely hidden from the chosen point of view. FAP: fibroblast activation protein; KLB: beta-klotho. Figure 4B,C were prepared using PyMOL.^64^

These findings provided options to pursue modification in position 180 or more distant from the extreme C-terminal, as is the case in pegozafermin which carries a 20 kDa glycoPEGylation on position 172.^42^ While a fatty-diacid side chain in position 180 eliminates the risk of potency loss due to carboxypeptidase-like cleavage from the C-terminal, placement of the modification site too far from the C-terminal will reduce or annul this protection. Structural modeling predicts albumin binding to a fatty-diacid side chain on position 180 to eliminate the possibility for FAP cleavage after Pro171 (Figure 4). These considerations led us to focus on position 180 as the preferred modification site.

### A C18 Diacid Side Chain at Position 180C Improves PK and PD in Mouse

The length of the fatty-diacid side chain positively correlates with albumin-binding affinity.^50^ Considering that covalent attachment of Fc, antibody, or PEG in the FGF21 C-terminal severely reduces activity,^23,33,62^ we expected potency loss during noncovalent albumin binding. To identify a fatty-diacid side chain with albumin affinity supporting both *t*_1/2_ extension and biological activity, we investigated how the side chain length affects in vitro potency and PK and PD properties (Table 3).

**Table 3:** Impact of Fatty-Diacid Side Chain Length and Albumin Concentration on In Vitro Potency, Systemic *t*_1/2_, and Body-Weight-Lowering Effects^a^

compound	mutations^b^	modification on 180C	potency (EC_50_, nM)				*t*_1/2_ (h)	body-weight loss (g)	
			0% HSA	0.1% HSA	1.0% HSA	1.5% HSA	mice (iv)	lean mice	DIO mice
**1**	–1M		NA	NA	NA	NA	0.7	0.5^d^	NA
**4**	–1A, 121Q, 168L		2.0	1.4	0.9	1.5	1.1	NA	NA
**15**	–1A, 121Q, 168L, 180C	C18 fatty diacid^c^	3.3	23	195	198	12.3	2.6^e^	9.8^f^
**16**	–1A, 121Q, 168L, 180C	cysteamine	2.5	1.8	2.1	NA	NA	NA	NA
**20**	–1A, 121Q, 168L, 180C	C12 fatty diacid^c^	5.8	3.7	5.2	NA	1.1	0.6^d^	NA
**21**	–1A, 121Q, 168L, 180C	C14 fatty diacid^c^	5.2	4.0	5.8	8.6	0.9	0.9^d^	NA
**22**	–1A, 121Q, 168L, 180C	C16 fatty diacid^c^	3.5	4.2	52	92	3.2	1.1^e^	NA
**23**	–1A, 121Q, 168L, 180C	C20 fatty diacid^c^	3.8	47	NA	173	23.6	NA	7.0^f^

a In vitro potency in the presence of 0, 0.1, 1, or 1.5% HSA was measured as phosphorylation of ERK in HEK293 cells transfected with human KLB. Body-weight loss was measured after 6 days (lean mice) or 10 days (DIO mice) dosing of compound.

b –1A and −1M are single amino acid extensions resulting in AHPIP and MHPIP N-termini, respectively.

c C12, C14, C16, C18, or C20 diacid gGlu-OEG-OEG-C2DA-Ac side chain, as indicated.

d 1 mg of compound/kg of body weight, twice-daily sc dosing.

e 1 mg compound/kg body weight, once-daily sc dosing.

f 0.3 mg compound/kg body weight, once-daily sc dosing. DIO: diet-induced obesity; KLB: beta-klotho; NA: not applicable. For statistical strength of data, see Supporting Information D (Tables S3 and S4 and Figure S3).

In the absence of HSA, the potency of compounds carrying a C12–C20 side chain at position 180 (**15**, **20**, **21**, **22,** and **23**) was similar to the stabilized backbone (**4**). An HSA concentration-dependent reduction of in vitro potency was observed for analogs carrying a C16, C18, or C20 side chain (Table 3) but not for analogs carrying a shorter (C12 or C14) or no side chain, suggesting that only the longer side chains bound albumin to a degree that impacted FGF21 receptor activation. These data imply that **15**, when albumin-bound, is restrained from activating its receptor, probably through a steric effect preventing binding to KLB (Table 3, Figure 4C and Supporting Information D [Figure S3]).

In line with these findings, longer side chains were required to extend *t*_1/2_ in vivo. Thus, C16, C18, and C20 diacid side chain analogs showed increased systemic *t*_1/2_ of 3.2, 12.6, and 23.6 h in iv-dosed lean mice (Table 3).

Body-weight loss in lean mice increased with the side chain length (C12–C18) of the FGF21 analog so that the greatest effect at 1 mg/kg once daily (2.6 g after 6 days of treatment) was achieved with **15** containing the C18 side chain. A head-to-head comparison showed the superiority of the C18 (**15**) compared with the C20 (**23**) side chain in diet-induced obese (DIO) mice. After 10 days of treatment (0.3 mg/kg once daily), **15** and **23** reduced body weight by 9.8 and 7.0 g (*p* < 0.001), respectively (Table 3 and Supporting Information E [Figures S5 and S6]). Furthermore, **15** reduced the body weight of DIO mice in a dose-dependent manner (Supporting Information E).

Taken together, the superior PD effect with the C18 fatty-diacid side chain (**15**) is ensured via the optimal balancing of *t*_1/2_ extension caused by albumin binding and free **15** and thus fully active FGF21 analog fraction. The shorter fatty-diacid side chains provide suboptimal *t*_1/2_ extension while the C20 side chain binds albumin too tightly and thus hinders optimal biological function. The concept of using reversible albumin binding to secure long circulating *t*_1/2_ (via the inactivated bound fraction) as well as high in vivo efficacy (via the free active fraction) is well proven.^50,65^

### Albumin Binding Proteolytically Stabilizes toward FAP Cleavage of **15**

**Figure 5:**
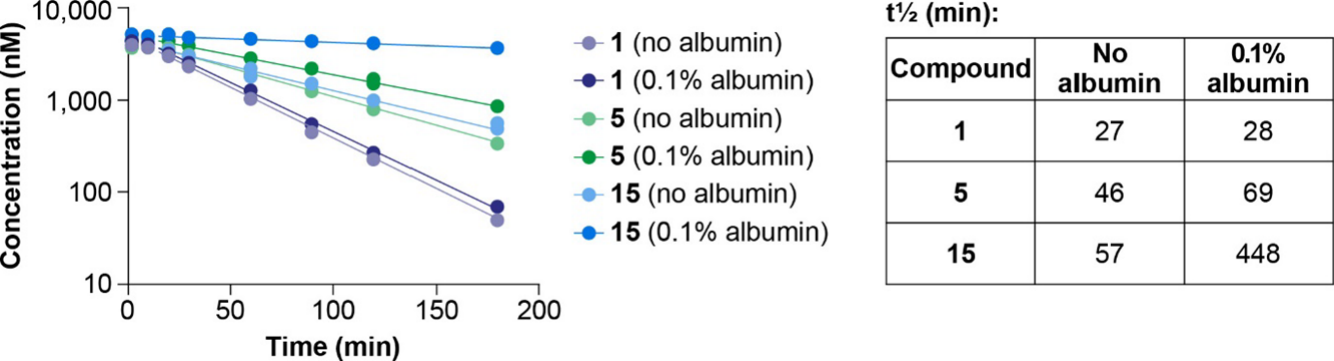
In vitro proteolytic stability of **1**, **5**, and **15** after incubation with fibroblast activation protein. The effect of albumin binding was evaluated by the addition of 0.1% albumin.

Endogenous human FGF21 is cleaved after Pro171 by FAP.^19,20^ Given the proximity of the C18 fatty diacid of **15** to the natural FAP cleavage site, we investigated the susceptibility of **15** to FAP cleavage in vitro (Figure 5). Met-FGF21 (**1**) was included to determine FAP cleavage in the absence of a fatty-diacid side chain. Compound **5** was used as a comparator since it differs from **15** by having the side chain in position 71, which is distant from the position of the side chain in **15** (see Figure 4B). In the absence of albumin, the *t*_1/2_ values of **1**, **15**, and **5** were comparable (27–57 min). It was confirmed by LC–MS that the decline in concentration for each compound was due to proteolytic cleavage between Pro171 and Ser172. The presence of albumin markedly increased stability of **15** toward FAP degradation (to 448 min), whereas albumin only had marginal or no effect on **1** and **5***t*_1/2_. Thus, the protection of **15** against FAP cleavage in the presence of albumin can be ascribed to the positioning of the side chain in position 180. The steric hindrance at site 180 modification, but not at site 71 modification, agrees with our structural model of **15** and its interaction with albumin (Figure 4B). This suggests that when **15** is bound to albumin the active site of FAP cannot access the cleavage recognition sequence in the **15** backbone.

### Model of **15** and Structural Aspects Related to Albumin Binding, FAP Degradation, and Modification Position

The amino acid sequence and the fatty-diacid side chain of the FGF21 analog **15** (20.3 kDa) are shown in Figure 4A. A structural model of **15** (without fatty-diacid side chain) was generated in AlphaFold,^66^ indicating multiple extended conformations of the FGF21 C-terminus in agreement with this region being highly flexible. A representative AlphaFold model of **15** is shown in Figure 4B. The crystal structure of albumin in complex with somapacitan (Protein Data Bank [PDB] entry 6QIO) was used to illustrate the distance and size relationship between **15** and albumin in the albumin-bound state of **15**, adjusting the linker length to match that of **15**.^67^**15** may bind other sites on albumin as well.^50^Figure 4B also shows the FAP enzyme (PDB entry 1Z68) with the indication of key amino acid residues in the catalytic tunnel.^68^ The three molecules are drawn to scale. Albumin binding to the fatty-diacid side chain in position 180 will sterically hinder FAP from accessing the Pro171/Ser172 cleavage site which is present in endogenous FGF21^19,20^ and will prevent KLB binding resulting in loss of potency (Table 3).

The structure–activity relationship for FGF21 C-terminal region analogs (Table 2) is in agreement with the interactions between FGF21 and KLB (Figure 4C). Thus, analogs with retained potency are predicted to be structurally permissible (engineered in positions 167, 168, 170–172, and 180), while analogs showing loss of potency (positions 176–179 and 181) are structurally unfavorable.

Taken together, the structural considerations support **15** having retained activity and albumin-mediated protection against C-terminal degradation.

### MHC Class II Binding Prediction Analysis of **15**

Given the immunogenicity risk of cross-reactivity with endogenous FGF21, the reduction of immunogenicity and antidrug antibody (ADA) development is important for FGF21 analogs. To address the key process of peptides binding to human leukocyte antigen (HLA) class II, which is a necessary step in the development of ADAs, an in silico prediction analysis was performed, as described previously.^69^ The amino acid sequence of **15** was analyzed for potential T-cell neo-epitopes using human FGF21 as the reference sequence. No neo-epitopes were predicted, indicative of a low immunogenicity potential. In addition, modification with fatty diacids has the potential to reduce the immunogenicity risk as shown for other peptides.^70^ Other FGF21 analogs have been found to induce low titers of non-neutralizing ADAs in the clinical setting^60,71^ and further clinical characterization of **15** is required to understand the immunogenicity risk.

### FGF-Receptor Selectivity of **15**

The FGF-receptor selectivity of compound **15** was compared to Met-FGF21 (**1**) in Ba/F3 cells overexpressing KLB in addition to FGFR1c, R2c, R3c, or R4 (Table 4). As seen in Table 4 and Figure S4, **15** and Met-FGF21 (**1**) were equally potent in activating FGFR1c/KLB and FGFR3c/KLB receptor complexes. Met-FGF21 (**1**) and **15** did not activate the FGFR2c/KLB or the FGFR4/KLB complex. The results for FGFR4/KLB agree with previous data.^32,72−74^ There are contrasting observations of whether FGF21 activates FGFR2c/KLB. FGFR2c/KLB activation is observed in transfected L6 cells,^32,73,74^ while activation was not observed by Suzuki et al.^8^ using a Ba/F3 cell system like ours. It is possible that the discrepancy regarding FGFR2c/KLB engagement between groups relates to the cellular background of the assays (L6 vs Ba/F3) employed. However, in our AlphaScreen binding assay, we were unable to establish a binding signal between FGF21, FGFR2c, and KLB, supporting the idea that FGF21 has negligible engagement of FGFR2.

**Table 4:** FGF-Receptor Selectivity of Compound **15** in Comparison to Met-FGF21 (**1**), Met-FGF1 (**24**), and Met-FGF19 (**25**)^a^

compound	potency EC_50_ (nM), 0% HSA [95% CI]			
	FGFR1c	FGFR2c	FGFR3c	FGFR4
**1**	3.7 [2.4; 5.8]	inactive	10.8 [5.5; 20.9]	inactive
**15**	4.1 [3.2; 5.3]	inactive	11.9 [11.0; 12.9]	inactive
**24**	NA	0.6 [0.5; 0.8]	NA	NA
**25**	NA	NA	NA	2.3 [2.0; 2.6]

a Determined in Ba/F3 cells overexpressing KLB in addition to FGFR1c, R2c, R3c, or R4. Four independent experiments were performed for each determination. CI: confidence interval; Met-FGF21: human FGF21 with an N-terminal Met extension; NA, not applicable.

**Figure 6:**
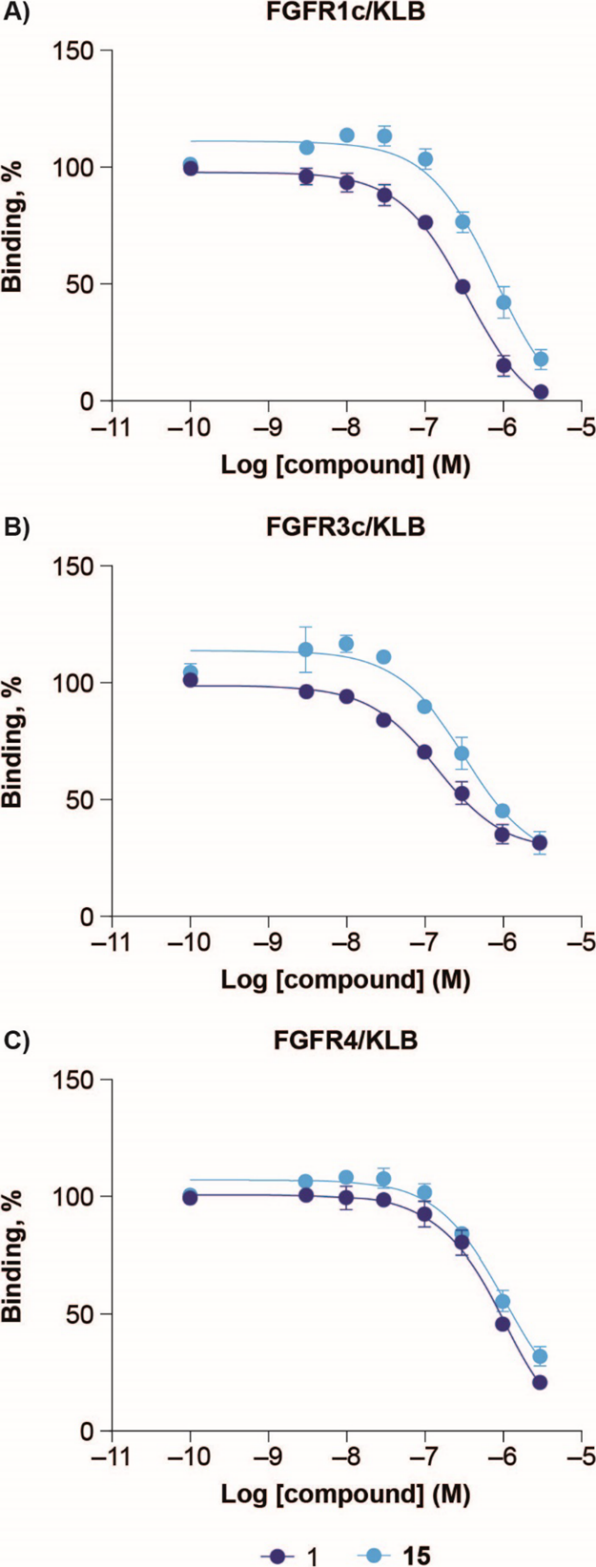
AlphaScreen binding assay data for **1** and **15** toward FGFR1c/KLB (A), FGFR3c/KLB (B), and FGFR4/KLB (C). Data are mean ± SEM (*n* = 3).

The binding of **15** and **1** across FGF receptor subtypes shows that the receptor selectivity of **15** follows that of **1** across FGFR1c, FGFR3c, and FGFR4 in complex with KLB (Figure 6). It is important to note that the binding to FGFR4/KLB is not accompanied by activation of the receptor (Table 4, Figures 6 and S4) in agreement with previous publications.^7,73^

### Species Qualification of **15**

Human FGF21 has previously been shown to bind^32,55^ and activate the mouse FGF21-receptor complex with higher potency than the human FGF21-receptor complex. To test whether protein engineering of **15** altered the potency across species, which could ultimately impact the therapeutic efficacy, the potency in mice, cynomolgus monkeys, and humans was compared in vitro.

In HEK293 cells overexpressing the cynomolgus monkey or human KLB, **15** and Met-FGF21 were on par (Table 5), while for mouse KLB, **15** was slightly more potent than Met-FGF21. Both **15** and Met-FGF21 activate phosphorylation of ERK signaling with approximately 10-fold increased potency in cells overexpressing the mouse KLB compared with human KLB, while both compounds are slightly more potent in the cells overexpressing cynomolgus KLB compared with the human KLB-expressing cells. While potency differences across cell lines should be cautiously interpreted, the higher potency of human FGF21 toward the mouse KLB agrees with previous studies.^32,55^ The potency of **15** and Met-FGF21 was also comparable in HEK293/KLB cells (Table 4) while human FGFR1c/KLB binding data showed a small but significant increase in the IC_50_ of **15** compared with **1** (Figures 3 and 6, and Table S2). Taken together, we conclude that **15** and Met-FGF21 have comparable in vitro potencies across the species.

**Table 5:** In Vitro Potency of Compound **15** and Met-FGF21 (**1**) in HEK293 Cells Overexpressing Mouse, Cynomolgus Monkey, or Human KLB^a^

compound	mouse KLB mean EC_50_ (nM) [95% CI]	cynomolgus monkey KLB mean EC_50_ (nM) [95% CI]	human KLB mean EC_50_ (nM) [95% CI]
**1**	0.3 [0.2; 0.4]	1.8 [1.1; 2.8]	3.0 [2.2; 4.3]
**15**	0.1 [0.1; 0.2]	1.4 [0.9; 2.2]	2.8 [2.3; 3.4]

a Six independent experiments were performed for each determination. CI: confidence interval; KLB: beta-klotho; Met-FGF21: human FGF21 with an N-terminal Met extension.

### Extended Systemic *t*_1/2_ of **15** across Species

The extended systemic *t*_1/2_ in mice (Table 3) was confirmed for **15** in minipigs, LYD pigs, and cynomolgus monkeys (Table 6). In cynomolgus monkeys, **15** showed an iv *t*_1/2_ of 53 h as compared to 2 h for Met-FGF21,^2^ supporting once-weekly dosing in humans. *t*_1/2_ in cynomolgus monkeys has been reported for two other current clinical candidates, thus sc-dosed pegozafermin and iv-dosed efruxifermin show *t*_1/2_ of approximately 50 h^36^ and 76 h,^32^ respectively.

**Table 6:** Summary of Key PK Parameters for **15** Dosed IV to Mouse, Minipig (*n* = 3), LYD Pig (*n* = 3), and Cynomolgus Monkey (*n* = 2)^a^

parameter	mouse^b^	minipig	LYD pig	cynomolgus monkey
AUC/D (h·kg/L)	NA	932 ± 39	759 ± 23	872 ± 84
CL (L/kg/h)	0.005144	0.001077 ± 0.00004	0.00132 ± 0.00004	0.00116 ± 0.00011
Vd (L/kg)	0.092	0.109 ± 0.003	0.091 ± 0.004	0.088 ± 0.009
*t*_1/2_ (h)	12	70 ± 4	48 ± 3	53 ± 0.5

a Data are mean ± SEM.

b AUC/D and SEM were not calculated due to sparse sampling in mouse PK. AUC: area under curve; CL: clearance; D: dose; NA: not applicable; Vd: volume of distribution.

The **15** FGF21 analog shows a low volume of distribution (Vd) across species, indicating that **15** is primarily present in plasma. Furthermore, an acceptable and clinically relevant absolute sc bioavailability of **15** was observed in LYD pigs (54%) and cynomolgus monkeys (92%). The PK characterization for FGF21 **15** is, overall, in line with that for other C18 fatty diacid *t*_1/2_-extended analogs, including the once-weekly semaglutide peptide which, in minipigs, shows a *t*_1/2_ of 46 h and a Vd of 0.1019 L/kg.^65^ In mice, FGF21 crosses the blood−brain−barrier^75^ and its action in the mouse brain has been reported to be important for the metabolic effects of FGF21.^76^ While the determinants, regulation, and impact of FGF21 agonism in the brain for pharmacological read-outs remains to be understood,^77^ the different *t*_1/2_ extension strategies (fatty acid, PEGylation, Fc-fusion) used in current clinical candidates may prove to differentiate their access to and action in the brain.

### Metabolism of **15** in Mouse and Minipig

The metabolism of **15** in mouse and minipig was investigated by LC–MS analysis of the PK study plasma samples. Analog **5** was used to determine the degradation of the nonengineered FGF21 C-terminal region in mouse; in addition, the metabolism of Met-FGF21 (**1**) was studied in mouse and minipig (see Supporting Information F [Table S5, Figure S7] and A [Figure S1]).

**Figure 7:**
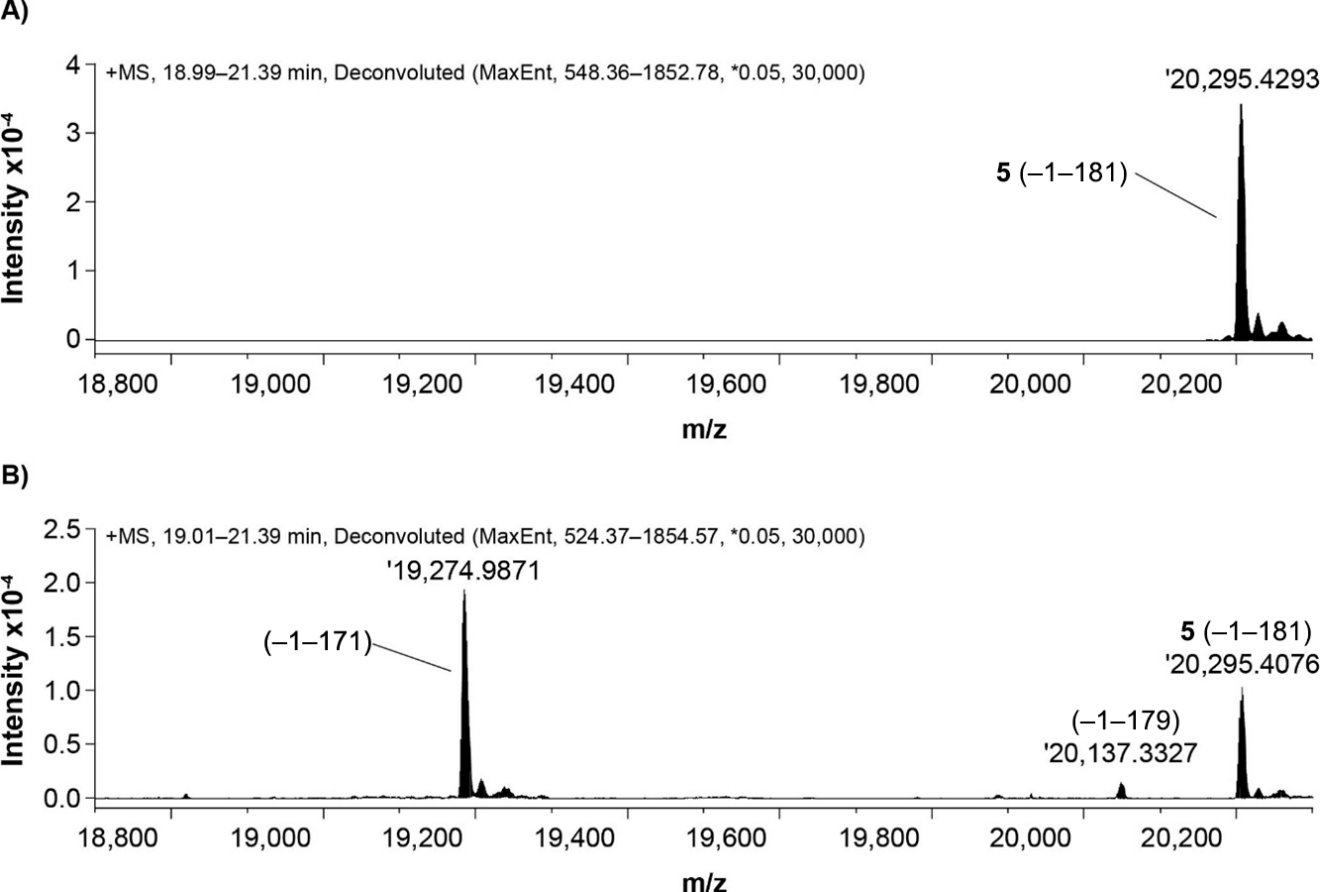
Identification of metabolites in plasma from mice dosed with **5** (analog with nonengineered FGF21 C-terminal). Deconvoluted MS spectra from LC–MS analysis are shown at 5 min (A) and 12 h (B) after dosing. Theoretical and experimentally determined masses deviate by less than ±2 ppm.

In mouse plasma samples, intact mass analysis by LC–MS combined with MS/MS sequencing enabled the identification of the dominant −1–171 metabolite (FAP cleavage product) of **5** (Figure 7 and Supporting Information G [Table S6 and Figure S8]). In addition, multiple less-abundant metabolites of **5** were also identified, including −1–151, −1–167, and three probable carboxypeptidase-like products: −1–180, −1–179, and −1–178. Some of these metabolites are within the retention time window shown in Figure 7.

**Figure 8:**
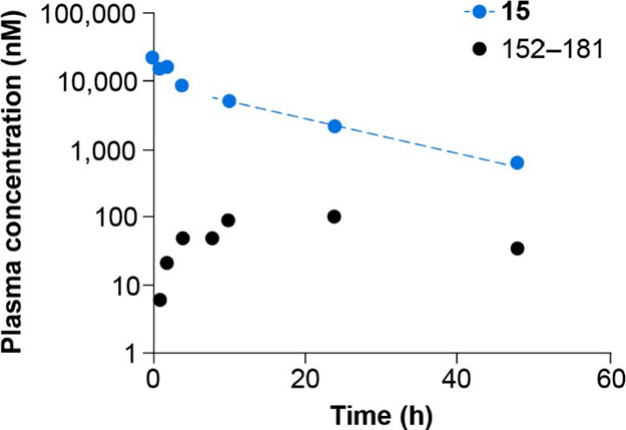
Exposure of **15** and its 152–181 metabolite in mice (20 mg/kg, iv). Quantification was conducted by LC–MS.

A 4-fold longer *t*_1/2_ in mice (iv) was obtained for **15** (12 h, Table 6) than for **5** (3 h). This difference was driven by proteolytic stabilization of **15** since the level of metabolites was much lower for **15** than for **5** (see Figure 7 for the FAP metabolite). The most abundant metabolite from **15** was 152–181 (exposure ∼2% of that of **15** based on AUC, see Figure 8) resulting from cleavage between residues Gly151 and Ile152. Furthermore, the −1–180 and 172–181 metabolites were identified at lower levels. The metabolic profile of **15** in minipig was similar to that in mice (Supporting Information F [Table S5 and Figure S7]).

In minipig and mouse PK (iv) plasma samples of Met-FGF21 (**1**), we observed C-terminal carboxypeptidase-like cleavage and FAP cleavage (see Supporting Information F [Table S5 and Figure S7]), resembling the data for **5** in the mouse.

Our approach to addressing C-terminal metabolism differs from that used in the Fc-FGF21(RGE) analog,^32^ which contains the two C-terminal point mutations Pro171Gly and Ala180Glu. That compound, later named efruxifermin, also showed increased *t*_1/2_ in mice, cynomolgus monkeys, and humans.^34^

In summary, the high metabolic stability of **15** can be ascribed to N-terminal Ala-1 elongation protecting against DPP-IV cleavage and C18 fatty-diacid side chain in position 180 protecting against FAP cleavage and carboxypeptidase-like activity.

### Biophysical and Formulation Properties of **15** Support a Liquid Formulation

The biophysical properties of **15** were investigated to determine whether a liquid formulation for sc injection is possible.

Considering that **15** has an acidic isoelectric point, phosphate buffer was chosen for slightly alkaline and largely temperature-independent pH control. Our structural model of **15** suggests the existence of patches of contiguous hydrophobicity. To provide an amphiphilic interface between hydrophobic surfaces and polar solvent, glycerol was chosen as a tonicity agent.^78^

**Figure 9:**
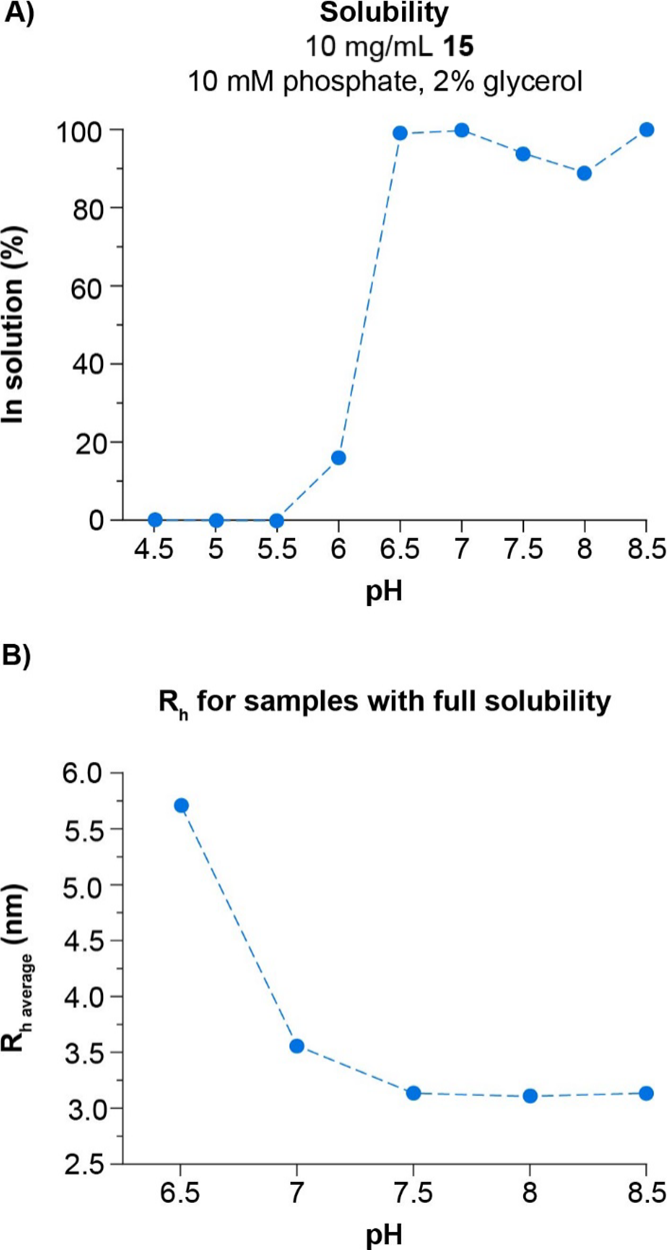
Solubility (A) and *R*_h_ (B) of **15** as a function of pH. *R*_h_ determination was done on supernatants obtained from centrifugation of samples used for solubility determination. *R*_h_: hydrodynamic radius.

The solubility of **15** was found to be very limited below pH 6 while fully soluble at pH ≥ 6.5 (Figure 9). The solubility profile is regulated by pH-dependent aggregation prior to the observed zone of precipitation. This was shown by dynamic light scattering (DLS) determination of the average **15** hydrodynamic radius (*R*_h_). While the *R*_h_ was 3.1 nm at pH ≥ 7.5, a significant increase in *R*_h_ was seen at pH 7.0, which was even more pronounced at pH 6.5.

**Figure 10:**
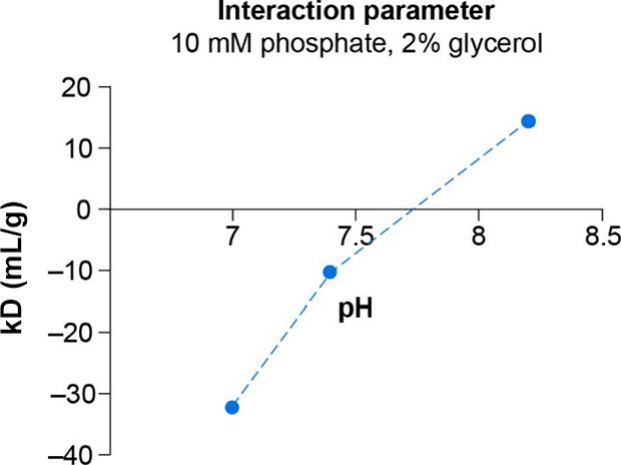
Interaction parameter kD at various pH. kD: interaction parameter for dynamic light scattering.

The effect of the pH on colloidal stability was investigated. The diffusion interaction parameter kD was determined using DLS^79,80^ (Figure 10). The kD was negative (−32 g/mL) at pH 7.0 and positive at pH 8.2. This indicates repulsive forces between the molecules to be dominating at pH 8.2, hence enhanced colloidal stability is expected.

**Figure 11:**
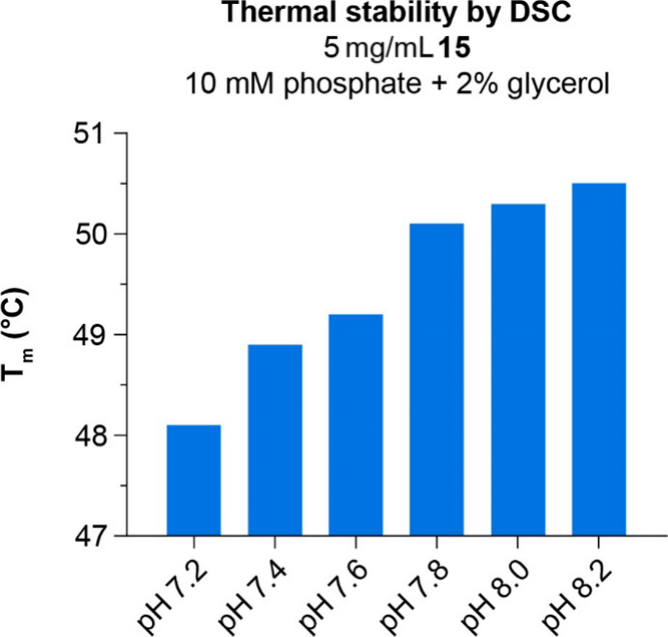
Temperature of midpoint of thermal transition (*T*_m_) for **15** at various pH. DSC: differential scanning calorimetry.

Thermal stability was determined by differential scanning calorimetry (DSC). As seen for the colloidal stability, the temperature of the midpoint of thermal transition (*T*_m_) increases with increasing pH, and the highest *T*_m_ (50.5 °C) is found at pH 8.2 (Figure 11). Furthermore, *T*_m_ decreased with increasing protein concentration (not shown). These results align well with studies of FGF21 which showed a *T*_m_ of 50 °C (DSC) at ∼1.5 mg/mL^21^ and 62 °C circular dichroism (CD) at 0.2 mg/mL.^81^ In LY2405319, thermal stability was enhanced by the introduction of an extra disulfide bridge.^21^ Thermal unfolding of FGF21 has recently been shown to be fully reversible.^81^ Taken together, we find the thermal stability of **15** to be appropriate for pharmaceutical uses.

All in all, pH is a major regulator of the biophysical properties of **15**. Solubility, colloidal stability, and thermal stability are all increasing with increasing pH and pH 8.2 is most suitable for the liquid formulation.

**Figure 12:**
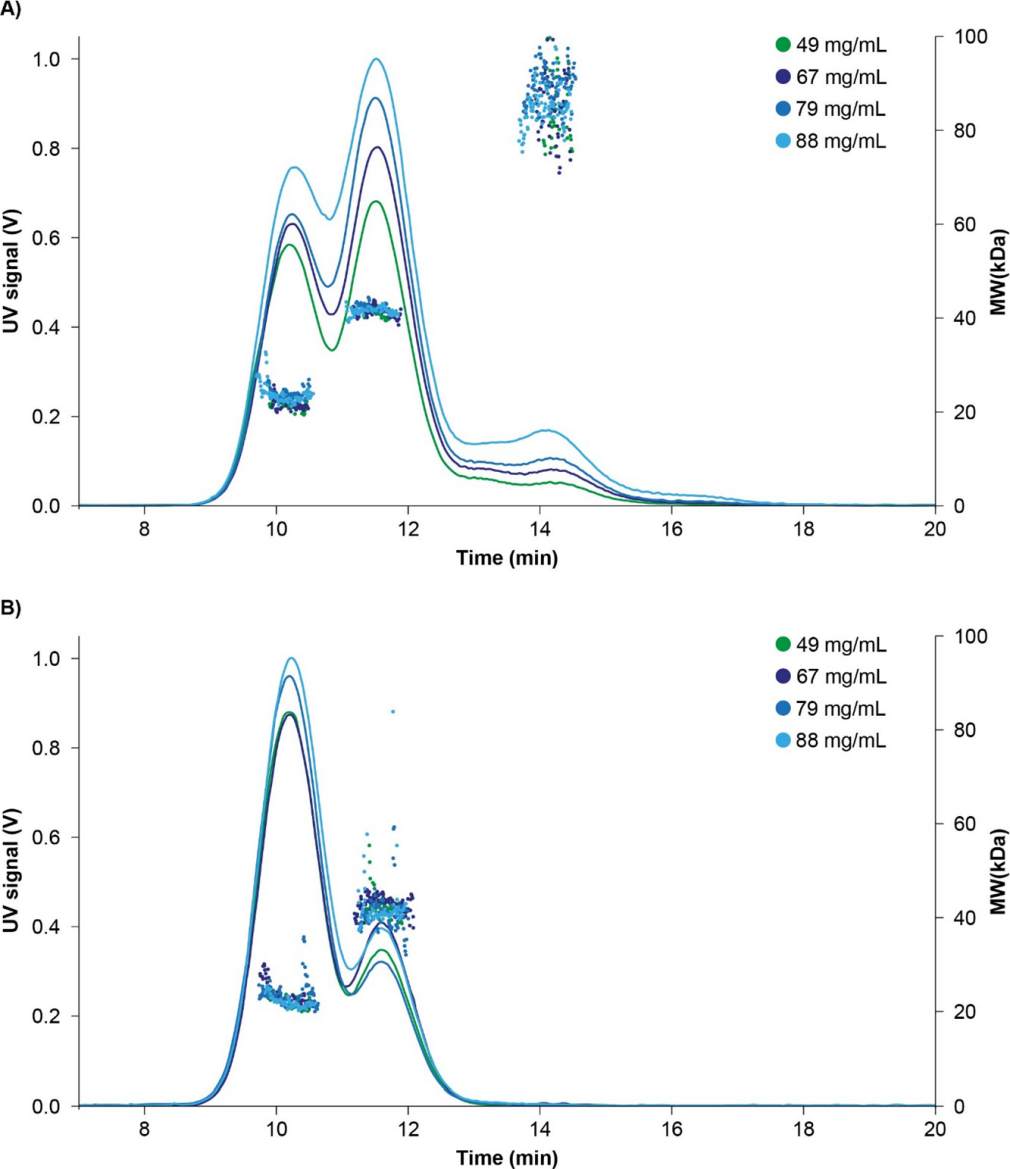
Fractogram from AF4-MALS for **15** in concentrations of 49–88 mg/mL (A) and after dilution to 5 mg/mL (B). 1 μL of the original and 10 μL of diluted samples were injected, and 10 mM phosphate pH 8.2 with 10 mM NaCl was used as running buffer. The determined molar masses are indicated over the various peaks. AF4-MALS: Asymmetrical Flow-Field Flow Fractionation Multi-Angle-Light-Scattering.

The concentration-dependent self-association (noncovalent agglomeration such as dimer or larger oligomers) of **15** is pronounced, as is also the case for FGF21.^33^ Samples with **15** concentrations ranging from 49 to 88 mg/mL in 10 mM phosphate, pH 8.2 with 2% glycerol were injected to an Asymmetrical Flow-Field Flow Fractionation Multi-Angle-Light-Scattering (AF4-MALS) system. The level of dimers and larger oligomers increases with **15** concentration, as shown in AF4-MALS fractograms (Figure 12). After dilution to 5 mg/mL (using 10 mM phosphate, pH 8.2, 2% glycerol buffer), the samples were analyzed by AF4-MALS again, and self-association was clearly shown to be reversible.

Accelerated stability read-outs for quiescent storage of **15** (15 mg/mL) are shown in Table 7. The concentration was chosen to support the planned phase 1 clinical studies. Very little degradation is observed at 4 °C, suggesting that long-term refrigerated storage is feasible. At 30 °C, low-level degradation is observed with the L-isoaspartate102 form of **15** constituting the major degradation product (0.9% formation per month), supporting in-use storage at ambient temperature.

**Table 7:** Chemical Stability of **15** Formulated at 15 mg/mL in 10 mM Phosphate Buffer, pH 8.2 with 2% Glycerol^a^

storage temperature (°C)	purity loss (%/month)	IsoAsp102 formation (%/month)	HMWP formation (%/month)
4	0.02	<0.01	0.01
30	2.2	0.9	0.1

a Quiescent storage followed by reverse-phase ultra-performance liquid chromatography and size-exclusion high-performance liquid chromatography analysis. Data at 30 °C were collected in real time, whereas 4 °C data were estimated from Arrhenius calculations of data from 25, 30, and 37 °C. HMWP, high molecular weight protein.

## Conclusions

Here, we describe the development of the long-acting FGF21 analog zalfermin (**15**) intended for once-weekly sc dosing. The fatty-diacid modification in the C-terminal region achieved multiple important goals, including: (i) *t*_1/2_ extension via albumin binding; (ii) protection toward FAP-mediated inactivating C-terminal degradation; and (iii) potent FGF-receptor complex activation. These features of zalfermin contributed to a high in vivo biological efficacy. We engineered the C-terminal region of zalfermin by screening side chain modification sites and fatty-diacid side chains. The exact positioning of the fatty-diacid side chain within the C-terminal region, as well as the strength of albumin binding by the side chain, posed a particular design challenge. Thus, careful selection of the modification site was needed to avoid dramatic loss of potency resulting from disrupted KLB binding. Too strong albumin binding limited biological efficacy since it brought a too high proportion of FGF21 analog to the albumin-bound state which does not allow KLB binding. The C-terminal design was therefore a balancing act which was ultimately solved by attaching a C18 diacid fatty-diacid side chain to a cysteine in position 180. The activity and protection toward FAP cleavage of these compounds is in good agreement with structural modeling of the interactions between the FGF21 analogs, the FGF receptor, KLB, albumin, and FAP. In addition, zalfermin contains an N-terminal alanine residue extension which offers protection against DPP-IV degradation, an Asn121Gln mutation to prevent deamidation, and a Met168Leu mutation to prevent oxidation. Zalfermin has FGF-receptor selectivity resembling that of human FGF21 and is predicted to be low-immunogenic. Translation of receptor complex activation was shown across mice, cynomolgus monkeys, and human species. A liquid formulation of zalfermin supports long-term refrigerated storage, one-month in-use storage at ambient temperature, and sc injection. Phase 1 clinical (NCT03015207, NCT04722653, NCT03479892) studies of safety, PK, and efficacy have supported the further progression of zalfermin which is currently in phase 2b for the treatment of MASH (NCT05016882).

## Experimental Section

### Production of FGF21 (0), Met-FGF21 (1), and FGF21 Analog Backbones

The DNA and amino acid sequences for human FGF21 are available from public databases with accession numbers EMBL:AB021975 and UNIPROT:Q9NSA1, respectively. DNA sequences encoding FGF21 amino acid sequences were inserted into a modified vector (pET11c-based) under the control of a T7 promoter and were transformed into a BL21 (DE3)-derived host strain. Isopropylthio-β-galactoside (IPTG)-induced FGF21 protein expression resulted in *E. coli* inclusion bodies, which were recovered. Protein resolubilization was done in 50 mM Tris pH 8.0, including also 2 M urea and 5 mM cysteamine in the case of FGF21 analogs containing an introduced cysteine residue. The protein was captured by anion exchange Q Sepharose Fast Flow chromatography, purified by Phenyl FF hydrophobic interaction chromatography, and where needed further purified by Source 30Q anion exchange chromatography. The production of FGF21 without N-terminal elongation (**0**) involved the expression of a precursor, from which an extension (−6SKTKTK) was cleaved off using a dipeptidyl peptidase I (DAP1) enzyme, which was recombinantly expressed and purified at Novo Nordisk. For cysteamine-protected backbones, the pure fractions were pooled and concentrated. Analogs not containing a cysteamine-protected cysteine mutant residue were furthermore buffer-exchanged by ultrafiltration/diafiltration (UF/DF) to a vehicle of 10 mM phosphate, 2% glycerol, pH 8.15. Following 0.22 μm sterile filtration, all compounds were stored frozen.

### Production of Met-FGF1 and Met-FGF19

Production methods for Met-FGF1 (**24**) and Met-FGF19 (**25**) were similar to those used for Met-FGF21. **24** is human FGF1 carrying an N-terminal methionine residue from *E. coli* expression (sequence in accession 3UD7_A). **25** is human FGF19 (mature form sequence of accession O95750) carrying an N-terminal methionine residue from *E. coli* expression.

### Fatty-Diacid Side Chain Synthesis

The fatty-diacid side chains were composed of a fatty diacid (C12, C14, C16, C18, and C20), a gGlu linker, and a small hydrophilic spacer containing two 2-(2-(2-aminoethoxy)ethoxy)acetyl elements (OEG-OEG), an ethylenediamine (C2DA), and finally an acetyl fragment deriving from the chemical handle (see Supporting Information K, Scheme S1).

Synthesis of fatty diacid-containing side chains (see example below) was performed by simple amide coupling reactions, either in solution or on solid support similar to methods previously described,^82^ but also including the addition of the ethylenediamine and Ac–Br elements before deprotection (see Supporting Information K, Scheme S2).

### Example of Side Chain Synthesis

The side-chain synthesis was performed as exemplified by the synthesis of C16-gGlu-OEG-OEG-C2DA-Ac–Br.

#### 5-{(S)-3-[2-(2-{[2-(2-{[2-(2-Bromo-acetylamino)-ethylcarbamoyl]-methoxy}-ethoxy)-ethylcarbamoyl]-methoxy}-ethoxy)-ethylcarbamoyl]-1-carboxy-propylcarbamoyl}-pentadecanoic Acid(2-amino-ethyl)-carbamic Acid Benzyl Ester Hydrochloride

A solution of N-(benzyloxycarbonyloxy) succinimide (ZOSu, 100 g, 401 mmol) in dichloromethane (500 mL) was added dropwise over 2 h to a solution of ethylenediamine (189 mL, 2.81 mol) in dichloromethane (750 mL). After 30 min, the suspension was filtered, and the solids were washed with dichloromethane. The filtrate was evaporated to dryness, and the residue was diluted with toluene (1.00 L) and water (0.50 L). The resulting mixture was filtered, and the filtrate was separated to afford two phases. The aqueous phase contained the product; therefore, it was extracted with dichloromethane (2 × 250 mL). All organic phases were combined, dried over anhydrous sodium sulfate, filtered, and concentrated in vacuo. The residue was diluted with toluene (750 mL) and extracted with 2 M aqueous hydrochloric acid (500 mL) and 1 M aqueous hydrochloric acid (100 mL). Acidic aqueous phases were combined and basified with a solution of sodium hydroxide (60.0 g, 1.50 mol) in water (90 mL). The resulting mixture was extracted with dichloromethane (4 × 200 mL), dried over anhydrous sodium sulfate, filtered, concentrated in vacuo, and diluted with hexane (200 mL); 4 M solution of hydrogen chloride in ether (100 mL, 400 mmol) was added to the solution, and the resulting suspension was concentrated in vacuo and diluted with hexanes (1.00 L). The precipitated solid was filtered, washed with hexanes, and dried in vacuo to give (2-amino-ethyl)-carbamic acid benzyl ester hydrochloride as a white powder.

#### Synthesis of 5-{(S)-3-[2-(2-{[2-(2-{[2-(2-Bromo-acetylamino)-ethylcarbamoyl]-methoxy}-ethoxy)-ethylcarbamoyl]-methoxy}-ethoxy)-ethylcarbamoyl]-1-carboxy-propylcarbamoyl}-pentadecanoic Acid

2-(7-Aza-1H-benzotriazole-1-yl)-1,1,3,3-tetramethyluronium hexafluorophosphate (HATU, 11.4 g, 30.1 mmol) and triethylamine (8.77 mL, 62.9 mmol) were subsequently added to a solution of (S)-22-(tert-butoxycarbonyl)-41,41-dimethyl-10,19,24,39-tetraoxo-3,6,12,15,40-pentaoxa-9,18,23-triazadotetracontanoic acid (C16(OtBu)-gGlu(OtBu)-OEG-OEG–OH) (22.4 g, 27.4 mmol), synthesized as described in ref (82) in dry dichloromethane (110 mL). Triethylamine (5.72 mL, 41.0 mmol) was added to a suspension of (2-amino-ethyl)-carbamic acid benzyl ester hydrochloride (6.94 g, 30.1 mmol) in dry dichloromethane (165 mL) and the resulting mixture was added to the above solution. The mixture was stirred at room temperature overnight, and then it was evaporated to dryness. The residue was redissolved in ethyl acetate (500 mL); washed with 1 M aqueous hydrochloric acid (2 × 200 mL), 5% aqueous solution of sodium carbonate (2 × 200 mL, very slow separation of phases), 1 M aqueous hydrochloric acid (8 × 200 mL), and brine; dried over anhydrous sodium sulfate and evaporated to dryness in vacuo. The residue was purified by flash column chromatography (Silicagel 60, 0.040–0.060 mm; eluent: dichloromethane/methanol 95:5) to afford 15-[(S)-3-(2-{2-[(2-{2-[(2-benzyloxycarbonylamino-ethylcarbamoyl)-methoxy]-ethoxy}-ethylcarbamoyl)-methoxy]-ethoxy}-ethylcarbamoyl)-1-tert-butoxycarbonyl-propylcarbamoyl]-pentadecanoic acid *tert*-butyl ester (C16(O*t*Bu)-gGlu(O*t*Bu)-OEG-OEG-C2DA-Z) as a pale yellow thick oil.

Yield: 23.84 g (88%).

RF (SiO_2_, dichloromethane/methanol 9:1): 0.35.

^1^H NMR spectrum (300 MHz, CDCl_3_, δH): 7.39–7.26 (m, 6 H); 7.19 (t, *J* = 6.3 Hz, 1 H); 6.91 (t, *J* = 5.7 Hz, 1 H); 6.52 (d, *J* = 7.5 Hz, 1 H); 5.83 (t, *J* = 5.5 Hz, 1 H); 5.09 (s, 2 H); 4.41 (ddd, *J* = 12.3, 4.6, and 4.3 Hz, 1 H); 3.99 (s, 2 H); 3.97 (s, 2 H); 3.71–3.30 (m, 20 H); 2.33–2.08 (m, 7 H); 1.97–1.83 (m, 1 H); 1.67–1.51 (m, 4 H); 1.45 (s, 9 H); 1.44 (s, 9 H); 1.35–1.20 (m, 20 H).

LC–MS purity: >99%.

LC–MS Rt (Kinetex 4.6 mm × 50 mm, acetonitrile/water 50:50 to 100:0 + 0.1% FA): 4.18 min.

LC–MS *m*/*z*: 994.9 (M + H)^+^.

Palladium on carbon (10%, 1.27 g, 1.20 mmol) was added to a solution of the above compound (23.8 g, 24.0 mmol) in methanol (350 mL) and the resulting mixture was hydrogenated at normal pressure for 4 h. The catalyst was filtered off and the filtrate was evaporated to dryness. The residue was evaporated several times from dichloromethane in order to remove residues of methanol and dried in vacuo to yield *tert*-butyl (S)-1-amino-25-(tert-butoxycarbonyl)-4,13,22,27-tetraoxo-6,9,15,18-tetraoxa-3,12,21,26-tetraazadotetracontan-42-oate (C16(O*t*Bu)-gGlu(O*t*Bu)-OEG-OEG-C2DA-H) as thick colorless oil.

Yield: 20.50 g (99%).

RF (SiO_2_, dichloromethane/methanol 9:1): 0.05.

^1^H NMR spectrum (300 MHz, CDCl_3_, δH): 7.54 (t, *J* = 5.7 Hz, 1 H); 7.41 (t, *J* = 5.6 Hz, 1 H); 7.14 (t, *J* = 5.5 Hz, 1 H); 6.68 (d, *J* = 7.5 Hz, 1 H); 5.25 (bs, 2 H); 4.39 (td, *J* = 8.3 and 4.2 Hz, 1 H); 4.01 (s, 4 H); 3.74–3.39 (m, 18 H); 2.96 (t, *J* = 5.7 Hz, 2 H); 2.34–2.06 (m, 7 H); 1.97–1.83 (m, 1 H); 1.68–1.50 (m, 4 H); 1.45 (s, 9 H); 1.43 (s, 9 H); 1.37–1.19 (m, 20 H).

LC–MS purity: > 99%.

LC–MS Rt (Kinetex 4.6 mm × 50 mm, acetonitrile/water 50:50 to 100:0 + 0.1% FA): 1.43 min.

LC–MS *m*/*z*: 860.8 (M + H)^+^.

N,N-Diisopropylethylamine (4.98 mL, 28.6 mmol) was added to a solution of the above amine (20.5 g, 23.8 mmol) in dry dichloromethane (290 mL) at −30 °C under argon. Bromoacetyl bromide (2.48 mL, 28.6 mmol) was added dropwise, and the resulting solution was stirred at 30 °C for an additional 3 h. The cooling bath was removed, the mixture was stirred at room temperature for 1 h, and then the solvent was removed in vacuo. The residue was redissolved in ethyl acetate (450 mL) and washed with a 5% aqueous solution of citric acid (300 mL). The phases were separated within 1 h. The organic layer was washed with water (300 mL) and the resulting emulsion was left to separate overnight to give three phases. The clear aqueous layer was removed, and the residual two phases were shaken with a saturated aqueous solution of potassium bromide (100 mL). The phases were left to separate overnight, the aqueous one was then removed, and the organic one dried over anhydrous sodium sulfate. The solvent was removed in vacuo and the residue was purified by flash column chromatography (Silicagel 60, 0.040–0.060 mm; eluent: dichloromethane/methanol 95:5) to afford *tert*-butyl (S)-1-bromo-28-(tert-butoxycarbonyl)-2,7,16,25,30-pentaoxo-9,12,18,21-tetraoxa-3,6,15,24,29-pentaazapentatetracontan-45-oate ((C16(O*t*Bu)-gGlu(O*t*Bu)-OEG-OEG-C2DA-Ac−Br)) as a colorless solid.

Yield: 19.46 g (83%).

RF (SiO_2_, dichloromethane/methanol 9:1): 0.25.

^1^H NMR spectrum (300 MHz, CDCl_3_, δH): 7.46 (m, 1 H); 7.33 (t, *J* = 5.9 Hz, 1 H); 7.21 (t, *J* = 5.1 Hz, 1 H); 6.92 (t, *J* = 5.2 Hz, 1 H); 6.50 (d, *J* = 7.5 Hz, 1 H); 4.41 (ddd, *J* = 12.2, 4.5, and 4.2 Hz, 1 H); 4.01 (s, 4 H), 3.85 (s, 2 H); 3.75–3.40 (m, 20 H), 2.36–2.08 (m, 7 H); 1.99–1.84 (m, 1 H); 1.68–1.51 (m, 4 H), 1.46 (s, 9 H); 1.44 (s, 9 H); 1.38–1.19 (m, 20 H).

LC–MS purity: >99%.

LC–MS Rt (Kinetex 4.6 mm × 50 mm, acetonitrile/water 50:50 to 100:0 + 0.1% FA): 3.51 min.

LC–MS *m*/*z*: 980.9, 982.9 (M + H)^+^.

The above compound (19.5 g, 19.8 mmol) was dissolved in trifluoroacetic acid (120 mL) and the resulting solution was stirred at room temperature for 1.5 h. Trifluoroacetic acid was removed in vacuo, and the residue was evaporated from dichloromethane (6 × 200 mL). Diethyl ether (200 mL) was added to the oily residue, and the mixture was stirred overnight to give a suspension. The solid product was filtered, washed with diethyl ether and hexanes, and dried in vacuo to afford the title product (C16-gGlu-OEG-OEG-C2DA-Ac–Br) as a white powder.

Yield: 16.74 g (97%).

^1^H NMR spectrum (300 MHz, AcOD-*d*_4_, δH): 4.61 (dd, J = 8.8 and 4.8 Hz, 1 H); 4.12 (s, 2 H), 4.10 (s, 2 H); 3.96 (s, 2 H); 3.77–3.39 (m, 20 H), 2.49–2.18 (m, 7 H); 2.16–1.04 (m, 1 H); 1.71–1.56 (m, 4 H), 1.30 (bs, 20 H).

LC–MS purity: >99%.

LC–MS Rt (Kinetex 4.6 mm × 50 mm, acetonitrile/water 50:50 to 100:0 + 0.1% FA): 3.51 min.

LC–MS *m*/*z*: 868.7, 870.7 (M + H)^+^.

Yield: 62.62 g (68%).

RF (SiO_2_, dichloromethane/methanol 4:1): 0.25 (free base).

^1^H NMR spectrum (300 MHz, AcOD-*d*_4_, 80 °C, δH): 7.42–7.26 (m, 5 H); 5.16 (s, 2 H); 3.60 (t, *J* = 5.7 Hz, 2 H); 3.32 (t, *J* = 5.7 Hz, 2 H).

### Preparation of FGF21 Analogs with a Fatty-Diacid Side Chain

The cysteamine-protected Cys-mutated FGF21 analogs were first treated with Bis(p-sulfonatophenyl)phenylphosphine (six equivalents) in a Tris, NaCl buffer at pH 8.0 to liberate the thiol of the cysteine (see example below). The side chains (five equivalents) were added to the mixture after deprotection (monitored by LC–MS). After the reaction overnight, the modified FGF21 analogs were isolated by anion exchange purification (typically a MonoQ 10/100 GL column). The product pool was concentrated and buffer-exchanged by UF/DF to a vehicle of 10 mM phosphate, 2% glycerol, pH 8.15, and 0.22 μm sterile filtered, and stored frozen.

### Example of Side Chain Conjugation to the FGF21 Backbone

#### S(Beta-180)-[2-[2-[[2-[2-[2-[[2-[2-[2-[[(4S)-4-carboxy-4-(15-carboxypentadecanoyl-amino)butanoyl] amino]ethoxy]ethoxy]acetyl]amino]ethoxy]ethoxy]acetyl]amino]-ethylamino]-2-oxoethyl]-Ala [Gln121, Leu168, Cys180]FGF21, **22**)

Tris in water was added to cysteamine protected Ala[Gln121, Leu168, Cys180]FGF21, **16** (80 mg, 0.0041 mmol), in Tris and NaCl-buffer (1.85 mg/mL) to adjust the pH to 8.1. BSPP (Bis(p-sulfonatophenyl)phenylphosphine dihydrate dipotassium salt, 13 mg) was added and stirred gently for 4 h at room temperature. 15-((S)-1-Carboxy-3-[2-(2-([2-(2-([2-(2-bromoacetylamino)ethylcarbamoyl]methoxy)ethoxy)ethylcarbamoyl]-methoxy)ethoxy)ethylcarbamoyl]propylcarbamoyl)pentadecanoic acid (C16-gGlu-OEG-OEG-C2DA-Br) (18 mg; 0.020 mmol) in ethanol (0.25 mL) was added. The pH was adjusted to 8.2 with Tris. After stirring gently overnight, MiliQ water (100 mL) was added to lower the conductivity to 2.9 mS/cm. The mixture was purified using anion exchange on a MonoQ 10/100 GL column using A-buffer: 20 mM Tris, pH 8.0; B-buffer: 20 mM Tris, 500 mM NaCl, pH 8.0, flow 5 mL and a gradient of 0–80% B over 60 CV. Yield: 46 mg, 56%.

LC–MS: Theoretical mass: 20,295.8: Found mass: 20,296.2.

### In Vitro Activity Characterization

Activation of the FGF receptor/KLB complex leads to activation of the MAPK/ERK signaling pathway and phosphorylation of ERK. The level of phosphorylated ERK (pERK) at a given time point increases with increasing concentrations of FGF21.

### Potency

The potency of the FGF21 analogs was determined in HEK293 cells transfected with human KLB. The HEK293/KLB cells were seeded in 96-well plates 2 days prior to the assay. On the day of the assay, a serial dilution of FGF21 analogs was added to the cells, and the plate was incubated at 37 °C for 12 min. The phosphorylation of ERK induced by the analogs was quantified using an AlphaScreen SureFire kit (cat. no. TGRES10K, PerkinElmer) and on an EnVision plate reader. To determine the impact of albumin on the in vitro potency of the FGF21 analogs, human serum albumin (HSA) was added to the dilution buffer at final assay concentrations of 0, 0.1, 1.0, and 1.5% and the potency was determined in HEK293/KLB cells as described above.

### FGF Receptor Selectivity

The selectivity of **15** for FGF receptor subtypes was determined in Ba/F3 cells with stable expression of human KLB in combination with FGFR1c, FGFR2c, FGFR3c, or FGFR4. To enhance FGFR3c and FGFR4 signaling in the Ba/F3 cells, chimer receptors of FGFR3c and FGFR4 were constructed exchanging the intracellular tyrosine kinase domain with that of FGFR1c (Supporting Information K [Method S1]). One day prior to the assay, the Ba/F3 cells were seeded in a medium supplemented with 0.02% Tween 20 and 10 μg/mL heparin. The addition of heparin is necessary as Ba/F3 cells do not produce the heparin required for optimal FGFR dimerization and signaling.^6,83,84^ The four different Ba/F3 cell lines were incubated with a serial dilution of FGF21 analogs for 15 min at 37 °C. FGF1 (**24**) and FGF19 (**25**) were used as positive control for Ba/F3/KLB/FGFR2c cells and Ba/F3/KLB/FGFR4 cells, respectively. The phosphorylation of ERK induced by the analogs was measured using an AlphaScreen SureFire kit (cat. no. TGRES10K, PerkinElmer) and an EnVision plate reader.

### Binding Affinity to FGF-Receptor Complexes

An AlphaScreen Binding Assay was used to determine the binding affinity of FGF21 analogs to FGFR1c/KLB, FGFR2c/KLB, FGFR3c/KLB, and FGFR4/KLB. Biotinylated FGF21 was coupled to streptavidin donor beads (PerkinElmer, cat. no. 6760002), and the ectodomain of human FGFR1c, FGFR2c, FGFR3c, or FGFR4 fused to Fc (R&D Systems, cat. no. 685-FR-050, 712-FR-050, 766-FR-050, and 658-FR-050) were coupled to Protein A acceptor beads (PerkinElmer, cat. no. 6760137M). A signal, detected as an increase in light at 520–620 nm, is generated when human BKL (R&D Systems, cat. no. 5889-KB) protein is added, bringing the donor and acceptor beads in close proximity. The assay is run in the presence of 0.1% ovalbumin which does not bind fatty acids. The signal (measured as counts per second) is measured using an EnVision plate reader. The signal can be inhibited by adding increasing doses of FGF21 analogs. This gives an indirect measure of binding affinity, and results are reported as IC_50_ values.

### Species Qualification

The species quantification of **15** was done in three stable cell lines (HEK293) overexpressing KLB of human, cynomolgus, and mouse origin. Selected clones responded to Met-FGF21 (**1**) with a similar magnitude of ERK phosphorylation across species, indicating a similar degree of KLB expression, allowing direct comparison between species. The potency of Met-FGF21 (**1**) and **15** was determined using pERK as a readout as described above (12 min of incubation at 37 °C).

All in vitro data were analyzed with nonlinear regression of sigmoidal dose–response curves using GraphPad Prism v 6.0 (GraphPad software, La Jolla, CA, USA).

More information on the cellular assays, DNA sequences for KLB, and generation of cell lines is provided in Supporting Information K [Methods S1–S4].

### In Vivo Experiments

All experimental procedures were conducted in accordance with internationally accepted principles for the care and use of laboratory animals and were conducted under a license from the Danish Animal Experiments Inspectorate.

### Body Weight Effects in Lean and DIO Mice

All animals had access to shelter, nesting material, and chewing sticks and were acclimatized and used to handling at least 1 week prior to any experiments. The mice were housed according to Novo Nordisk standards (Housing of experimental animals at Novo Nordisk A/S) and had ad libitum access to food and tap water under controlled lighting (12 h:12 h light/dark cycle; lights on 6 a.m.), temperature (21 ± 3 °C), and humidity (50 ± 20% relative humidity) conditions. One day prior to the start of dosing, animals were stratified into groups matched for body weight.

The effect of FGF21 analogs was determined in lean C57BL/6J male mice (body weight approximately 24 g) or in DIO C57BL/6J male mice (body weight approximately 49 g) fed a high-fat diet (RD12492, 60% kcal fat, Research Diets, USA) for 16 weeks). The FGF21 analogs were subcutaneously administered once or twice daily. The FGF21 analogs were formulated in 10 mM phosphate, 2% (w/vol) glycerol, 500 ppm of polysorbate 80, pH 8.15.

Data were analyzed by two-way ANOVA or Student’s *t* test using GraphPad Prism v 6.0 (GraphPad software, La Jolla, CA, USA).

### Pharmacokinetic Characterization

Pharmacokinetic characterization was conducted using iv or sc administration. All studies were conducted with the API dissolved in a phosphate buffer (10 mM phosphate and 2% glycerol, adjusted to pH 8.15). In mice, the formulation also contained 500 ppm of polysorbate 80.

### Pharmacokinetics in iv-Dosed Mice

Mouse PK was determined in lean male C57BL/6J mice by using a sparse sampling regimen. C57Bl/6J mice (Taconic Europe, Denmark) weighing approximately 30 g were housed according to Novo Nordisk standard procedures. Mice were dosed 20 mg/kg iv using a syringe and a 26G needle. This was performed in nonfasted mice and was based on sparse sampling with collection of blood samples at 0 and 5 min, and at 1, 2, 4, 8, 12, 24, and 48 h post-dose. Each mouse contributed two to three blood samples over the course of the sampling period. This way, a full plasma concentration curve was obtained with a contribution from the collective.

### Pharmacokinetics in iv-Dosed Minipig

Female Göttingen minipigs (Ellegaard, Denmark), approximately 8–9 years of age and weighing 17–20 kg were implanted with peripherally inserted central venous catheters positioned in the *vena jugularis* for blood sampling. Minipigs were dosed with 5 nmol/kg of **15**, using the central venous catheter, and a full plasma concentration versus time profile was obtained from each animal for up to 11 days. Plasma samples were collected at 0, 0.083, 0.25, 0.5, 0.75, 1, 1.5, 2, 3, 4, 6, 8, 10, 24, 48, 72, 96, 120, 168, 192, 216, 240, and 264 h post-dose. A PK and metabolite identification study was conducted in minipigs receiving Met-FGF21 at 20 mg/kg dose (two animals). Plasma samples were collected at 0, 0.083, 0.25, 0.5, 1, 2, 4, 6, 8, 10, 12, 24, 30, 48, 54, and 72 h post-dose.

### Pharmacokinetics in sc- and iv-Dosed LYD Pigs

Crossbred LYD female pigs (Gundso̷gård, Denmark), weighing 70–80 kg were implanted with venous catheters in the *vena cava caudalis* for blood sampling. LYD pigs were dosed with 5 nmol/kg, using the central venous catheter for iv and a NovoPen 4 with needle NovoFine 30G for sc dosing. Full plasma concentration versus time profile was obtained from each animal for up to 11 days. Plasma samples were collected at 0, 0.083, 0.25, 0.5, 0.75, 1, 1.5, 2, 3, 4, 6, 8, 10, 24, 30, 48, 72, 96, 120, 168, 192, 216, 240, and 264 h post-dose.

### Pharmacokinetics in sc- and iv-Dosed Monkeys

Purpose-bred non-naïve Vietnamese cynomolgus monkeys (*macaca fascicularis*) were obtained from Huntingdon Life Sciences (HLS) stock, and animals were returned to stock at HLS after the study. The monkeys were approximately 33–40 months of age and weighed 2.8–3.9 kg. Animals were housed and maintained within the Department of Primate Toxicology, HLS, according to the standard operating procedures of that department. On the day of dosing, animals received a 5 nmol/kg iv bolus injection in the cephalic or saphenous vein with a syringe and a 25G butterfly needle. For sc administration, a bolus injection was given into the thoracic or lumbar region at a dose of 15 mg/kg using a syringe and a 23G needle. Full plasma concentration versus time profile was obtained from each animal for up to 10 days post-dosing. Blood samples were collected at 0, 0.083, 0.25, 0.5, 1, 3, 6, 8, 10, 12, 18, 24, 36, 48, 72, 96, 120, 144, 168, 192, 216, and 240 h post-dose.

For all studies, blood samples were collected in EDTA-containing tubes and EDTA plasma was stored at −20 °C after centrifugation until plasma analysis. For all studies, an ELISA assay (Biovendor R&D, cat. no. RD191108200R) was used for the determination of FGF21 in plasma. For in vivo metabolite studies, LC–MS was used for the determination of FGF21 analogs and the identification of metabolites.

For all pharmacokinetic studies, the plasma concentration versus time profiles were analyzed by a noncompartmental analysis using Phoenix WinNonlin Professional 6.3 (Pharsight, Mountain View, CA, USA). In mice, sparse sampling-based calculation was used, and in other species, calculations were performed using individual concentration versus time values. The AUC was calculated using the Linear Log Trapezoidal method. The PK parameters are presented as the arithmetic mean and standard error of the mean, except *t*_1/2_, which is presented as the harmonic mean.

### In Vitro and In Vivo Proteolytic Stability

#### DPP-IV Stability of −1–31 and 1–31 Peptides

Ten μM of peptide was incubated in duplicate with recombinant DPP-IV (2 μg/mL, R&D Systems, cat no. 1180-SE) at 37 °C. The incubation buffer was a PBS buffer with 0.0005% Tween20 and 0.001% BSA pH 7.4. The −1–31 and 1–31 peptides represented the N-terminal region of hFGF21. The sequences were as follows: −1–31: AHPIPDSSPLLQFGGQVRQRYLYTDDAQQTEA and 1–31: HPIPDSSPLLQFGGQVRQRYLYTDDAQQTEA. Native GLP-1 (7–37) is a well-known DPP-IV substrate and was used as a positive control. The *t*_1/2_ of GLP-1 was 16 min in this assay. Incubations were stopped at 0.5, 3, 10, 20, 30, 60, 120, and 180 min by the addition of three volumes of ethanol containing 40 nM of internal standard. Samples were diluted with three volumes of water containing 1% formic acid. Parent peptides were quantified by LC–MS and DPP-IV metabolite formation was verified for unstable compounds. LC–MS was performed on a TLX-2 system (Germering, Germany) that was used in one-column mode coupled with Q-Exactive HF (ThermoScientific, Bremen, Germany) operated in positive ionization mode in a scan-dependent mode. The chromatographic conditions were similar to those described for in vitro degradation experiments with FAP.

#### In Vitro Degradation of (1), (15), and (5) by FAP

Five μM of FGF21 analog was incubated in duplicate with FAP (R&D Systems, 3715-SE-010) at pH 7.4 (50 mM Tris, pH 7.4, 100 mM NaCl, 0.05% Tween20) at 37 °C. Incubations were conducted at ±0.1% BSA. The enzyme-to-substrate ratio was 1:20 (w/w). Samples were taken at 2, 10, 20, 30, 60, 120, and 180 min. Reactions were stopped by adding 90 μL of 20% acetonitrile (1% formic acid) to 30 μL of the sample. All samples were analyzed by LC–MS for the quantification of FGF21 analog and confirmation of −1–171 metabolite formation. LC–MS was conducted on an Acquity ultraperformance liquid chromatography (UPLC) iClass system (Waters, Milford, MA) connected to a QExactive Plus (ThermoScientific, Bremen, Germany) operated in positive ionization mode (full scan *m*/*z* 400–2,000). UPLC conditions were as follows: a Waters Acquity UPLC Peptide HSS T3 column (1.8 μm, 100 A, 1 × 50 mm ID) was set at 50 °C, and the following gradient was used: 0–2 min 20% B, 2–20 min: 20–35% B with a flow at 0.3 mL/min.

#### LC–MS Analysis of In Vivo Metabolites

Fifty μl of acetonitrile was added to 50 μL of plasma. After centrifugation, the resulting supernatant was diluted with five volumes of water; 10 μL of the sample was injected onto the LC–MS equipment. Plasma standards of **15** and synthetic standards of 152–181 metabolite and 172–181 metabolite were prepared at 0.05, 0.2, 1, 5, and 20 μM, and analyzed before and after the animal samples. For the analysis of **5**, the standards only contained parent protein and not metabolites. LC–MS analysis was performed on a Waters Acquity UPLC system (Milford, MA) and Maxis 4G Q-TOF from Bruker Daltonics (Bremen, Germany). The mobile phases consisted of 1% formic acid in water and 1% formic acid in acetonitrile. The UPLC column was a Cortecs column (1.6 μm, 2.1 mm ID × 50 mm) from Waters. The flow rate was 0.3 mL/min and the column temperature was 60 °C. The following gradient was used: 0–2.9 min: isocratic at 5% B, 2.9–3.0 min: 5–15%, 3.0–31.5 min: 15–40% B, 31.5–32.5 min: 40–100% B, 31.6–32.5 min: 100% B. Finally, the column was re-equilibrated with 5% B prior to the next run. MS was conducted in positive ionization mode by electrospray ionization from *m*/*z* 300–1,850.

#### Identification of Metabolites

Average MS spectra from one-min intervals were deconvoluted (MaxEnt3) and intact, monoisotopic masses were aligned (within ±5 ppm) with the sequence of parent FGF21 analog in GPMAW (Lighthouse Data, Odense, Denmark, vs 9.51). MS/MS spectra were acquired for metabolites if there was more than one option. For metabolites with a lower molecular weight (approximately 6,000–8,000 Da and below), metabolites were identified using the software packages Biotools and Sequence Editor, both from Bruker Daltonics (version 3.2).

#### Immunopurification of Plasma Samples for MS/MS Experiments

An MS/MS experiment was performed to verify the identity of the −1–171 metabolite of **5**. Fifteen micrograms of biotinylated antibody (anti-H-FGF21 capture, R&D Systems BAF2539) were added to 100 μL of plasma to which 80 μL of sample buffer and 320 μL of PBS were added. The sample buffer consisted of Tris-buffered saline (50 mM Tris, pH 7.4, 100 mM NaCl, 1% Tween). After a 2 h incubation at 37 °C, incubation with MyOne T1 streptavidin magnetic beads (Invitrogen 65601) at room temperature was conducted on a KingFisher Flex. Magnetic beads were washed, and the final elution was performed in 240 μL of 20% acetonitrile containing 1% formic acid and 0.01% BSA. Samples were analyzed as described for FAP incubation but with full scan MS from *m*/*z* 200–2,000 and parallel reaction monitoring (PRM) transitions of the four most abundant charge states.

### RP-UPLC and SE-HPLC Analysis of FGF21 Formulations and Compounds for In Vitro/In Vivo Analysis

#### RP-UPLC Determination of Purity and Content

Reverse-phase UPLC (RP-UPLC) analysis for purity and content was performed on a Waters (Milford, MA) UPLC system with UV detection at 215 nm. The eluent system was (A) 0.1% (v/v) trifluoroacetic acid (TFA) in Milli-Q water and (B) 0.1% (v/v) TFA in 90% (v/v) acetonitrile. FGF21 compound samples were injected onto an Acquity UPLC BEH Shield RP18 Column, 2.1 × 150 mm, 1.7 μm, 130 Å (part number 186003376) from Waters, held at 60 °C, using a flow rate of 0.4 mL/min with a main gradient of 37–44% B over 23 min and a total runtime of 33 min. For **15** formulations, a compound-specific method was used with an Acquity UPLC Peptide CSH C18 Column, 2.1 × 150 mm, 1.7 μm, 130 Å (part number 186006938) held at 60 °C, using a flow rate of 0.35 mL/min with a main gradient of 35–42% B over 35 min and a total runtime of 45 min. Purity, as assessed by RP-UPLC, was evaluated as the main peak area in relation to the total area of all peptide-related peaks. Purity was ≥90% using this high-resolution RP-UPLC method (Supporting Information I and J; Table S9 and Figure S9).

#### SE-HPLC Determination of Purity and HMWP Content

Size-exclusion high-performance liquid chromatography (SE-HPLC) for the determination of the relative content of covalent high molecular weight proteins (HMWP), as well as of purity, was done using a Waters Alliance HPLC system equipped with a Waters Insulin HMWP column (7.8 × 300 mm; part number WAT201549, Waters) at 50 °C with a flow rate of 0.5 mL/min and UV detection at 215 nm. Elution was performed under isocratic conditions (30 min runtime) with a mobile phase prepared in Milli-Q water: 0.5 M NaCl, 10 mM sodium dihydrogen monohydrate, 5 mM ortho-phosphoric acid, and 50% (v/v) 2-propanol. All compounds were evaluated in vivo. Purity was ≥99% according to SE-HPLC (Supporting Information I and J; Table S10 and Figure S10).

#### Trypsin Peptide Map and LC–MS of FGF21 Analogs

Trypsin (sequence grade Promega) dissolved in 100 mM ammonium bicarbonate pH 8.0 was added to an FGF21 analog solution (∼1 mg/mL) to a ratio of 1/100 (w/w). The solution was vortex-mixed and spun down. The sample was incubated for approximately 18 h in a heat-block (Eppendorf) at 37 °C with mixing at 400 rpm. After incubation, the reaction was stopped by adding 7 μL of 5% TFA/40 μL of FGF21 analog solution. The solution was vortex-mixed and spun down.

The digest was analyzed by liquid chromatography elevated energy mass spectrometry (LC–MS^E^) using an LC–MS Synapt G2 (Waters) with a Waters UPLC system. The peptide mixture was separated on an Acquity CSH C18 column (1.0 × 150 mm, 1.7 μm, 130 Å, part number 186005294) held at 55 °C using a linear gradient of 0–50% eluent B with a flow rate of 0.1 mL/min and a total runtime of 30 min. Eluent A: 0.1% formic acid, Eluent B: 0.1% formic acid in acetonitrile. Detection by UV at 215 and by MS and MS^E^ (ES+, mass range 100–4,000 Da continuum, cone voltage 25 V, resolution mode, capillary voltage 3 kV, source temperature 120 °C, cone gas flow 25 L/h, MS^E^ cone voltage ramp 25–45 V). Data were analyzed manually in MassLynx compared with theoretical digests (the theoretical tryptic peptides of Met-FGF21 and **15** are listed in Supporting Information F [Table S5] and H [Table S7]) and automatically with BiopharmaLynx. Peptides were identified by LC–MS and LC–MS^E^. For the identification of isomers, tryptic peptides of Asp isomers were synthesized.

### Biophysical Characterization

#### Solubility

Ten mg/mL **15** in 10 mM phosphate with 2% glycerol was used to create a pH series from 4.5 to 8.5 in half of the pH steps. After pH adjustment, the samples were left overnight at ambient room temperature. On the following day, the samples were centrifuged and the concentration in the supernatant was determined by UV measurement at 280 nm. For samples having a recovery larger than 90%, DLS measurements were carried out using a DynaPro Plate Reader (Wyatt Technology Corp., Santa Barbara, CA, USA). Triplicates of 30 μL of each sample were added to a 384-well plate Corning 3540 (Corning, NY, USA). The plate was centrifuged for 5 min at 1,200 rpm before analysis to remove any air bubbles from the wells. Each well was measured 40 times with 5 s acquisition time. Dynamics software version 7.1.8 (Wyatt Technology Corp., Santa Barbara, CA, USA) was used to collect and analyze the data.

#### pH-Dependent Interaction

DLS data were acquired at 25 °C by adding 30 mL of each sample in triplicates to a 384-well plate Corning 3540 (Corning, NY, USA). Each well was measured 40 times with 5 s acquisition time. A DynaPro Plate Reader and Dynamics software version 7.1.8 (Wyatt Technology Corp., Santa Barbara, CA, USA) was used to collect and analyze the data. The interaction parameter, kD, was calculated as the slope of diffusion coefficient *D* versus concentration divided by *D*_0_ (the extrapolation of *D* to zero concentration). A sample series of 1–15 mg/mL was prepared by dilution of stock solutions. Samples were filtered through 0.1 μm prior to analysis.

#### pH-Dependent Thermal Stability

The study of heat-induced unfolding of FGF21 **15** was performed by Capillary DSC using a MicroCal VP-Capillary DSC from Malvern instruments (UK). The final concentration was 5 mg/mL in 10 mM phosphate, pH 7.2–8.2 with 2% glycerol. Each pH was subjected to a temperature ramp from 20 to 110 °C with a heating rate of 200 °C/h, and specific heat capacity as a result of heat-induced unfolding was constantly measured and recorded. The corresponding sample buffer was in the reference cell. A buffer–buffer reference scan was subtracted from each sample scan prior to data analyses. Obtained endothermic peaks were analyzed in MicroCal Analysis software (Malvern Instruments, UK) and the temperature of the midpoint of thermal transition was determined.

#### High-Concentration Self-Association

Asymmetrical flow-field flow fractionation (AF4) was performed on an Eclipse DualTec instrument (Wyatt Technology Europe GmbH, Germany). The AF4 system was coupled to an Agilent 1200 series HPLC system (Agilent Technologies, Santa Clara, CA, USA), which consisted of a 1260 isocratic pump, UV detector, and autosampler. An 18-angle static light scattering detector (DAWN HELEOS) and a differential refractive index detector (Optilab T-rEX), both from Wyatt Technology Corporation, Santa Barbara, CA, USA, were coupled to the system. A short channel (effective length 145 mm) with a spacer height of 490 mm (W490) and regenerated cellulose membranes with a cutoff of 10 kDa (Wyatt Technology Europe GmbH, Germany) as the bottom semi-permeable wall was used. One μL of concentrated samples and 10 μL of diluted samples (5 mg/mL) were injected. Focusing was performed with a cross-flow of 1.5 mL/min for 1 min. This was followed by separation at a detector flow of 1 mL/min and constant cross-flow of 2 mL/min for 20 min. Finally, a 5 min period without cross-flow and detector flow at 1 mL/min was applied to elute the remaining sample. ASTRA software, version 7.3.2.19, was used for analysis.

#### Immunoassay for Analysis of FGF21 Analogs in Plasma

Plasma samples were analyzed with a kit from BioVendor (RD191108200R). The procedure for the kit was followed with modifications (own standards and control samples prepared for each analog).

## Supporting Information Available

Proteolytic degradation of Met-FGF21 in mice and minipig; DPP-IV metabolism of N-terminal of FGF21; statistical strength of in vitro potency data shown in Table 2; statistical strength of data shown in Table 3; lowering of body weight in lean and DIO mice; proteolytic degradation of **15** in mice and minipig; identification of metabolites of **5**; theoretical trypsin peptide maps of Met-FGF21 (**1**) and **15**; compound purity data; UPLC traces of **1**, **4**, **15**, **16, 20**, **21**, **22**, and **23**; and supplementary methods (PDF)

## Abbreviations

ADA: antidrug antibody
AF4: asymmetrical flow-field flow fractionation
C2DA: ethylenediamine
D2/D3: immunoglobulin-like binding domains of FGFR1c and FGFR3c
DIO: diet-induced obese
DLS: dynamic light scattering
DPP-IV: dipeptidyl peptidase IV
DSC: differential scanning calorimetry
Fab: fragment antigen-binding
FAP: fibroblast activation protein
Fc: fragment crystallizable
gGlu: gamma-glutamic acid
HATU: 2-(7-aza-1H-benzotriazole-1-yl)-1,1,3,3-tetramethyluronium hexafluorophosphate
HLA: human leukocyte antigen
HMWP: high molecular weight protein
kD: interaction parameter for DLS
KLB: beta-klotho
LC–MS^E^: liquid chromatography elevated energy mass spectrometry
LYD: Landrace, Yorkshire, and Duroc
MALS: multi-angle-light-scattering
MASH: metabolic dysfunction-associated steatohepatitis
Met-FGF21: human FGF21 with N-terminal Met extension
NHP: nonhuman primate
OEG: 2-(2-(2-aminoethoxy)ethoxy)acetyl
pEC_50_: negative logarithm of EC_50_
pERK: phosphorylation of extracellular signal-regulated kinase
pIC_50_: negative logarithm of IC_50_
Rh: hydrodynamic radius
SE-HPLC: size-exclusion high-performance liquid chromatography
TEA: triethylamine
TG: triglyceride
Tm: midpoint of thermal transition
UPLC: ultraperformance liquid chromatography
Vd: volume of distribution
