# Supplementary material for: Development of Zalfermin, a Long-Acting Proteolytically Stabilized FGF21 Analog
Source: J Med Chem. 2024 Jul 16;67(14):11769–88. doi: 10.1021/acs.jmedchem.4c00391 (PMC11284795; doi:10.1021/acs.jmedchem.4c00391)
Supplement: Supplementary file 1 — jm4c00391_si_001.pdf [file jm4c00391_si_001.pdf]

Supporting Information

Development of Zalfermin, a Long-Acting Proteolytically Stabilized FGF21 Analog

Kristian Sass-Ørum[1*], Tina Møller Tagmose[1], Jørgen Olsen[1], Annika Sjölander[1], Per-Olof Wahlund[1], Dan Han[3], Andreas Vegge[2], Steffen Reedtz-Runge[1], Zhe Wang[3], Xiang Gao[3], Birgit Wieczorek[1], Kasper Lamberth[2], Kirsten Lykkegaard[2], Peter Kresten Nielsen[1], Henning Thøgersen[1], Mingrui Yu[3], Jianhua Wang[3], Jørn Drustrup[1], Xujia Zhang[3], Patrick Garibay[1], Kristian Hansen[2], Ann Maria Kruse Hansen[2], Birgitte Andersen[2]

[1] Novo Nordisk A/S, Global Research Technologies, DK-2760 Maaloev, Denmark

[2] Novo Nordisk A/S, Global Drug Discovery, DK-2760 Maaloev, Denmark

[3] Novo Nordisk A/S, Novo Nordisk Research Center China, Beijing 102206, China

Contents

SI A: Proteolytic Degradation of Met-FGF21 in Mice and Minipig 4

Figure S1A. Identification of Metabolites of Met-FGF21 (1) In Minipigs Dosed with Met-FGF21 (1) (20 mg/kg, iv). 4

Figure S1B. Identification of Metabolites of Met-FGF21 (1) in Mice dosed with Met-FGF21 (1) (20 mg/kg, sc). 4

SI B: DPP-IV Metabolism of N-terminal of FGF21 5

Figure S2. LC-MS Analysis of 1–31 Peptide Fragment of hFGF21 Following Incubation with DPP-IV (3 h). 5

SI C: Statistical Strength of In Vitro Potency Data Shown in Table 2 6

Table S1. Statistical Strength of In Vitro Potency Data Shown in Table 2. 6

Table S2. FGFR1c/KLB AlphaScreen Binding Data For Results Shown in Figure 3. 7

SI D: Statistical Strength of Data Shown in Table 3 8

Table S3. Statistical Strength of In Vitro Potency Data shown in Table 3. 8

Table S4. Statistical Strength of t½ and In Vivo Potency Data Shown in Table 3. 9

Figure S3. Effect of Albumin on In Vitro Potency of FGF21 Compounds with Increasing Length of Fatty Diacid Sidechain. 10

Figure S4. FGF21-induced ERK-phoshorylation in Ba/F3 cells overexpressing FGFR1c/KLB, FGFR2c/KLB, FGFR3c/KLB, and FGFR4/KLB 11

SI E: Lowering of Body Weight in Lean and DIO Mice 12

Figure S5. Effect of 15 and 23 in DIO mice. 12

Figure S6. 15 Dose Response in DIO mice. 12

SI F: Proteolytic Degradation of 15 in Mice and Minipig 14

Table S5. Identification of Metabolites for 15 Following Dosing in Mice (20 mg/kg, iv). 14

Figure S7. Exposure of 15 and The Most Abundant Metabolite 152–181 in Minipig (1 mg/kg, iv) 14

SI G: Identification of Metabolites of 5 15

Table S6. Identification of Metabolites of 5 Dosed to Mice (20 mg/kg, iv). 15

Figure S8. MS/MS spectra of A) 5 (analog with non-engineered FGF21 C-terminal region), and B) FAP metabolite (–1–171) identified in plasma samples from mice (20 mg/kg, iv). 16

SI H: Theoretical Trypsin Peptide Maps of Met-FGF21 (1) and 15 17

Table S7. Theoretical Trypsin Peptide Map of Met-FGF21 (1). 17

Table S8. Theoretical Trypsin Peptide Map of 15. 17

SI I: Compound Purity Data 18

Table S9. Purity of FGF21 Analogs as Determined by RP-UPLC. 18

Table S10. Purity of FGF21 Analogs as Determined by SE-HPLC. 18

SI J: RP-UPLC and SE-HPLC Traces of 1, 4, 15, 16, 20, 21, 22, and 23 19

Figure S9. Chromatograms for FGF21 Analog Purity (RP-UPLC) Determinations shown in Table S9. 19

Figure S10. Chromatograms for FGF21 Analog Purity (SE-HPLC) Determinations shown in Table S10. 21

SI K: Supplementary Methods 24

Scheme S1. Albumin-Binding Sidechains: C12, C14, C16, C18, and C20-gGlu-OEG-OEG-C2DA-Ac–Br. 24

Scheme S2. Synthesis of Sidechains. 24

Method S1. Generation of HEK293 KLB Cell Lines 25

Method S2. In Vitro Potency Assay Description in HEK293 26

Method S3. Generation of Ba/F3 Cell Lines Expressing KLB/FGFR1c, KLB/FGFR2c, KLB/FGFR3c, and KLB/FGFR4 27

Method S4. Receptor Selectivity Assay with Ba/F3 Cells 31

SI A: Proteolytic Degradation of Met-FGF21 in Mice and Minipig

Figure S1A. Identification of Metabolites of Met-FGF21 (**1**) In Minipigs Dosed with Met-FGF21 (**1**) (20 mg/kg, iv).

The deconvoluted spectrum is from LC-MS analysis of a plasma sample taken 2 h after dosing. Theoretical and experimentally determined masses deviate by less than ±2 ppm. Met-FGF21: human FGF21 with N-terminal Met extension.

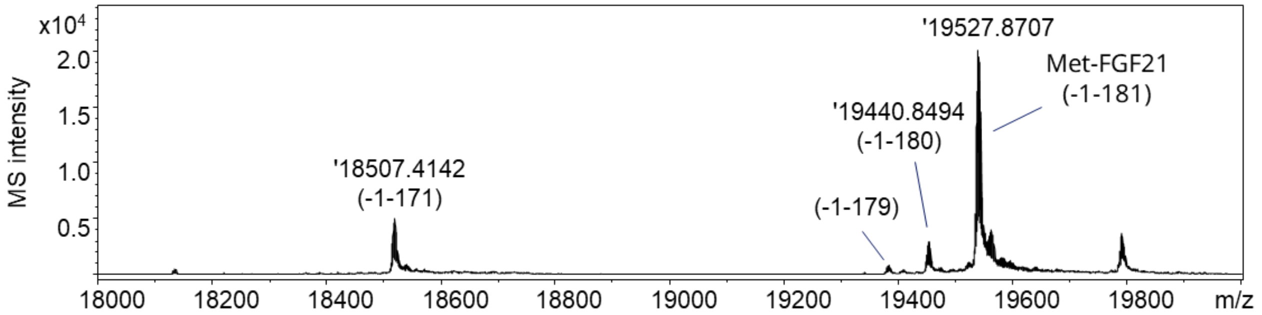

Figure S1B. Identification of Metabolites of Met-FGF21 (**1**) in Mice dosed with Met-FGF21 (**1**) (20 mg/kg, sc).

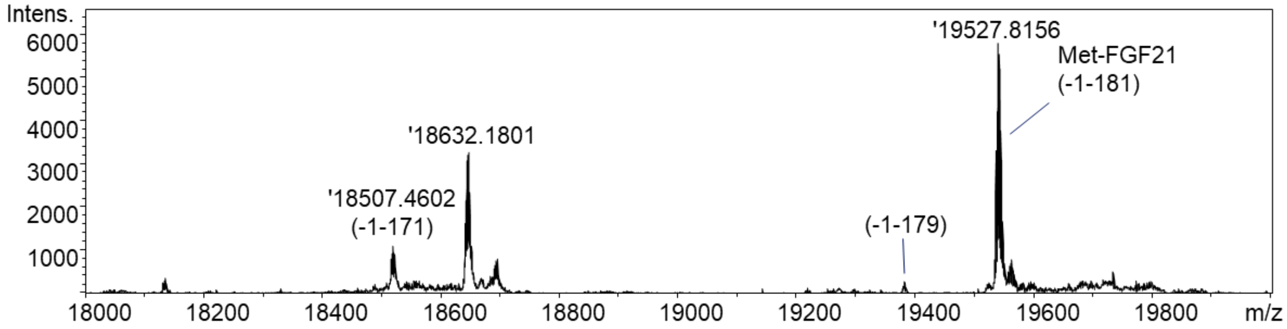

The deconvoluted spectrum is from LC-MS analysis of a plasma sample taken 3 h after dosing. Theoretical and experimentally determined masses deviate by less than ±2 ppm. Met-FGF21: human FGF21 with N-terminal Met extension.

SI B: DPP-IV Metabolism of N-terminal of FGF21

Figure S2. LC-MS Analysis of 1–31 Peptide Fragment of hFGF21 Following Incubation with DPP-IV (3 h).

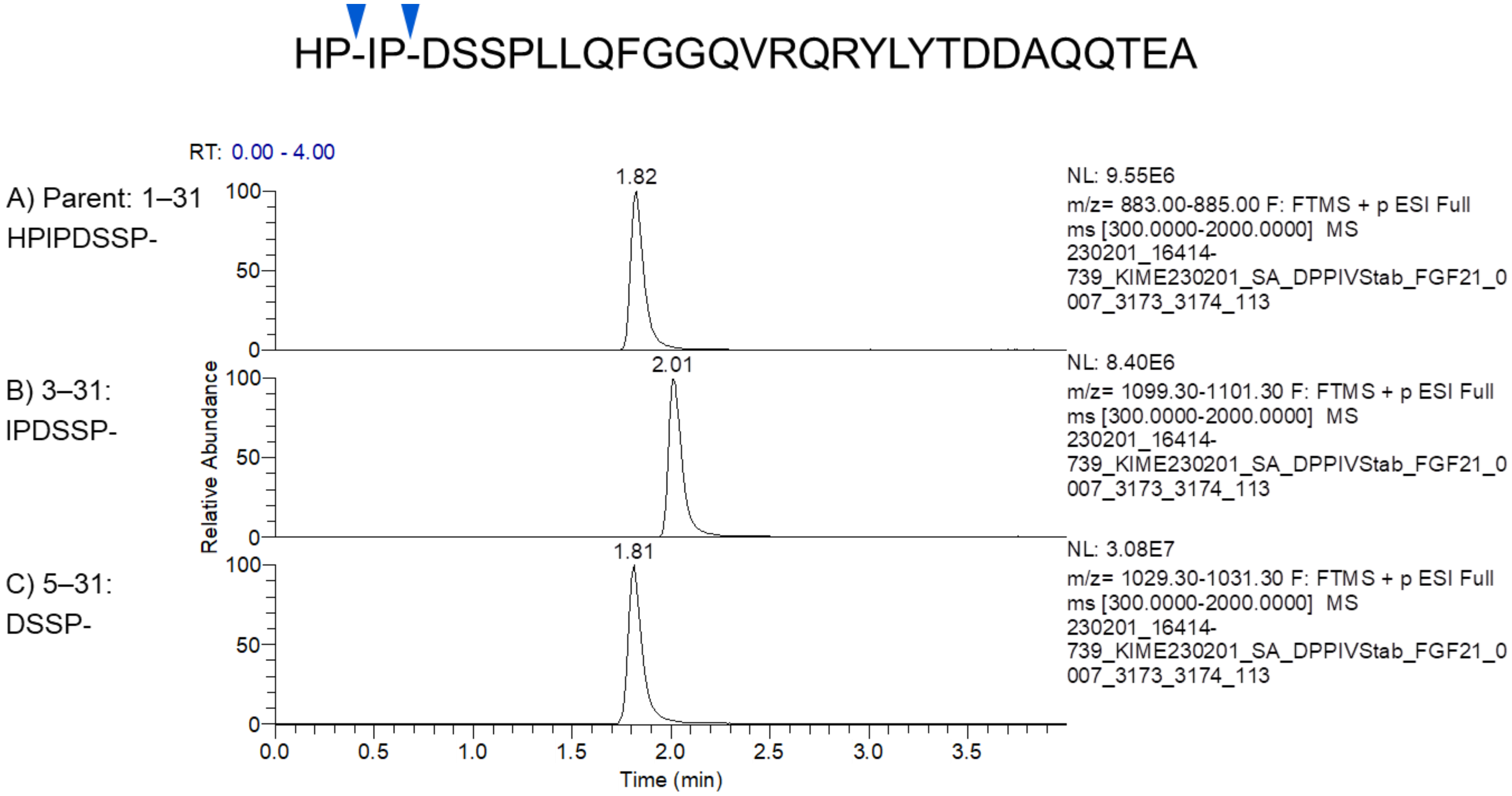

A) Intact 1–31 peptide (charge state 4). B) DPP-IV cleavage to form 3–31 (charge state 3). C) 5–31 peptide (charge state 3) from the second DPP-IV cleavage. DPP-IV: dipeptidyl peptidase IV hFGF21: human FGF21.

SI C: Statistical Strength of In Vitro Potency Data Shown in Table 2

Table S1. Statistical Strength of In Vitro Potency Data Shown in Table 2.

In Vitro Potency of FGF21 Analogs Measured as Phosphorylation of ERK in HEK293 Cells Transfected with Human KLB.

Compound	Mutations	Modification		Potency EC_{50} (nM)	Potency pEC_{50}	SD[b]	Number of experiments
		Site	Type				
FGF21 (**0**)				1.0	9.0	0.2	4
**1**	-1M			1.6	8.8	0.2	211
**2**	-1A			1.3	8.9	0.3	5
**3**	-1A, 121Q			1.0	9.0	9.0; 9.0	2
**4**	-1A, 121Q, 168L			2.0	8.7	0.2	23
**5**	-1A, 71C, 121Q, 168L	71C	Fatty diacid[a]	4.8	8.3	8.2; 8.5	2
**6**	-1A, 121Q, 167C, 168L	167C	Cysteamine	1.4	8.8	8.9; 8.8	2
**7**	-1A, 121Q, 168L, 170C	170C	Cysteamine	3.4	8.5	8.4; 8.5	2
**8**	-1A, 121Q, 168L, 171C	171C	Cysteamine	2.2	8.6	8.6; 8.7	2
**9**	-1A, 121Q, 168L, 172C	172C	Cysteamine	3.3	8.5	8.4; 8.5	2
**10**	-1A, 121Q, 168L, 174C	174C	Fatty diacid[a]	1.5	8.8	0.3	4
**11**	-1A, 121Q, 168L, 174C	174C	Cysteamine	6.1	8.2	8.1; 8.3	2
**12**	-1A, 121Q, 168L, 176C	176C	Cysteamine	160	6.8	6.6; 6.9	2
**13**	-1A, 121Q, 168L, 178C	178C	Cysteamine	90	7.0	7.3; 6.8	2
**14**	-1A, 121Q, 168L, 179C	179C	Cysteamine	67	7.2	7.1; 7.3	2
**15**	-1A, 121Q, 168L, 180C	180C	Fatty diacid[a]	3.3	8.5	0.2	50
**16**	-1A, 121Q, 168L, 180C	180C	Cysteamine	2.5	8.6	0.2	19
**17**	-1A, 121Q, 168L, 180C, des181	180C	Fatty diacid[a]	3.9	8.4	0.4	4
**18**	-1A, 121Q, 168L, 181C	181C	Fatty diacid[a]	39	7.4	0.2	3
**19**	-1A, 121Q, 168L, 181C	181C	Cysteamine	24	7,6	0.4	12

[a]C18 diacid gGlu-OEG-OEG-C2DA-Ac sidechain. [b]Individual measurements are shown when $n < 3$. KLB: beta-klotho; pEC_{50}: negative logarithm of EC_{50}; SD: standard deviation.

Table S2. FGFR1c/KLB AlphaScreen Binding Data for Results Shown in Figure 3.

Compound	Mutations	Modification		IC_{50}, nM	pIC_{50}	SEM pIC_{50}	Number of experiments
		Site	Type				
**1**	-1M			277	6.56	0.89	7
**4**	-1A, 121Q, 168L			663	6.18*	0.10	7
**15**	-1A, 121Q, 168L, 180C	180C	Fatty diacid[a]	750	6.13**	0.08	7
**17**	-1A, 121Q, 168L, 180C, des181	180C	Fatty diacid[a]	1622	5.79***, [b]	0.05	4
**18**	-1A, 121Q, 168L, 181C	181C	Fatty diacid[a]	>2000	5.72***, [c]	0.02	4

*$p < 0.05$, **$p < 0.01$, ***$p < 0.001$, unpaired t-test vs **1**; [a]C18 diacid gGlu-OEG-OEG-C2DA-Ac sidechain; [b]$p < 0.05$, unpaired t-test vs **15;** [c]$p < 0.05$ unpaired t-test vs **4.** pIC_{50}: negative logarithm of IC_{50}; SEM: standard error of the mean.

SI D: Statistical Strength of Data Shown in Table 3

Table S3. Statistical Strength of In Vitro Potency Data shown in Table 3.

The In Vitro Potency of FGF21 Analogs in the Presence of 0, 0.1, 1.0, or 1.5% HSA was Measured as Phosphorylation of ERK in HEK293 Cells Transfected with Human KLB.

			**Potency (EC_{50}, nM)**											
**Compound**	**Mutations**	**Modification on 180C**	**0% HSA**	pEC_{50}±SD	n	**0.1% HSA**	pEC_{50}±SD	n	**1.0% HSA**	pEC_{50}±SD	n	**1.5% HSA**	pEC_{50}±SD	n
**1**	-1M		NA			NA			NA			NA		
**4**	-1A, 121Q, 168L		2.0	8.7 ± 0.2	23	1.4	8.9 ± 0.3	23	0.9	9.0 ± 0.1	3	1.5	8.8 ± 0.2	6
**15**	-1A, 121Q, 168L, 180C	C18 sidechain	3.3	8.5 ± 0.2	50	23	7.6 ± 0.2	26	195	6.7 ± 0.03	3	198	6.7 ± 0.2	6
**16**	-1A, 121Q, 168L, 180C	Cysteamine	2.5	8.6 ± 0.2	12	1.8	8.7 ± 0.3	10	2.1	8.7 ± 0.2	3	NA		
**20**	-1A, 121Q, 168L, 180C	C12 sidechain	5.8	8.2 ± 0.2	6	3.7	8.4 ± 0.1	3	5.2	8.3 ± 0.1	3	NA		
**21**	-1A, 121Q, 168L, 180C	C14 sidechain	5.2	8.3 ± 0.2	14	4.0	8.4 ± 0.1	8	5.8	8.2 ± 0.1	5	8.6	8.1 ± 0.1	3
**22**	-1A, 121Q, 168L, 180C	C16 sidechain	3.5	8.5 ± 0.3	15	4.2	8.4 ± 0.5	8	52	7.3 ± 0.3	5	92	7.0 ± 0.3	6
**23**	-1A, 121Q, 168L, 180C	C20 sidechain	3.8	8.4 ± 0.2	3	47	7.3 ± 0.6	3	NA			173	6.8 ± 0.4	3

KLB: beta-klotho; NA: not applicable; pEC_{50}: negative logarithm of EC_{50}; SD: standard deviation.

Table S4. Statistical Strength of t½ and In Vivo Potency Data Shown in Table 3.

			t½ (h) 20 mg/kg iv	Body weight loss (g)	
**Compound**	**Mutations**	**Modification on 180C**	**Mice Mean[a]**	**Lean mice Mean ± SEM**	**DIO mice Mean ± SEM**
**1**	-1M		0.7	0.5 ± 0.2[b]	NA
**4**	-1A, 121Q, 168L		1.1	NA	NA
**15**	-1A, 121Q, 168L, 180C	C18 fatty diacid	12.3	2.6 ± 0.2[c]	9.8 ± 0.5[d]
**16**	-1A, 121Q, 168L, 180C	Cysteamine	NA	NA	NA
**20**	-1A, 121Q, 168L, 180C	C12 fatty diacid	1.1	0.6 ± 0.1[b]	NA
**21**	-1A, 121Q, 168L, 180C	C14 fatty diacid	0.9	0.9 ± 0.2[b]	NA
**22**	-1A, 121Q, 168L, 180C	C16 fatty diacid	3.2	1.1 ± 0.2[c]	NA
**23**	-1A, 121Q, 168L, 180C	C20 fatty diacid	23.6	NA	7.0 ± 0.5[d]

[a]t½ presented as harmonic mean without variability, due to plasma concentration versus time profiles being generated using sparse sampling in mice. [b]1 mg/kg sc twice-daily dosing. [c]1 mg/kg sc once-daily dosing. [d]0.3 mg/kg sc once-daily dosing. DIO: diet-induced obese. NA: not applicable; SEM, standard error of the mean; sc: subcutaneous.

Figure S3. Effect of Albumin on In Vitro Potency of FGF21 Compounds with Increasing Length of Fatty Diacid Sidechain.

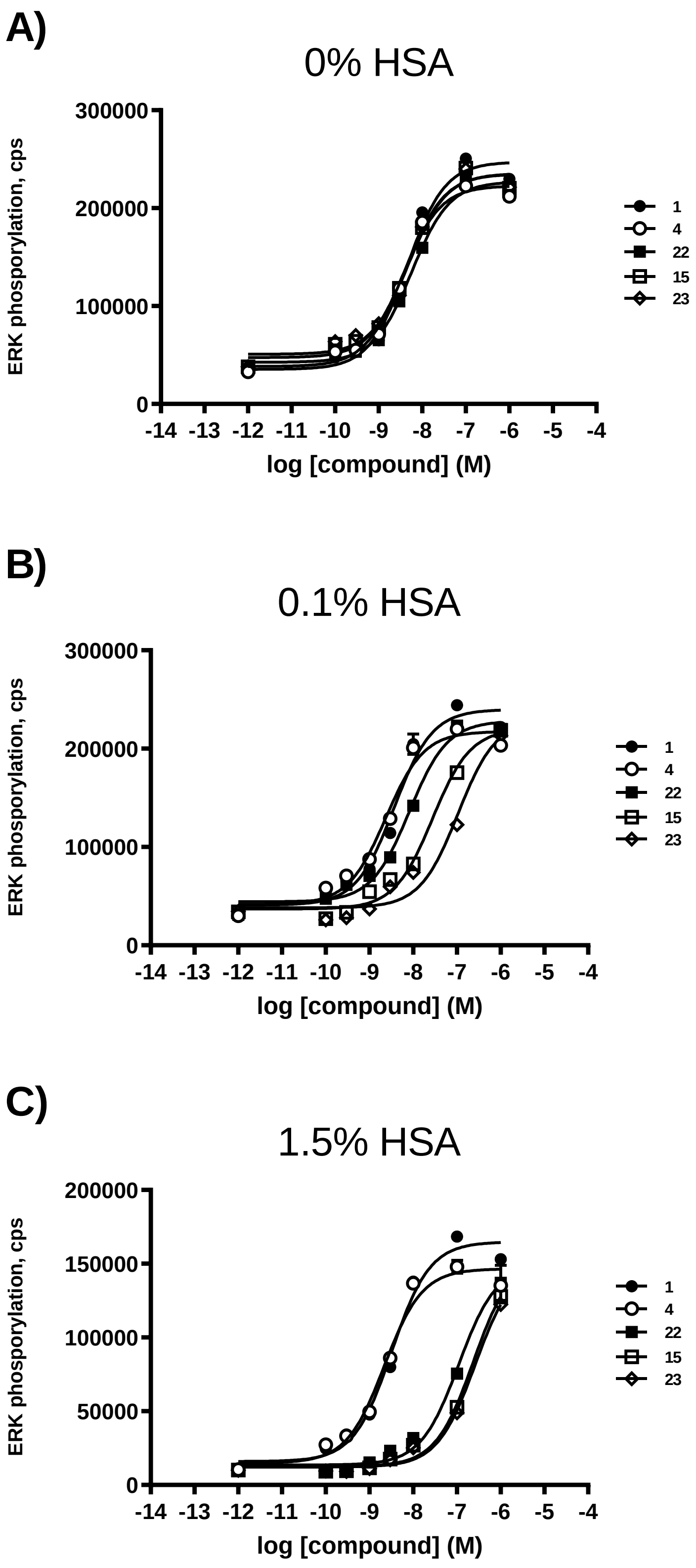

Phosphorylation of ERK in HEK293/KLB Cells in Response to Increasing Concentrations of Met-FGF21 (**1**), **4**, 22 (C16 diacid), **15** (C18 diacid), and **23** (C20 diacid) A) in the absence of HSA; B) in the presence of 0.1% HSA; C) in the presence of 1.5% HSA. KLB: beta-klotho; Met-FGF21: human FGF21 with N-terminal Met extension. Data are shown as mean ± standard error of the mean from a representative assay performed with duplicate measurements. Some error bars coincide with the data points and thus aren't visible.

Figure S4. FGF21-induced ERK-phoshorylation in Ba/F3 cells overexpressing FGFR1c/KLB, FGFR2c/KLB, FGFR3c/KLB, and FGFR4/KLB

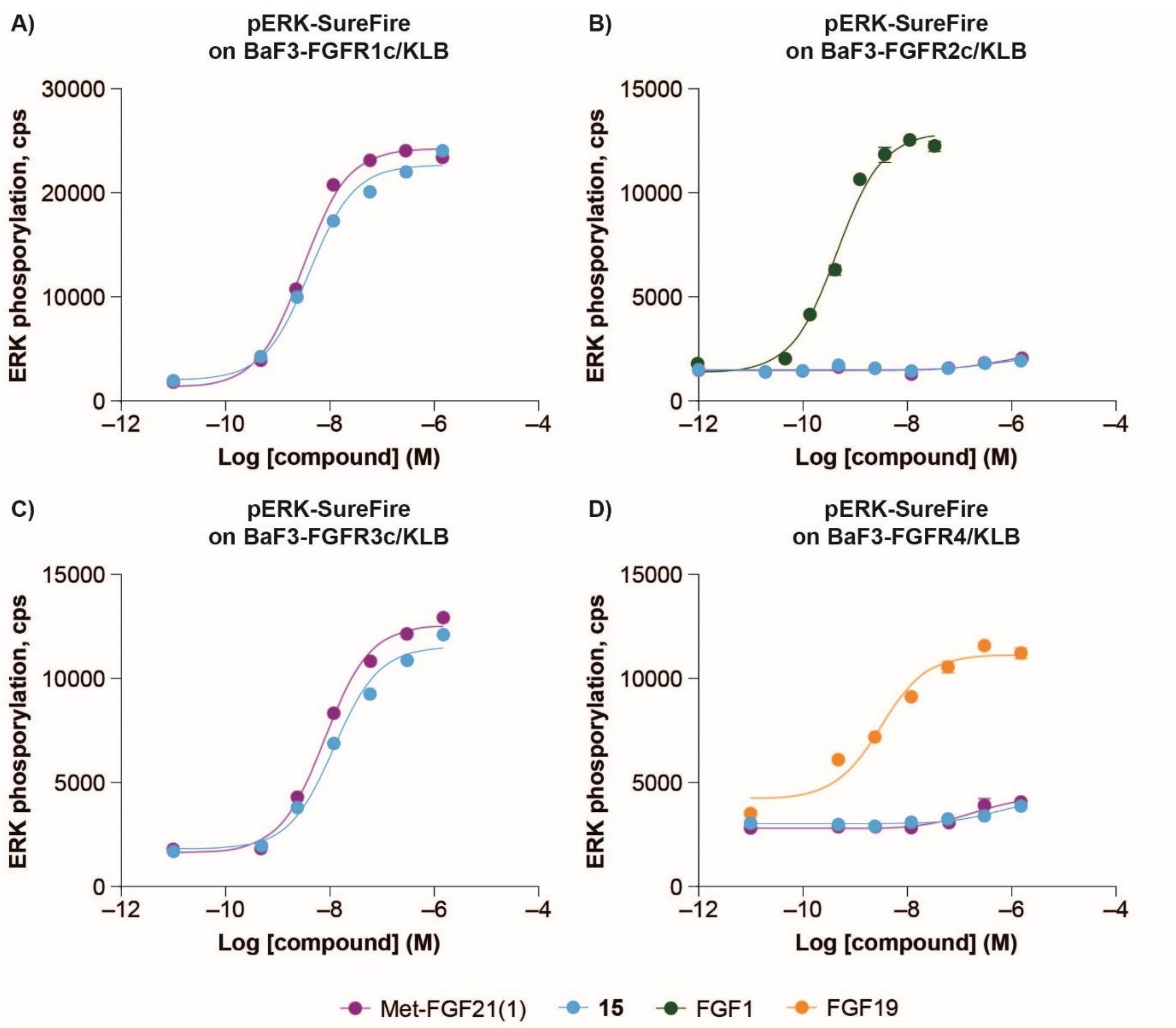

Phosphorylation of ERK in Ba/F3 cells overexpressing FGFR1c/KLB (A), FGFR2c/KLB (B), FGFR3c/KLB (C) and FGFR4/KLB in response to increasing concentrations of Met-FGF21 (**1**), **15**, FGF1, and FGF19. Data are shown as mean ± standard error of the mean (N = 3). Some error bars coincide with the data points and thus aren't visible.

SI E: Lowering of Body Weight in Lean and DIO Mice

Figure S5. Effect of **15** and **23** in DIO mice.

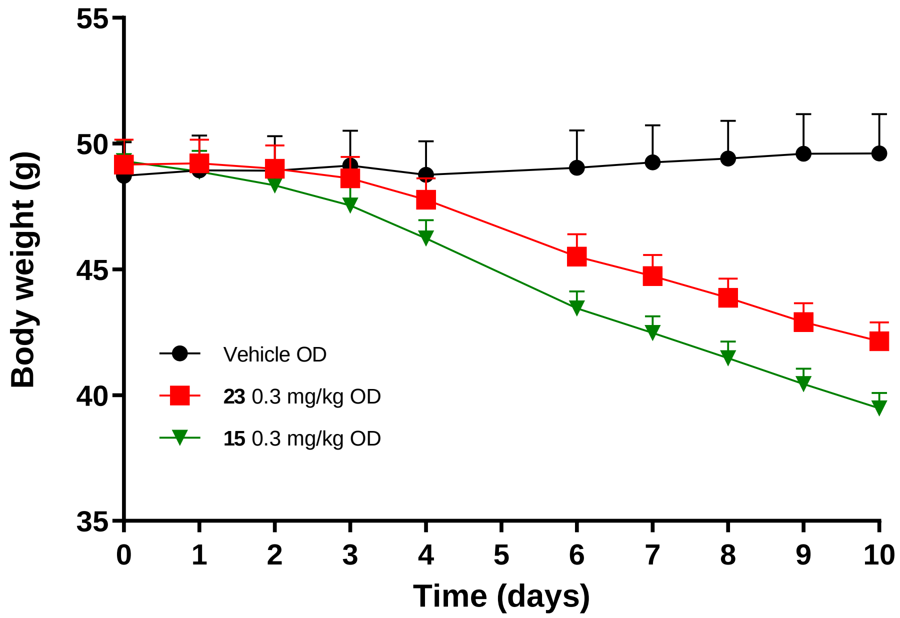

Compounds were dosed 0.3 mg/kg sc once daily. Body weight was measured in the morning before dosing. Data are shown as mean ± standard error of the mean (*n* = 10). DIO: diet-induced obese; OD: once daily.

Figure S6. 15 Dose Response in DIO mice.

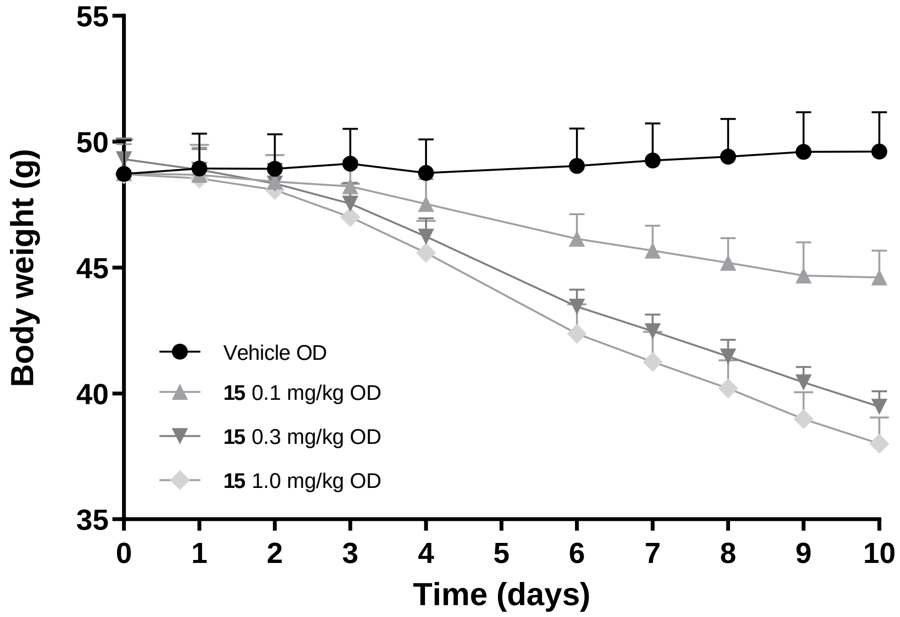

15 was dosed at 0.1 mg/kg, 0.3 mg/kg and 1 mg/kg sc. Body weight was measured in the morning before dosing. Data are shown as mean ± standard error of the mean (n = 10). DIO: diet-induced obese; OD: once daily.

SI F: Proteolytic Degradation of 15 in Mice and Minipig

Table S5. Identification of Metabolites for **15** Following Dosing in Mice (20 mg/kg, iv). The 152–181 metabolite was found at the highest levels in vivo.

Metabolite ID	Observed mass[a]	Mass accuracy (ppm)	Sequence[d]
**15**	20,311.38[a]	2	AHPIPDSSPLLQFGGQV...PLSLVGPSQGRSPSYCS
–1–180	20,224.34[a]	2	AHPIPDSSPLLQFGGQV...DPLSLVGPSQGRSPSYC
172–181	943.9741[b]	0	SQGRSPSYCS
152–181	957.4943[c]	3	ILAPQPPDVGSSDPLSLVGPSQGRSPSYCS

[a]Monoisotopic mass, uncharged from deconvolution. [b]Charge state 2. [c]Charge state 4. [d]Fatty-diacid sidechain on 180Cys residue.

Figure S7. Exposure of **15** and The Most Abundant Metabolite 152–181 in Minipig (1 mg/kg, iv).

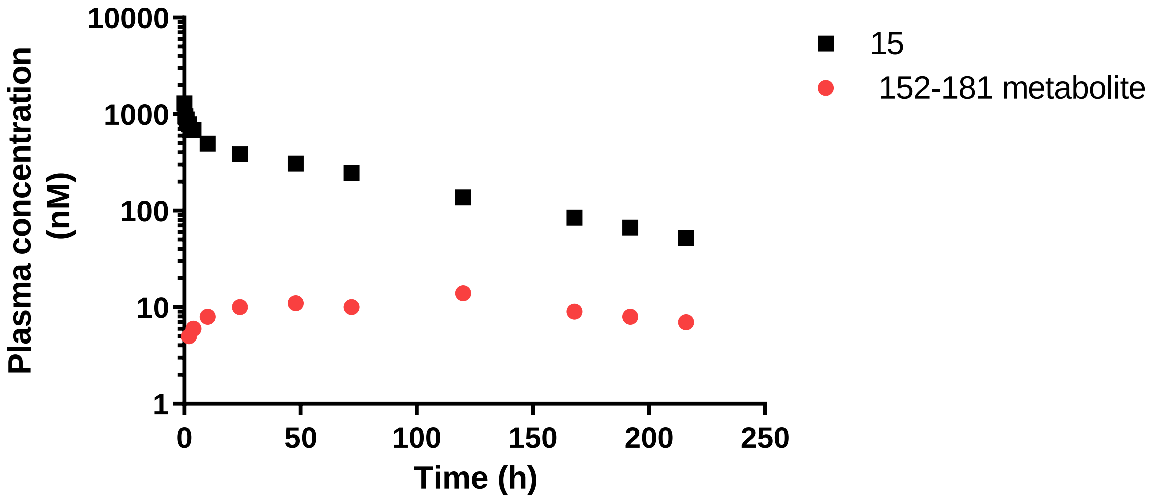

15 was measured by immunoassay and 152–181 metabolite by LC-MS. **15** data are represented as mean data (n = 3) and LC-MS quantification of 152–181 metabolite was conducted on samples pooled at the individual time points (n = 3).

SI G: Identification of Metabolites of 5

Table S6. Identification of Metabolites of **5** Dosed to Mice (20 mg/kg, iv).

The –1–171 metabolite (FAP metabolite) was found at the highest levels in vivo.

Metabolite ID	Observed mass[a]	Mass accuracy (ppm)	Sequence[b]
**5**	20,295.41	0	AHPIPDSSPLLQFGGQV...PLSLVGPSQGRSPSYAS
–1–180	20,208.37	1	AHPIPDSSPLLQFGGQV...DPLSLVGPSQGRSPSYA
–1–179	20,137.33	–1	AHPIPDSSPLLQFGGQV...SDPLSLVGPSQGRSPSY
–1–178	19,974.28	0	AHPIPDSSPLLQFGGQV...SSDPLSLVGPSQGRSPS
–1–171	19,274.99	2	AHPIPDSSPLLQFGGQV...PQPPDVGSSDPLSLVGP
–1–167	18,908.73	0	AHPIPDSSPLLQFGGQV...GILAPQPPDVGSSDPLS
–1–151	17,334.93	0	AHPIPDSSPLLQFGGQV...RFLPLPGLPPALPEPPG

[a]Monoisotopic mass, uncharged. [b]Fatty-diacid sidechain on 71Cys residue.

Figure S8. MS/MS spectra of A) **5** (analog with non-engineered FGF21 C-terminal region), and B) FAP metabolite (–1–171) identified in plasma samples from mice (20 mg/kg, iv).

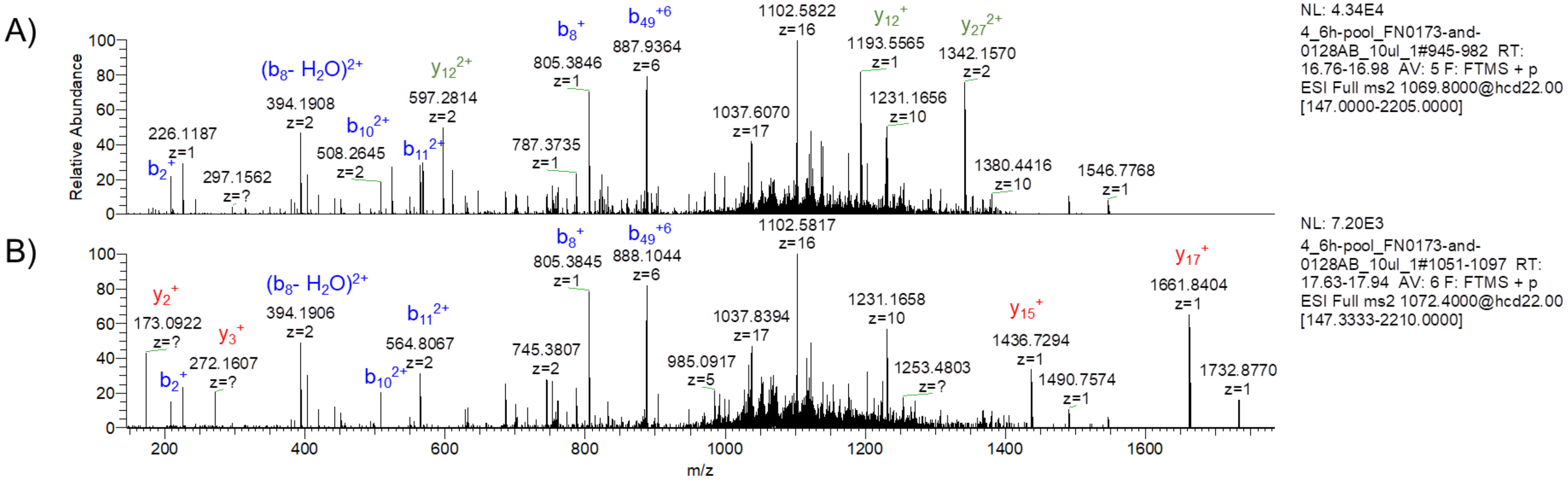

The sample was purified by immunoprecipitation and MS/MS spectra are from LC-MS analysis (PRM). MS/MS was conducted on charge state 19 and charge state 18 of **5** and FAP metabolite, respectively. The b- and y-ions were in accordance with sequences of **5** and the FAP metabolite. FAP: fibroblast activation protein.

SI H: Theoretical Trypsin Peptide Maps of Met-FGF21 (1) and 15

Table S7. Theoretical Trypsin Peptide Map of Met-FGF21 (**1**).

Peptide number	From–To	Mass (Da)[a]	Sequence
T1	–1–17	1,978.01	MHPIPDSSPLLQFGGQVR
T2	18–19	302.17	QR
T3	20–36	2,064.98	YLYTDDAQQTEAHLEIR
T4	37–56	2,013.99	EDGTVGGAADQSPESLLQLK
T5	57–69	1,334.86	ALKPGVIQILGVK
T6	70–72	362.19	TSR
T7	73–96	2,726.24	FLCQRPDGALYGSLHFDPEACSFR
T8	97–122	2,905.46	ELLLEDGYNVYQSEAHGLPLHLPGNK
T9	123–126	495.26	SPHR
T10	127–131	554.28	DPAPR
T11	132–135	399.22	GPAR
T12	136–175	3,997.10	FLPLPGLPPALPEPPGILAPQPPDVGSSDPLSMVGPSQGR
T13	176–181	610.26	SPSYAS

[a]Monoisotopic mass, uncharged. Met-FGF21: human FGF21 with N-terminal Met extension.

Table S8. Theoretical Trypsin Peptide Map of **15**.

Peptide number	From–To	Mass (Da)[a]	Sequence[b]
T1	–1–17	1,918.01	AHPIPDSSPLLQFGGQVR
T2	18–19	302.17	QR
T3	20–36	2,064.98	YLYTDDAQQTEAHLEIR
T4	37–56	2,013.99	EDGTVGGAADQSPESLLQLK
T5	57–69	1,334.86	ALKPGVIQILGVK
T6	70–72	362.19	TSR
T7	73–96	2,726.24	FLCQRPDGALYGSLHFDPEACSFR
T8	97–122	2,919.48	ELLLEDGYNVYQSEAHGLPLHLPGQK
T9	123–126	495.26	SPHR
T10	127–131	554.28	DPAPR
T11	132–135	399.22	GPAR
T12	136–175	3,979.15	FLPLPGLPPALPEPPGILAPQPPDVGSSDPLSLVGPSQGR
T13	176–181	1,457.72	SPSYCS

[a]Monoisotopic mass, uncharged. [b]Fatty-diacid sidechain on 180Cys residue.

SI I: Compound Purity Data

Table S9. Purity of FGF21 Analogs as Determined by RP-UPLC.

Compound	Purity (RP-UPLC) (%)
**1**	98.3
**4**	93.9
**15**	94.3
**16**	86.6[a]
**20**	97.6
**21**	95.8
**22**	93.7
**23**	91.8

[a]Also contained 6.6% S-S dimer linking two monomers of **16** at the introduced 180Cys. RP-UPLC: reverse-phase ultra-performance liquid chromatography.

Table S10. Purity of FGF21 Analogs as Determined by SE-HPLC.

Compound	Purity (SE-HPLC) (%)
**1**	100
**4**	99.8
**15**	99.9
**16**	93.0[a]
**20**	99.9
**21**	99.5
**22**	99.8
**23**	99.0

All compounds, except **16**, were tested in vivo.
[a]Sample also contained approximately 6.6% S-S dimer linking two monomers of **16** at the introduced 180Cys (RP-UPLC and LC-MS).

SI J: RP-UPLC and SE-HPLC Traces of 1, 4, 15, 16, 20, 21, 22, and 23

Figure S9. Chromatograms for FGF21 Analog Purity (RP-UPLC) Determinations shown in Table S9.

1

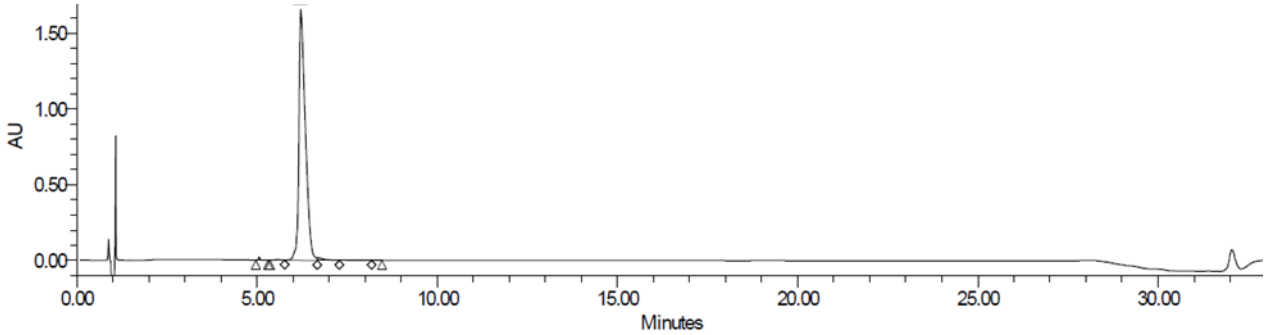

4

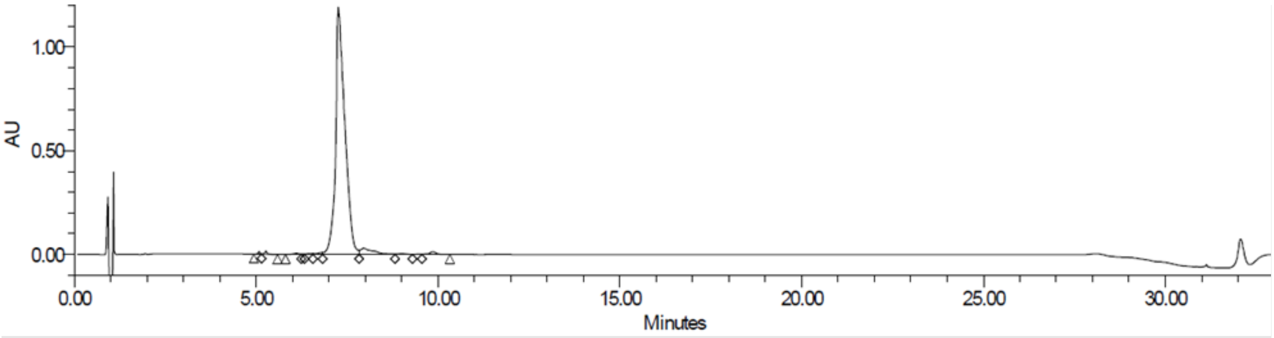

15

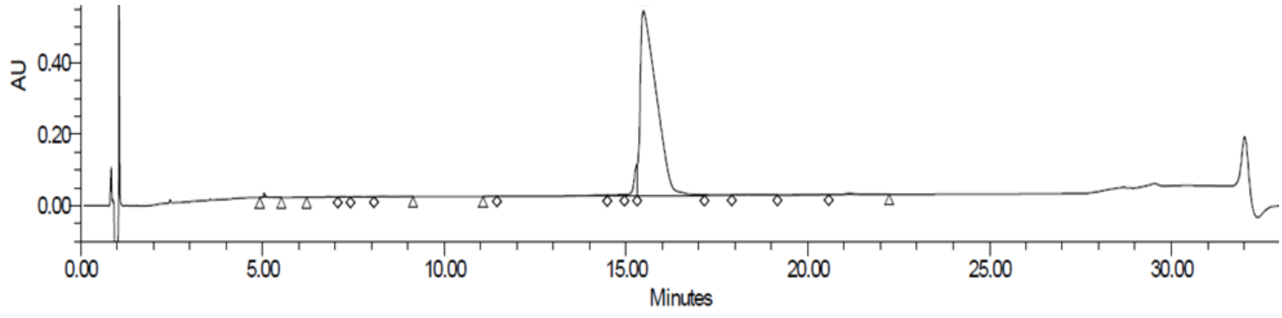

16

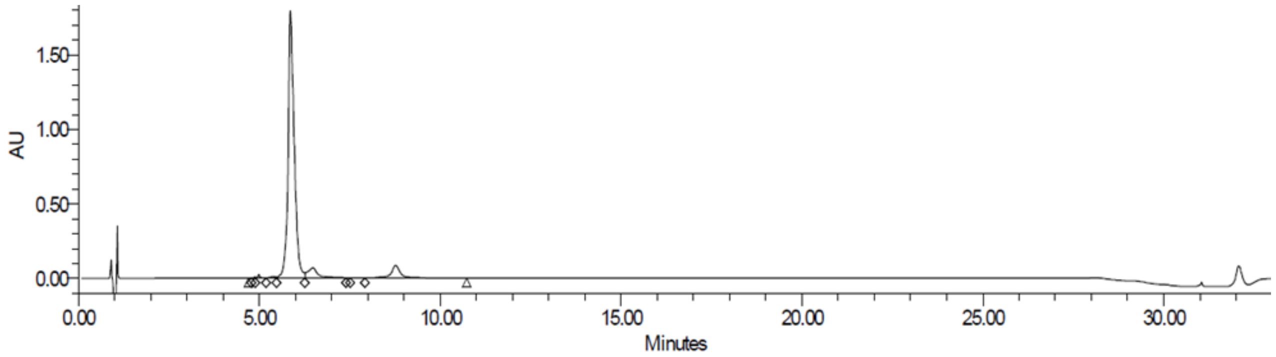
AU
1.50
1.00
0.50
0.00
0.00
5.00
10.00
15.00
20.00
25.00
30.00
Minutes

20

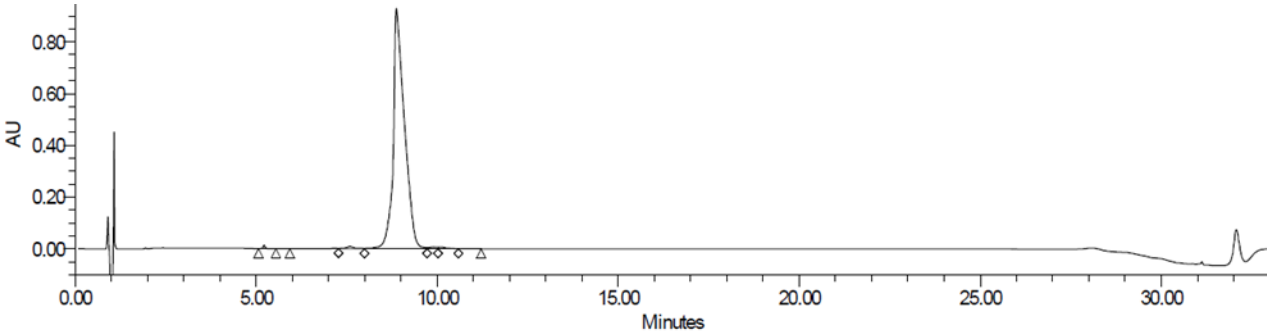
AU
0.80
0.60
0.40
0.20
0.00
0.00
5.00
10.00
15.00
20.00
25.00
30.00
Minutes

21

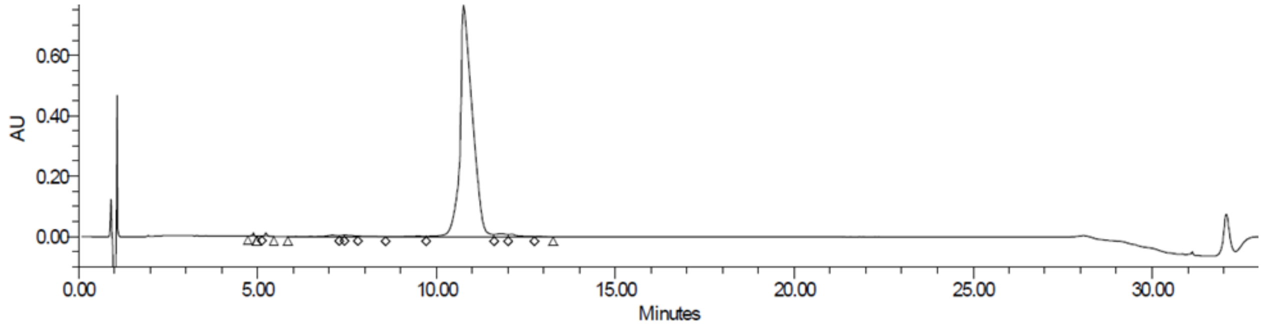
AU
0.60
0.40
0.20
0.00
0.00
5.00
10.00
15.00
20.00
25.00
30.00
Minutes

22

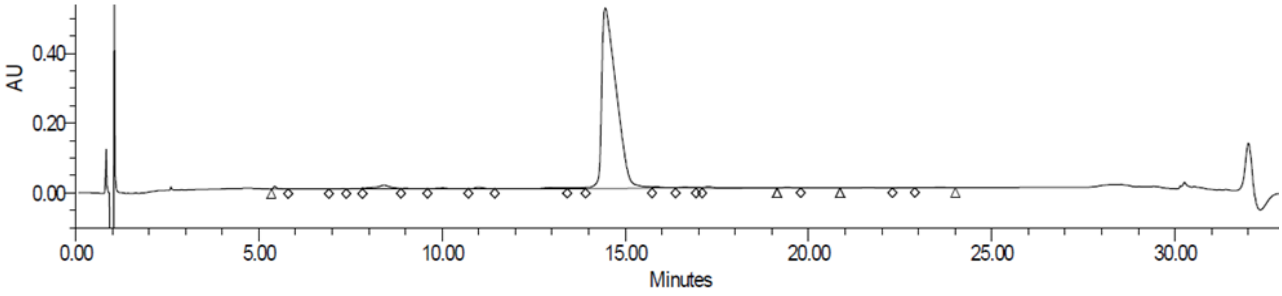
AU
0.40
0.20
0.00
0.00
5.00
10.00
15.00
20.00
25.00
30.00
Minutes

23

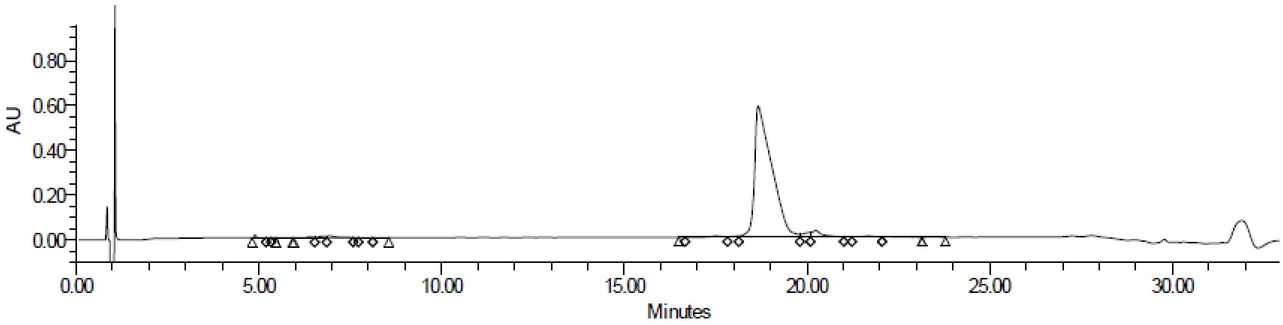

RP-UPLC: reverse-phase ultra-performance liquid chromatography.

Figure S10. Chromatograms for FGF21 Analog Purity (SE-HPLC) Determinations shown in Table S10.

1

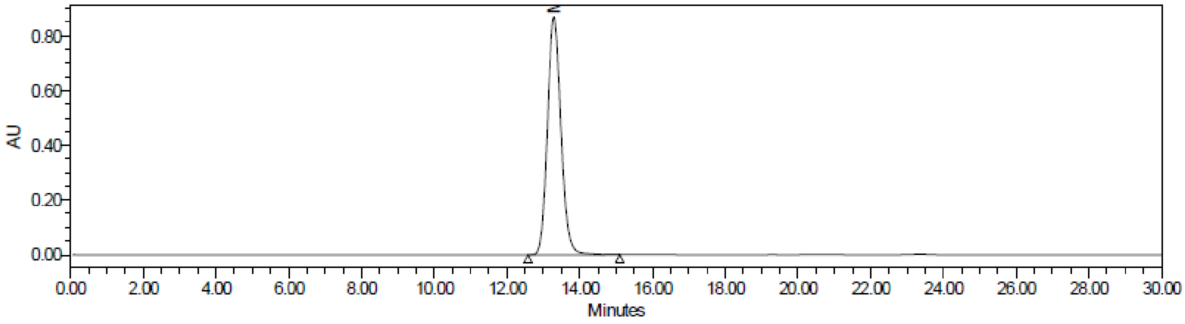

4

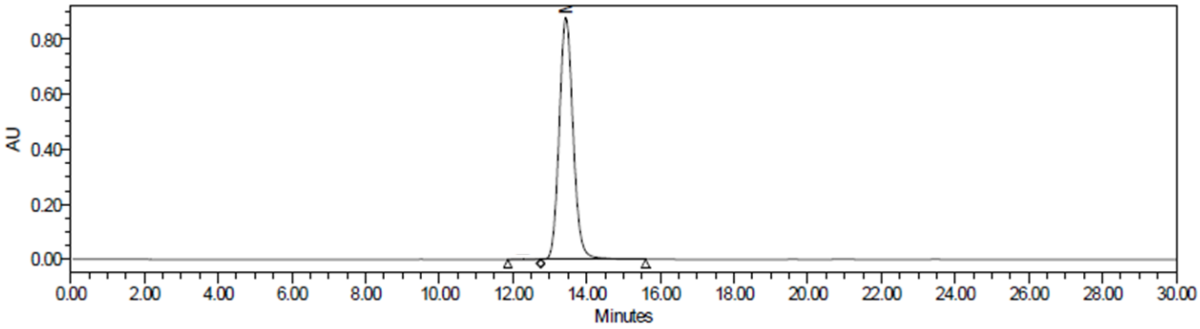

15

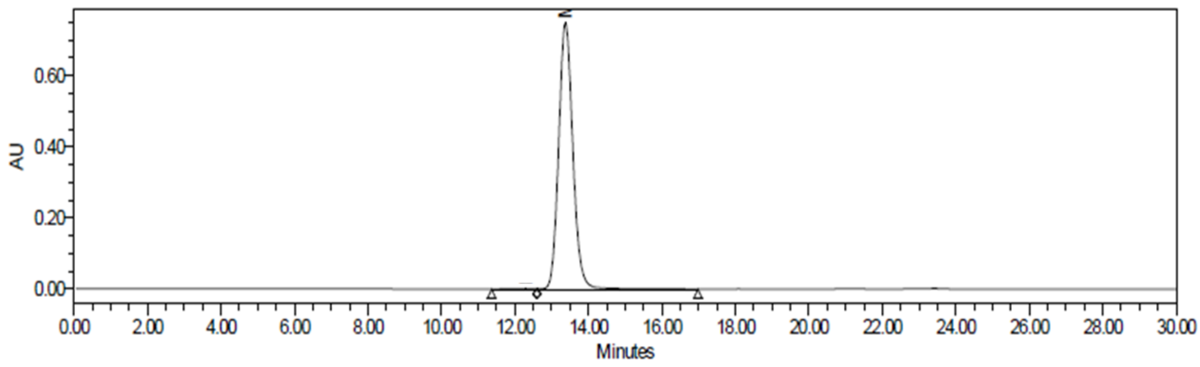
AU
0.00
0.20
0.40
0.60
Minutes
0.00 2.00 4.00 6.00 8.00 10.00 12.00 14.00 16.00 18.00 20.00 22.00 24.00 26.00 28.00 30.00

16

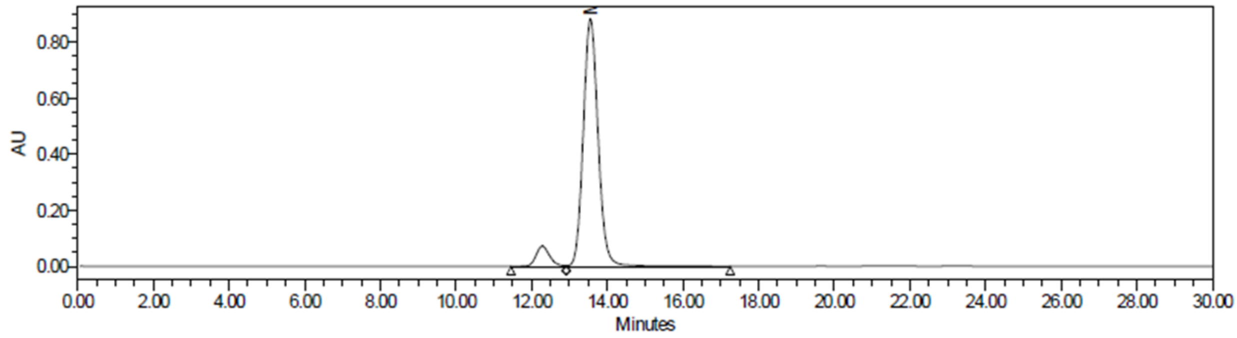
AU
0.00
0.20
0.40
0.60
0.80
Minutes
0.00 2.00 4.00 6.00 8.00 10.00 12.00 14.00 16.00 18.00 20.00 22.00 24.00 26.00 28.00 30.00

20

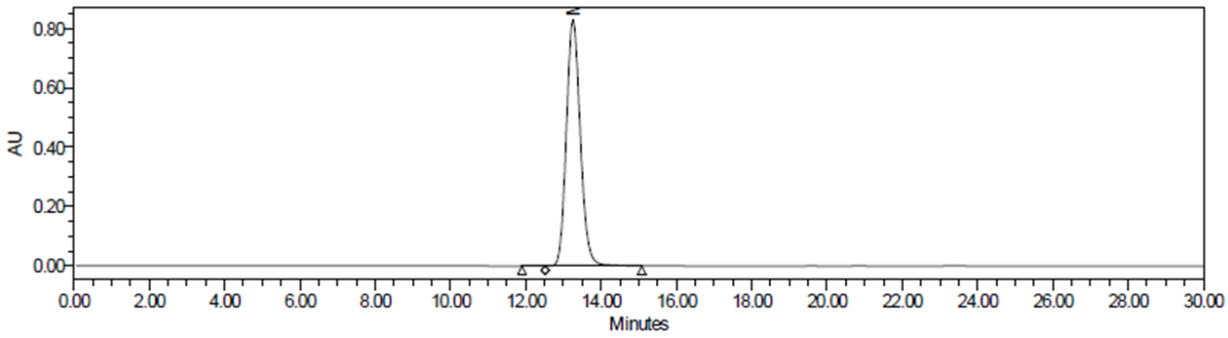
AU
0.00
0.20
0.40
0.60
0.80
Minutes
0.00 2.00 4.00 6.00 8.00 10.00 12.00 14.00 16.00 18.00 20.00 22.00 24.00 26.00 28.00 30.00

21

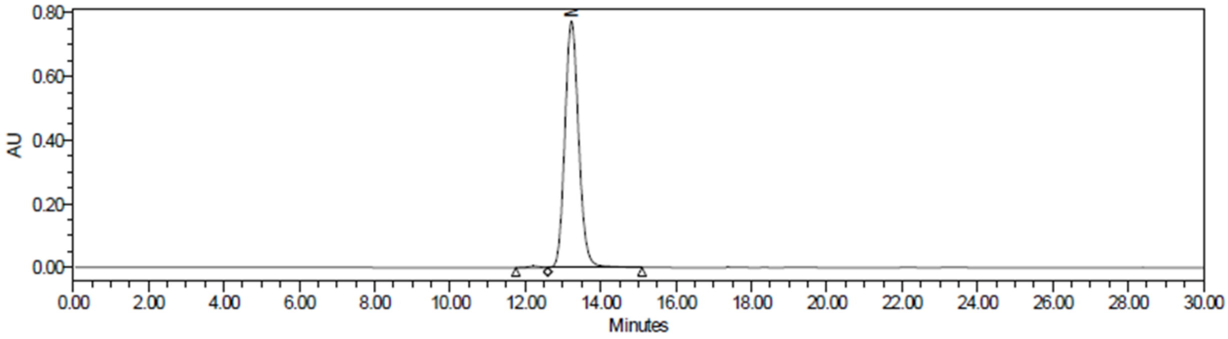
AU
0.00
0.20
0.40
0.60
0.80
Minutes
0.00 2.00 4.00 6.00 8.00 10.00 12.00 14.00 16.00 18.00 20.00 22.00 24.00 26.00 28.00 30.00

22

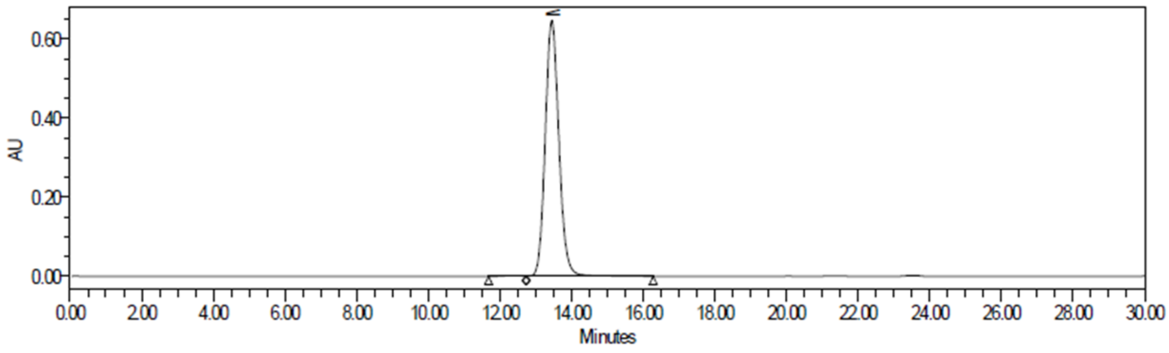

23*

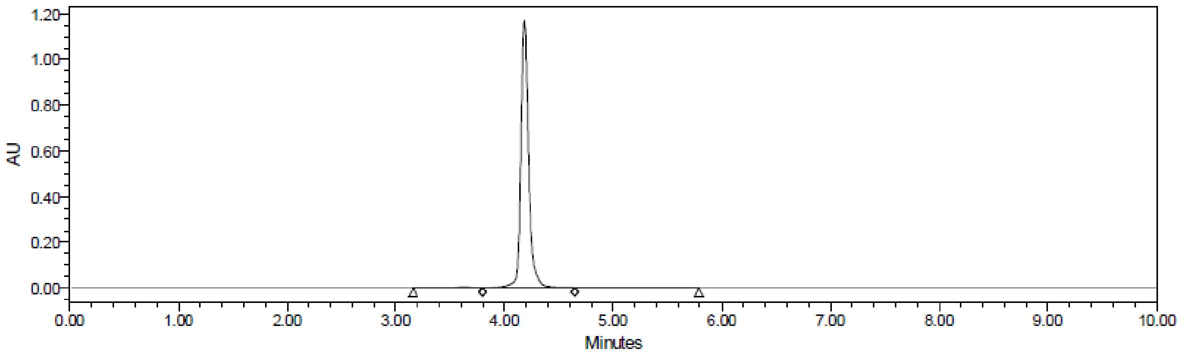

*Only 10-min runtime SE-HPLC data were available for **23**.

SE-HPLC: size-exclusion high-performance liquid chromatography.

SI K: Supplementary Methods

Scheme S1. Albumin-Binding Sidechains: C12, C14, C16, C18, and C20-gGlu-OEG-OEG-C2DA-Ac–Br.

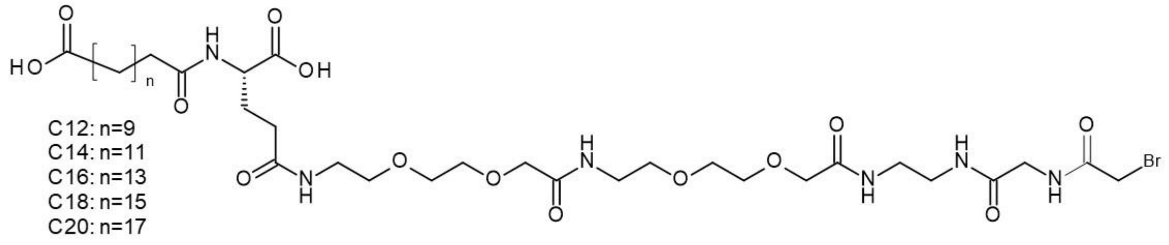

Scheme S2. Synthesis of Sidechains.

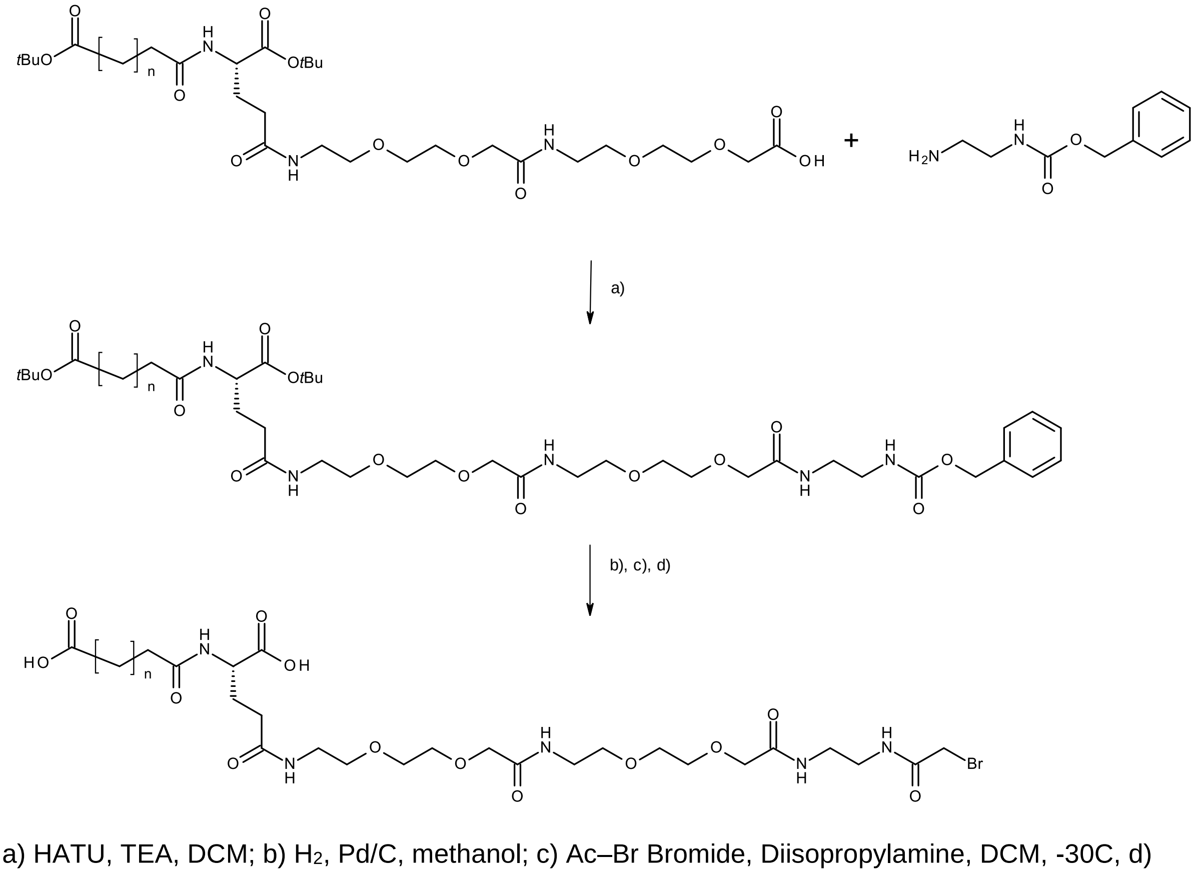

a) HATU, TEA, DCM; b) H_2, Pd/C, methanol; c) Ac–Br Bromide, Diisopropylamine, DCM, -30C, d) TFA. HATU, 2-(7-Aza-1H-benzotriazole-1-yl)-1,1,3,3-tetramethyluronium hexafluorophosphate; TEA: Triethylamine.

Method S1. Generation of HEK293 KLB Cell Lines

HEK293 cells have a native expression of FGF receptors (mainly FGFR1c and FGFR3c) and become responsive to FGF21 when transfected with beta-klotho (KLB).. Expression vector pcDNA3 (Life Technologies) with cDNA encoding human (GenBank: KAI4025177.1), cynomolgus (GenBank: EHH53620.1), and mouse KLB (GenBank: EDL37735.1) was used for transfection. Three separate stable cell lines were generated using standard methods of transfection.

Method S2. In Vitro Potency Assay Description in HEK293

The HEK293/KLB (mouse, cynomolgus, human) cells were seeded with 30,000 cells/well in 96 well plates in Dulecco's Modified Eagle Medium (DMEM) (f BE12-604F/U1, BioWhittaker), supplemented with 10% Fetal Calf Serum (FCS) (cat no. 16140-071 Gibco), 1% penicillin/streptomycin (cat no. 15140, Gibco), 100 µg/mL hygromycin B, (cat no. 400052, Calbiochem). Two days later and 2 h before the addition of the compound, the cell medium was exchanged with 100 µL basal medium (DMEM cat no. BE12-604F/U1, BioWhittaker). The compounds were diluted in assay medium (DMEM cat no. BE12-604F/U1, BioWhittaker) supplemented with 0.02% Tween20, warmed to 37 °C, added to the cells (100 µL), and incubated at 37 °C for 12 min. To test the effect of albumin on the potency, HSA was added to the media in final concentration of 0.1, 1, and 1.5%. All medium was quickly removed and 50 µL lysis-buffer was added per well. The plate was shaken for 5 min and the lysate was transferred to new plates for phosphorylation of extracellular signal-regulated kinase (pERK) analysis. pERK was measured in 384 well plates with the AlphaScreen® SureFire® kit (cat no. TGRES10K, PerkinElmer). The kit is based on ERK and pERK specific antibodies coupled to donor and acceptor beads. The phosphorylation of ERK will bring acceptor and donor beads in close proximity and a signal is generated which is read on an EnVision® plate reader. The data were analysed with non-linear regression of sigmoidal dose-response curves using GraphPad Prism v 6.0 (GraphPad software, La Jolla, CA, USA).

Method S3. Generation of Ba/F3 Cell Lines Expressing KLB/FGFR1c, KLB/FGFR2c, KLB/FGFR3c, and KLB/FGFR4

BaF3 cells lack native expression of FGF receptors and are therefore well suited for investigation of the interaction with specific FGF receptors variants. cDNA encoding human BKL was cloned into vector pMX-IRES-neo and Ba/F3 cells were stably transfected using standard methods. cDNA encoding human FGFR1c, FGFR2c, FGFR3c or FGFR4 was cloned into vector pMX-IRES-puro (#RTV-14, Cell Biolabs) to generate expression vectors. Subsequently receptors were cloned into the KLB containing Ba/F3 cells to generate four separate cell lines representing each receptor subtype. For FGFR3c and FGFR4 the assay signal to noise window was small and to enhance signalling, the extracellular domain of human FGFR3c and FGFR4 sequence was fused to the intracellular domain (tyrosine kinase domain) of FGFR1c to make FGFR3c and FGFR4 chimer receptors.

DNA sequences encoding human KLB, FGFR1c, FGFR2c, FGFR3c (chimer), and FGFR4 (chimer) are shown below.

**Receptor**	**DNA sequence**
KLB	atgaagccaggctgtgcggcaggatctccagggaatgaatggattttcttcagcactgatgaaataaccacacgctata<br>ggaatacaatgtccaacgggggattgcaaagatctgtcatcctgtcagcacttattctgctacgagctgttactggattctc<br>tggagatggaagagctatatggtctaaaaatcctaattttactccggtaaatgaaagtcagctgtttctctatgacactttcc<br>ctaaaaactttttctggggtattgggactggagcattgcaagtggaagggagttggaagaaggatggaaaaggacctt<br>ctatatgggatcatttcatccacacacaccttaaaaatgtcagcagcacgaatggttccagtgacagttatatttttctgga<br>aaaagacttatcagccctggattttataggagtttctttttatcaattttcaatttcctggccaaggcttttccccgatggaatag<br>taacagttgccaacgcaaaaggtctgcagtactacagtactcttctggacgctctagtgcttagaaacattgaacctata<br>gttactttataccactgggatttgcctttggcactacaagaaaaatatgggggtggaaaaatgataccataatagatatc<br>ttcaatgactatgccacatactgtttccagatgtttggggaccgtgtcaaatattggattacaattcacaacccatatctagt<br>ggcttggcatgggtatgggacaggtatgcatgcccctggagagaagggaaatttagcagctgtctacactgtgggaca<br>caacttgatcaaggctcactcgaaagtttggcataactacaacacacatttccgcccacatcagaagggttggttatcg<br>atcacgttgggatctcattggatcgagccaaaccggtcggaaaacacgatggatatattcaaatgtcaacaatccatgg<br>tttctgtgcttggatggtttgccaaccctatccatggggatggcgactatccagaggggatgagaaagaagttgttctccg<br>ttctacccattttctctgaagcagagaagcatgagatgagaggcacagctgatttctttgccttttcttttggacccaacaac<br>ttcaagcccctaaacaccatggctaaaatgggacaaaatgtttcacttaatttaagagaagcgctgaactggattaaac<br>tggaatacaacaaccctcgaatcttgattgctgagaatggctggttcacagacagtcgtgtgaaaacagaagacacca<br>cggccatctacatgatgaagaatttcctcagccaggtgcttcaagcaataaggttagatgaaatacgagtgtttggttata<br>ctgcctggtctctcctggatggctttgaatggcaggatgcttacaccatccgccgaggattattttatgtggattttaacagta<br>aacagaaagagcggaaacctaagtcttcagcacactactacaaacagatcatacgagaaaatggtttttctttaaaag<br>agtccacgccagatgtgcagggccagtttccctgtgacttctcctggggtgtcactgaatctgttcttaagcccgagtctgt<br>ggcttcgtccccacagttcagcgatcctcatctgtacgtgtggaacgccactggcaacagactgttgcaccgagtggaa<br>ggggtgaggctgaaaacacgacccgctcaatgcacagattttgtaaacatcaaaaaacaacttgagatgttggcaag<br>aatgaaagtcacccactaccggtttgctctggattgggcctcggtccttcccactggcaacctgtccgcggtgaaccgac<br>aggccctgaggtactacaggtgcgtggtcagtgaggggctgaagcttggcatctccgcgatggtcaccctgtattatcc<br>gacccacgcccacctaggcctccccgagcctctgttgcatgccgacgggtggctgaacccatcgacggccgaggcct<br>tccaggcctacgctgggctgtgcttccaggagctgggggacctggtgaagctctggatcaccatcaacgagcctaacc<br>ggctaagtgacatctacaaccgctctggcaacgacacctacggggcggcgcacaacctgctggtggcccacgccct<br>ggcctggcgcctctacgaccggcagttcaggccctcacagcgcggggccgtgtcgctgtcgctgcacgcggactggg<br>cggaacccgccaacccctatgctgactcgcactggagggcggccgagcgcttcctgcagttcgagatcgcctggttcg<br>ccgagccgctcttcaagaccggggactaccccgcggccatgagggaatacattgcctccaagcaccgacgggggct<br>ttccagctcggccctgccgcgcctcaccgaggccgaaaggaggctgctcaagggcacggtcgacttctgcgcgctca<br>accacttcaccactaggttcgtgatgcacgagcagctggccggcagccgctacgactcggacagggacatccagttt<br>ctgcaggacatcacccgcctgagctcccccacgcgcctggctgtgattccctgggggggtgcgcaagctgctgcggtgg<br>gtccggaggaactacggcgacatggacatttacatcaccgccagtggcatcgacgaccaggctctggaggatgacc

	ggctccggaagtaccacctagggaagtaccttcaggaggtgctgaaagcatacctgattgataaagtcagaatcaaa<br>ggctattatgcattcaaactggctgaagagaaatctaaacccagatttggattcttcacatctgattttaaagctaaatcctc<br>aatacaattttacaacaaagtgatcagcagcaggggcttccctttgagaacagtagttctagatgcagtcagacccaa<br>gaaaatacagagtgcactgtctgcttattccttgtgcagaagaaaccactgatattcctgggttgttgcttcttctccaccct<br>ggttctactcttatcaattgccatttttcaaaggcagaagagaagaaagttttggaaagcaaaaaacttacaacacatac<br>cattaaagaaaggcaagagagttgttagc
FGFR1c	atgtggagctggaagtgcctcctcttctgggctgtgctggtcacagccacactctgcaccgctaggccgtccccgacctt<br>gcctgaacaagcccagccctggggagcccctgtggaagtggagtccttcctggtccaccccggtgacctgctgcagct<br>tcgctgtcggctgcgggacgatgtgcagagcatcaactggctgcgggacggggtgcagctggcggaaagcaaccg<br>cacccgcatcacaggggaggaggtggaggtgcaggactccgtgcccgcagactccggcctctatgcttgcgtaacc<br>agcagcccctcgggcagtgacaccacctacttctccgtcaatgtttcagatgctctcccctcctcggaggatgatgatgat<br>gatgatgactcctcttcagaggagaaagaaacagataacaccaaaccaaaccccgtagctccatattggacatcccc<br>agaaaagatggaaaagaaattgcatgcagtgccggctgccaagacagtgaagttcaaatgcccttccagtgggacc<br>ccaaaccccacactgcgctggttgaaaaatggcaaagaattcaaacctgaccacagaattggaggctacaaggtcc<br>gttatgccacctggagcatcataatggactctgtggtgccctctgacaagggcaactacacctgcattgtggagaatga<br>gtacggcagcatcaaccacacataccagctggatgtcgtggagcggtcccctcaccggcccatcctgcaagcagggt<br>tgcccgccaacaaaacagtggccctgggtagcaacgtggagttcatgtgtaaggtgtacagtgacccgcagccgcac<br>atccagtggctaaagcacatcgaggtgaatgggagcaagattggcccagacaacctgccttatgtccagatcttgaag<br>actgctggagttaataccaccgacaaagagatggaggtgcttcacttaagaaatgtctcctttgaggacgcaggggag<br>tatacgtgcttggcgggta<br>actctatcggactctcccatcactctgcatggttgaccgttctggaagccctggaagagaggccggcagtgatgacctc<br>gcccctgtacctggagatcatcatctattgcacaggggccttcctcatctcctgcatggtggggtcggtcatcgtctacaa<br>gatgaagagtggtaccaagaagagtgacttccacagccagatggctgtgcacaagctggccaagagcatccctctg<br>cgcagacaggtgtctgctgactccagtgcatccatgaactctggggttcttctggttcggccatcacggctctcctccagt<br>gggactcccatgctagcaggggtctctgagtatgagcttcccgaagaccctcgctgggagctgcctcgggacagactg<br>gtcttaggcaaacccctgggagagggctgctttgggcaggtggtgttggcagaggctatcgggctggacaaggacaa<br>acccaaccgtgtgaccaaagtggctgtgaagatgttgaagtcggacgcaacagagaaagacttgtcagacctgatct<br>cagaaatggagatgatgaagatgatcgggaagcataagaatatcatcaacctgctgggggcctgcacgcaggatgg<br>tcccttgtatgtcatcgtggagtatgcctccaagggcaacctgcgggagtacctgcaggcccggaggcccccagggct<br>ggaatactgctacaaccccagccacaacccagaggagcagctctcctccaaggacctggtgtcctgcgcctaccag<br>gtggcccgaggcatggagtatctggcctccaagaagtgcatacaccgagacctggcagccaggaatgtcctggtga<br>cagaggacaatgtgatgaagatagcagactttggcctcgcacgggacattcaccacatcgactactataaaaagaca<br>accaacggccgactgcctgtgaagtggatggcacccgaggcattatttgaccggatctacacccaccagagtgatgtg<br>tggtctttcggggtgctcctgtgggagatcttcactctgggcggctccccataccccggtgtgcctgtggaggaacttttca<br>agctgctgaaggagggtcaccgcatggacaagcccagtaactgcaccaacgagctgtacatgatgatgcgggactg<br>ctggcatgcagtgccctcacagagacccaccttcaagcagctggtggaagacctggaccgcatcgtggccttgacctc<br>caaccaggagtacctggacctgtccatgcccctggaccagtactcccccagctttcccgacacccggagctctacgtg<br>ctcctcaggggaggattccgtcttctctcatgagccgctgcccgaggagccctgcctgccccgacacccagcccagctt<br>g ccaatggcggactcaaacgccgc
FGFR2c	atggtcagctggggtcgtttcatctgcctggtcgtggtcaccatggcaaccttgtccctggcccggccctccttcagtttagt<br>tgaggataccacattagagccagaagagccaccaaccaaataccaaatctctcaaccagaagtgtacgtggctgcg<br>ccaggggagtcgctagaggtgcgctgcctgttgaaagatgccgccgtgatcagttggactaaggatggggtgcacttg<br>gggcccaacaataggacagtgcttattggggagtacttgcagataaagggcgccacgcctagagactccggcctcta<br>tgcttgtactgccagtaggactgtagacagtgaaacttggtacttcatggtgaatgtcacagatgccatctcatccggaga<br>tgatgaggatgacaccgatggtgcggaagattttgtcagtgagaacagtaacaacaagagagcaccatactggacc<br>aacacagaaaagatggaaaagcggctccatgctgtgcctgcggccaacactgtcaagtttcgctgcccagccgggg<br>ggaacccaatgccaaccatgcggtggctgaaaaacgggaaggagtttaagcaggagcatcgcattggaggctaca<br>aggtacgaaaccagcactggagcctcattatggaaagtgtggtcccatctgacaagggaaattatacctgtgtagtgg<br>agaatgaatacgggtccatcaatcacacgtaccacctggatgttgtggagcgatcgcctcaccggcccatcctccaag<br>ccggactgccggcaaatgcctccacagtggtcggaggagacgtagagtttgtctgcaaggtttacagtgatgcccagc<br>cccacatccagtggatcaagcacgtggaaaagaacggcagtaaatacgggcccgacgggctgccctacctcaagg<br>ttctcaaggccgccggtgttaacaccacggacaaagagattgaggttctctatattcggaatgtaacttttgaggacgctg<br>gggaatatacgtgcttggcgggtaattctattgggatatcctttcactctgcatggttgacagttctgccagcgcctggaag<br>agaaaaggagattacagcttccccagactacctggagatagccatttactgcataggggtcttcttaatcgcctgtatggt<br>ggtaacagtcatcctgtgccgaatgaagaacacgaccaagaagccagacttcagcagccagccggctgtgcacaa<br>gctgaccaaacgtatcccccctgcggagacaggtaacagtttcggctgagtccagctcctccatgaactccaacacccc<br>gctggtgaggataacaacacgcctctcttcaacggcagacacccccatgctggcaggggtctccgagtatgaacttcc<br>agaggacccaaaatgggagtttccaagagataagctgacactgggcaagcccctgggagaaggttgctttgggcaa<br>gtggtcatggcggaagcagtgggaattgacaaagacaagcccaaggaggcggtcaccgtggccgtgaagatgttg<br>aaagatgatgccacagagaaagacctttctgatctggtgtcagagatggagatgatgaagatgattgggaaacacaa

	gaatatcataaatcttcttggagcctgcacacaggatgggcctctctatgtcatagttgagtatgcctctaaaggcaacct<br>ccgagaatacctccgagcccggaggccacccgggatggagtactcctatgacattaaccgtgttcctgaggagcaga<br>tgaccttcaaggacttggtgtcatgcacctaccagctggccagaggcatggagtacttggcttcccaaaaatgtattcat<br>cgagatttagcagccagaaatgttttggtaacagaaaacaatgtgatgaaaatagcagactttggactcgccagagat<br>atcaacaatatagactattacaaaaagaccaccaatgggcggcttccagtcaagtggatggctccagaagccctgttt<br>gatagagtatacactcatcagagtgatgtctggtccttcggggtgttaatgtgggagatcttcactttaggggggctcgccct<br>acccagggattcccgtggaggaactttttaagctgctgaaggaaggacacagaatggataagccagccaactgcac<br>caacgaactgtacatgatgatgagggactgttggcatgcagtgccctcccagagaccaacgttcaagcagttggtaga<br>agacttggatcgaattctcactctcacaaccaatgaggaatacttggacctcagccaacctctcgaacagtattcaccta<br>gttaccctgacacaagaagttcttgttcttcaggagatgattctgtttttctccagaccccatgccttacgaaccatgccttc<br>ctcagtatccacacataaacggcagtgttaaaaca
FGFR3c	atgggcgcccctgcctgcgccctcgcgctctgcgtggccgtggccatcgtggccggcgcctcctcggagtccttgggg<br>acggagcagcgcgtcgtggggcgagcggcagaagtcccgggcccagagcccggccagcaggagcagttggtctt<br>cggcagcggggatgctgtggagctgagctgtcccccgcccgggggtggtcccatggggcccactgtctgggtcaagg<br>atggcacagggctggtgccctcggagcgtgtcctggtggggccccagcggctgcaggtgctgaatgcctcccacgag<br>gactccggggcctacagctgccggcagcggctcacgcagcgcgtactgtgccacttcagtgtgcgggtgacagacg<br>ctccatcctcgggagatgacgaagacggggaggacgaggctgaggacacaggtgtggacacaggggccccttact<br>ggacacggcccgagcggatggacaagaagctgctggccgtgccggccgccaacaccgtccgcttccgctgcccag<br>ccgctggcaaccccactccctccatctcctggctgaagaacggcagggagttccgcggcgagcaccgcattggagg<br>catcaagctgcggcatcagcagtggagcctggtcatggaaagcgtggtgccctcggaccgcggcaactacacctgc<br>gtcgtggagaacaagtttggcagcatccggcagacgtacacgctggacgtgctggagcgctccccgcaccggccca<br>tcctgcaggcggggctgccggccaaccagacggcggtgctgggcagcgacgtggagttccactgcaaggtgtacag<br>tgacgcacagccccacatccagtggctcaagcacgtggaggtgaatggcagcaaggtgggcccggacggcacac<br>cctacgttaccgtgctcaagacggcgggcgctaacaccaccgacaaggagctagaggttctctccttgcacaacgtc<br>acctttgaggacgccggggagtacacctgcctggcgggcaattctattgggttttctcatcactctgcgtggctggtggtg<br>ctgccagccgaggaggagctggtggaggctgacgaggcgggcagtctgtacctggagatcatcatctattgcacagg<br>ggccttcctcatctcctgcatggtggggtcggtcatcgtctacaagatgaagagtggtaccaagaagagtgacttccac<br>agccagatggctgtgcacaagctggccaagagcatccctctgcgcagacaggtgtctgctgactccagtgcatccatg<br>aactctggggttcttctggttcggccatcacggctctcctccagtgggactcccatgctagcaggggtctctgagtatgag<br>cttcccgaagaccctcgctgggagctgcctcgggacagactggtcttaggcaaacccctgggagagggctgctttggg<br>caggtggtgttggcagaggctatcgggctggacaaggacaaacccaaccgtgtgaccaaagtggctgtgaagatgtt<br>gaagtcggacgcaacagagaaagacttgtcagacctgatctcagaaatggagatgatgaagatgatcgggaagcat<br>aagaatatcatcaacctgctgggggcctgcacgcaggatggtcccttgtatgtcatcgtggagtatgcctccaagggca<br>acctgcgggagtacctgcaggcccggaggcccccagggctggaatactgctacaaccccagccacaacccagag<br>gagcagctctcctccaaggacctggtgtcctgcgcctaccaggtggcccgaggcatggagtatctggcctccaagaa<br>gtgcatacaccgagacctggcagccaggaatgtcctggtgacagaggacaatgtgatgaagatagcagactttggcc<br>tcgcacgggacattcaccacatcgactactataaaaagacaaccaacggccgactgcctgtgaagtggatggcacc<br>cgaggcattatttgaccggatctacacccaccagagtgatgtgtggtctttcggggtgctcctgtgggagatcttcactctg<br>ggcggctccccataccccggtgtgcctgtggaggaacttttcaagctgctgaaggagggtcaccgcatggacaagcc<br>cagtaactgcaccaacgagctgtacatgatgatgcgggactgctggcatgcagtgccctcacagagacccaccttca<br>agcagctggtggaagacctggaccgcatcgtggccttgacctccaaccaggagtacctggacctgtccatgcccctg<br>gaccagtactcccccagctttcccgacacccggagctctacgtgctcctcaggggaggattccgtcttctctcatgagcc<br>gctgcccgaggagccctgcctgccccgacacccagcccagcttgccaatggcggactcaaacgccgc
FGFR4	atgcggctgctgctggccctgttgggggtcctgctgagtgtgcctgggcctccagtcttgtccctggaggcctctgaggaa<br>gtggagcttgagccctgcctggctcccagcctggagcagcaagagcaggagctgacagtagcccttgggcagcctgt<br>gcgtctgtgctgtgggcgggctgagcgtggtggccactggtacaaggagggcagtcgcctggcacctgctggccgtgt<br>acggggctggagggggccgcctagagattgccagcttcctacctgaggatgctggccgctacctctgcctggcacgag<br>gctccatgatcgtcctgcagaatctcaccttgattacaggtgactccttgacctccagcaacgatgatgaggaccccaa<br>gtcccatagggacctctcgaataggcacagttacccccagcaagcaccctactggacacacccccagcgcatggag<br>aagaaactgcatgcagtacctgcggggaacaccgtcaagttccgctgtccagctgcaggcaaccccacgcccacca<br>tccgctggcttaaggatggacaggcctttcatggggagaaccgcattggaggcattcggctgcgccatcagcactgga<br>gtctcgtgatggagagcgtggtgccctcggaccgcggcacatacacctgcctggtagagaacgctgtgggcagcatc<br>cgttataactacctgctagatgtgctggagcggtccccgcaccggcccatcctgcaggccgggctcccggccaacac<br>cacagccgtggtgggcagcgacgtggagctgctgtgcaaggtgtacagcgatgcccagccccacatccagtggctg<br>aagcacatcgtcatcaacggcagcagcttcggagccgacggtttcccctatgtgcaagtcctaaagactgcagacatc<br>aatagctcagaggtggaggtcctgtacctgcggaacgtgtcagccgaggacgcaggcgagtacacctgcctcgcag<br>gcaattccatcggcctctcctaccagtctgcctggctcacggtgctgccagaggaggaccccacatggaccgcagca<br>gcgcccgaggccaggtatacggacatcatcatctattgcacaggggccttcctcatctcctgcatggtggggtcggtcat<br>cgtctacaagatgaagagtggtaccaagaagagtgacttccacagccagatggctgtgcacaagctggccaagagc<br>atccctctgcgcagacaggtgtctgctgactccagtgcatccatgaactctggggttcttctggttcggccatcacggctct

	cctccagtgggactcccatgctagcaggggtctctgagtatgagcttcccgaagaccctcgctgggagctgcctcggg<br>acagactggtcttaggcaaacccctgggagagggctgctttgggcaggtggtgttggcagaggctatcgggctggac<br>aaggacaaacccaaccgtgtgaccaaagtggctgtgaagatgttgaagtcggacgcaacagagaaagacttgtca<br>gacctgatctcagaaatggagatgatgaagatgatcgggaagcataagaatatcatcaacctgctgggggcctgcac<br>gcaggatggtcccttgtatgtcatcgtggagtatgcctccaagggcaacctgcgggagtacctgcaggcccggaggcc<br>cccagggctggaatactgctacaaccccagccacaacccagaggagcagctctcctccaaggacctggtgtcctgc<br>gcctaccaggtggcccgaggcatggagtatctggcctccaagaagtgcatacaccgagacctggcagccaggaatg<br>tcctggtgacagaggacaatgtgatgaagatagcagactttggcctcgcacgggacattcaccacatcgactactata<br>aaaagacaaccaacggccgactgcctgtgaagtggatggcacccgaggcattatttgaccggatctacacccacca<br>gagtgatgtgtggtctttcggggtgctcctgtgggagatcttcactctgggcggctccccataccccggtgtgcctgtgga<br>ggaacttttcaagctgctgaaggagggtcaccgcatggacaagcccagtaactgcaccaacgagctgtacatgatga<br>tgcgggactgctggcatgcagtgccctcacagagacccaccttcaagcagctggtggaagacctggaccgcatcgtg<br>gccttgacctccaaccaggagtacctggacctgtccatgcccctggaccagtactcccccagctttcccgacacccgg<br>agctctacgtgctcctcaggggaggattccgtcttctctcatgagccgctgcccgaggagccctgcctgccccgacacc<br>cagcccagcttgccaat ggcggactcaaacgccgc

Method S4. Receptor Selectivity Assay with Ba/F3 Cells

Ba/F3 cell lines were maintained in RPMI 1640 medium (Gibco, cat no. 72400) with 10% heat-inactivated FBS (Ausbian, cat no. VS500T), penicillin/streptomycin (Gibco, cat no. 15140), 0.5 ng/mL IL-3 (Sigma, cat no. 4144), 1 µg/mL puromycin (Gibco, cat no. A11138-03), and 1 mg/mL G418 (Gibco, cat no. 10131-027). The day before the experiment, cells were seeded at 300,000 cells/well with 50 µL phenol-free RPMI1640 (Gibco, cat no. 11835) medium supplemented with 0.02% Tween20 (Merck, cat no. S6386784206) and 10 µg/mL heparin (Sigma, cat no. H3149) in 96-well plates (Falcon, cat no. 353072). The cells were treated by 50 µL of compound at a series of concentrations in assay medium for 15 min at 37 °C. Cells were then lysed with 25 µL of 5xLysis buffer and agitated on a plate shaker (~350 rpm) for 30 min. The lysate was analysed for ERK phosphorylation signal using AlphaScreen® SureFire® kit (PerkinElmer, cat no. TGRES10K) following recommended protocol by the manufacturer. Specifically, 4 µL of the lysate was transferred to a 384-well Proxiplate (PerkinElmer, cat no. 6008289) and mixed with 7µL of reaction mix (60 parts reaction buffer + 10 parts activation buffer + 1 part donor beads + 0.5 part acceptor beads). The plate was sealed with TopSeal-A adhesive film (PerkinElmer, cat no. 6050173), agitated gently on a plate shaker for 5 min, and then incubated at room temperature for 4 h (protected from light) before reading on an EnVision® plate reader (PerkinElmer, cat no. 2104-0010A) with excitation: 680 nm, emission: 520–620 nm setting. The data were analysed with non-linear regression of sigmoidal dose-response curves using GraphPad Prism v 6.0 (GraphPad software, La Jolla, CA, USA).
